# Effect of Different Feeds on the Fungi Microbiome of Suffolk Crossed with Tibetan Sheep

**DOI:** 10.3390/life13112210

**Published:** 2023-11-14

**Authors:** Yue Ren, Renzeng Ciwang, Jia Wang, Khalid Mehmood, Farid Shokry Ataya, Kun Li

**Affiliations:** 1 Institute of Livestock Research, Tibet Academy of Agricultural and Animal Husbandry Sciences, Lhasa 850000, China; 13659501812@163.com; 2 Key Laboratory of Animal Genetics and Breeding on Tibetan Plateau, Ministry of Agriculture and Rural Affairs, Lhasa 850000, China; 3 Institute of Traditional Chinese Veterinary Medicine, College of Veterinary Medicine, Nanjing Agricultural University, Nanjing 210095, China; 17119103@njau.edu.cn (J.W.); lk3005@njau.edu.cn (K.L.); 4 MOE Joint International Research Laboratory of Animal Health and Food Safety, College of Veterinary Medicine, Nanjing Agricultural University, Nanjing 210095, China; 5 Faculty of Veterinary and Animal Sciences, The Islamia University of Bahawalpur, Bahawalpur 6300, Pakistan; khalid.mehmood@iub.edu.pk; 6 Department of Biochemistry, College of Science, King Saud University, P.O. Box 2455, Riyadh 11451, Saudi Arabia; fataya@ksu.edu.sa

**Keywords:** Suffolk crossed with Tibetan sheep, feeds, fungi, microbiome

## Abstract

The gut microbiome plays an important role in the metabolism, nutrient absorption and immunocompetency of animals. The dynamics of the microbiota can be influenced by modulatory factors that involve nutrition, environment, health, diseases, etc. Few reports have been documented regarding the effects of different feeds on the fungi microbiome of Suffolk crossed with Tibetan sheep. A total of 30 Suffolk crossed with Tibetan sheep (ST sheep) were selected for the study and randomly divided into five equal groups (*n* = 6): AZ, BZ, CZ, DZ and EZ. Group AZ was fed with alfalfa and oat grass, whereas group BZ was fed with mixture of concentrated feed, alfalfa and oat grass. Groups CZ, DZ and EZ were fed with concentrated feed #1, #2 and #3, respectively. All experimental animals were fed twice a day for four months, and rectum samples were collected for microbiota analysis. Results revealed that 2,781,461 raw reads and 2,333,239 clean reads were achieved in the ST sheep. When compared with the sheep of groups AZ and BZ (164), the shared amplicon sequence variants (ASVs) between AZ and CZ (109), AZ (113) and DZ (118) as well as AZ along with EZ were fewer. Conspicuous different phyla (8) and genera (56) were examined and compared with free-range sheep in AZ. Genera including *Xeromyces*, *Kazachstania*, *Cordyceps*, *Rhodotorula*, *Pichia*, *Spor,* etc. were found higher in animals in the CZ, DZ and EZ groups. The results of this study provide new insights regarding the effects of different feeds on the fungi microbiome of sheep farmed on the plateau. We concluded that the differences in feed in Suffolk crossed with Tibetan sheep altered their gut microbiota.

## 1. Introduction

Sheep are an important ruminant that provide mutton, milk and cashmere to human beings [1,2]. With the improvement in living standards, there is a growing demand for mutton and its products. The Qinghai–Tibetan Plateau in China is home to many wild and domestic animals [1], of which sheep and yaks are considered important economically, as they provide indispensable elementary resources to local pastoralists [2].

Evolutionary changes in Tibetan sheep have made them adaptable to a harsh environment, as the plateau is has limited forage [3]. Thes plateau sheep not only produce economic and agricultural products but also have significant cultural importance [4,5]. The Suffolk sheep is a worldwide distributed mutton breed, and Suffolk crossed with Tibetan sheep (ST sheep) are popular animals on the plateau because of their fast weight gain and adaptability to the plateau environment.

It has been documented that multiple species of fungi, protozoans and bacteria make up the microbiome [6], which contributes greatly to a host’s metabolism, nutrient absorption and immune status [7]. The abundance of fungi in the gut biome is lower than that of bacteria but still is of significant importance to the health of animal [8]. Previous studies have revealed that colitis and inflammatory bowel and liver diseases are highly co-related to the dysbiosis of fungal microbiota [9,10,11,12]. Microbiota can be influenced by modulatory factors that include nutrition, environment, health, diseases, medications, etc. [13]. Diet plays a crucial role in altering the microbiota by changing the abundance of bacterial species [14]. It has been reported that animals fed with different forages had different gut microbiota [15,16,17]. However, few reports have been documented regarding the effects of different feeds on the fungal microbiome of Suffolk crossed with Tibetan sheep on the plateau. Therefore, the current study aimed to compare the fungal microbiome of Suffolk crossed with Tibetan sheep fed with different feeds.

## 2. Materials and Methods

### 2.1. Experiment Design

A total of thirty (30) three-month-old male Suffolk crossed with Tibetan sheep (ST sheep) with weights of 17.21 ± 1.34 Kg were chosen and divided into five groups, namely AZ, BZ, CZ, DZ and EZ. All the selected sheep were healthy, non-castrated meat animals, and the average weights among all sheep groups were almost same. The ST sheep in group AZ were fed with alfalfa and oat grass; those in group BZ were fed with a mixture of concentrated feed, alfalfa and oat grass; those in groups CZ, DZ and EZ were fed with concentrated feed #1, #2 and #3 (Table S1), respectively. The mixture of concentrated feed in group B was mixed with concentrated feed 1#, 2# and 3# in an equal amount. All the ST sheep were offered feed two times a day for four months, while animals had ad libitum access to clean, fresh drinking water. The animals remained healthy during the experimental period, and no specific treatment strategy was adopted. The type of breeding system of the animals was semi-wild.

The body weight of the ST sheep was measured, and fecal samples from the rectum of all the sheep were collected with cotton swabs and stored in sterile tubes till examination. All samples were transferred to the Institute of Livestock Research of the Tibet Academy of Agricultural and Animal Husbandry Sciences on Drikold and kept at −80 °C in a freezer until further analysis.

### 2.2. Suffolk Cross with Tibetan Sheep Microbiome Analysis

The genomic DNA extraction from each ST sheep group (*n* = 6) were performed using a DNA Stool Mini Kit (QIAamp, Hildan, Germany) according to the guidelines. The quality inspection and quantity detection of the DNA products were carried out through 1.2% agarose gel electrophoresis and a NanoDrop one (Thermo Scientific, Waltham, Massachusetts, USA). The ITS1 region was amplified by using specific primers pairs (ITS1 F:5′-CTTGGTCATTTAGAGGAAGTAA3′; ITS1 R:5′-GCTGCGTTCTTCATCGATGC3′) as per a previous study [18] (Table A2), and the PCR products’ quality and quantity were also examined. Then, library construction was performed by piloting a TIANSeq DirectFast Library Kit (Tiangen Biotech Co., Ltd., Beijing, China). After that, the present library was sent for sequencing via the Illlumina MiSeq platform (Bioyi Biotechnology Co., Ltd., Beijing, China).

The generated raw data from the Illlumina platform were initially passed through quality control DADA2 to achieve the amplicon sequence variants [19]. The ASVs were aligned to the UNITE database (ITS) to produce the taxonomy table [20]. Alpha diversity indexes observed operational taxonomic units (OTUs), and the Chao1, Shannon and Faith’s phylogenetic were calculated using QIIME2. Moreover, the beta diversity indexes, which included principal coordinate analysis, non-metric multidimensional scaling and the unweighted pair group method were measured via arithmetic means. Different fungi among the ST sheep groups were examined through analysis of variance (ANOVA), LEfSe and DEseq2 [21,22]. Function predication of the KEGG Ortholog of the microbiome of the ST sheep was performed through PICRUSt [23].

### 2.3. Statistical Analysis

All the data from the ST sheep were evaluated via ANOVA and Dunn’s test by utilizing IBM SPSS (27.0). Data are presented as the means ± SD and were considered statistically significant at *p* < 0.05.

## 3. Results

### 3.1. Generated Sequencing Data in the ST Sheep in Different Feeding Groups

**Figure 1:**
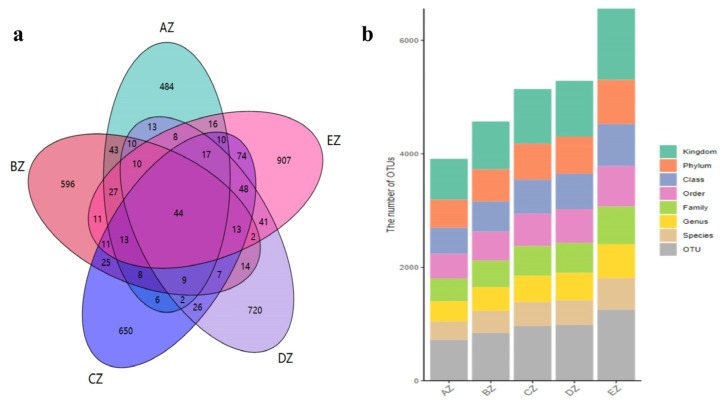
Sequence data analysis: (**a**) Venn map; (**b**) annotation statistics. Groups: AZ, BZ, CZ, DZ and EZ. In (**b**), the different colors represent different taxa.

At the end of this experiment, the body weights of the ST animals in the CZ (*p* < 0.05), DZ (*p* < 0.0001) and EZ (*p* < 0.05) groups were significantly higher than those of the ST sheep in the AZ group (Figure S1). In the present study, a total of 2,781,461 raw data and 2,333,239 filtered data were generated (Table S3). These data produced 3, 577 ASVs, with 44 shared ASVs among the current sheep groups. Group AZ shared 164, 109, 113 and 118 ASVs, respectively, compared with groups BZ, CZ, DZ and EZ (Figure 1a). Then, the ASVs were aligned to different taxonomies, and 15, 15, 16, 14 and 14 phyla were found in the AZ, BZ, CZ, DZ and EZ groups, respectively. A total of 138, 184, 181, 201 and 221 genera were found in the five ST sheep groups, respectively (Figure 1).

### 3.2. Comparing Analyses of the Fungal Microbiota in ST Sheep in Different Feeding Groups

**Figure 2:**
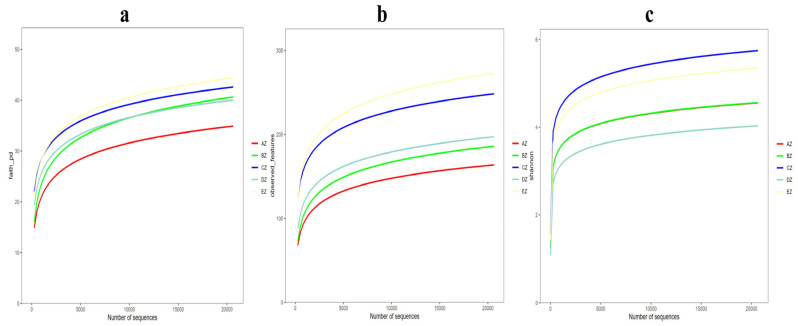
Alpha rarefaction curves for the ST sheep groups: (**a**) faith_pd; (**b**) observed_features; (**c**) shannon_entropy. The different colors represent the different groups of AZ, BZ, CZ, DZ and EZ.

**Figure 3:**
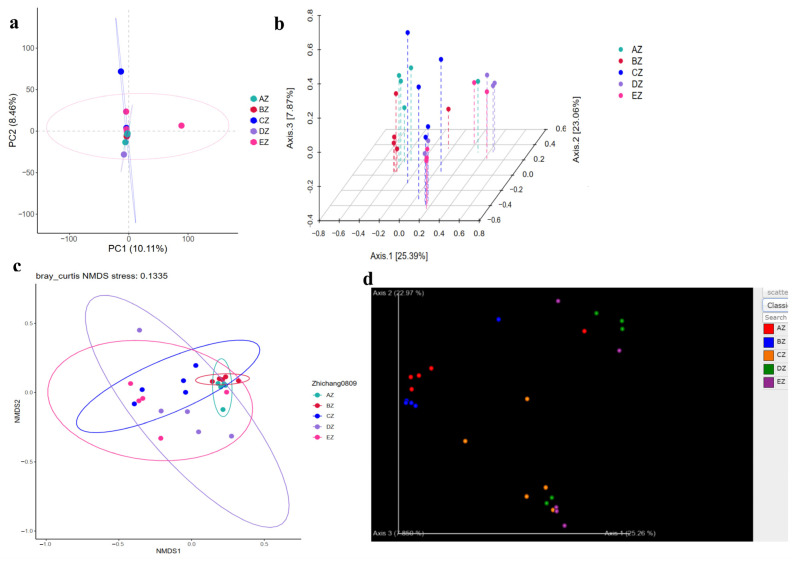
Comparing analyses of the beta diversity index in the ST sheep groups: (**a**) PCA; (**b**) PCoA; (**c**) NMDS; (**d**) Qiime2. The different colors represent the different groups of AZ, BZ, CZ, DZ and EZ.

**Figure 4:**
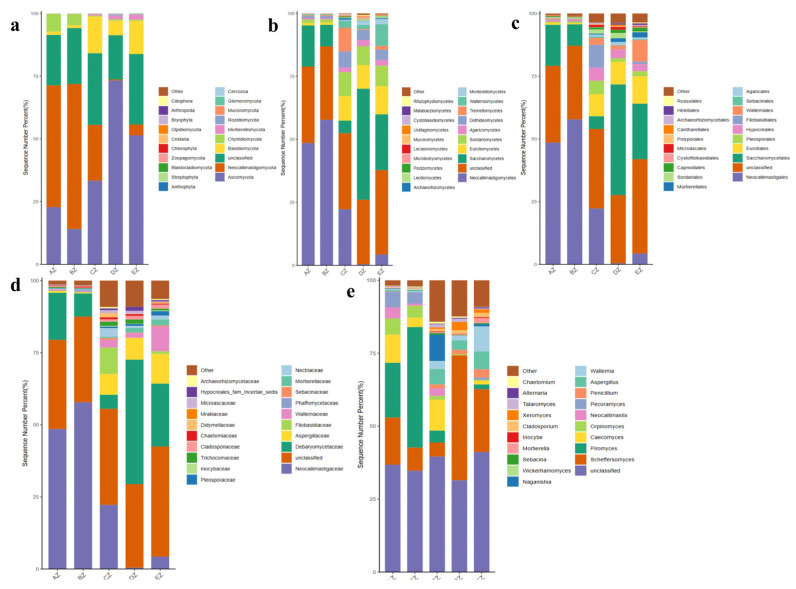
The microbiota structure of the ST sheep in different taxa: (**a**) phylum; (**b**) class; (**c**) order; (**d**) family; (**e**) genus. Groups: AZ, BZ, CZ, DZ and EZ. The different colors represent different species.

Rarefaction curves of faith_pd and features of shannon_entropy were observed in all of the five ST sheep groups with saturated curves, which demonstrated that all the sequencing data were adequate and that the richness and evenness of the microbiota in the ST sheep were sufficient (Figure 2). The Alpha diversity index analysis found that there was no significant difference in the chao1, faith_pd, observed_features, shannon_entropy and Simpson, respectively, among all groups (Table S4, Figure S2). The beta diversity index analysis found that there were no significant differences among the groups through the analyses of PCA and PCoA, whereas significant differences were found via NMDS and Qiime2 (Figure 3). The pairwise ANOSIM analysis found that the distances between the AZ, BZ (*p* < 0.05), CZ (*p* < 0.05), DZ (*p* < 0.01) and EZ (*p* < 0.05) groups were statistically significant. Furthermore, significant differences were found between the BZ, CZ (*p* < 0.05), DZ (*p* < 0.01) and EZ (*p* < 0.01) groups (Figure S3). The primary species in different ST sheep groups for different taxa are shown in Table 1 and Figure 4.

### 3.3. Revealing Marker Fungi Species in ST Sheep in Different Feeding Groups

**Figure 5:**
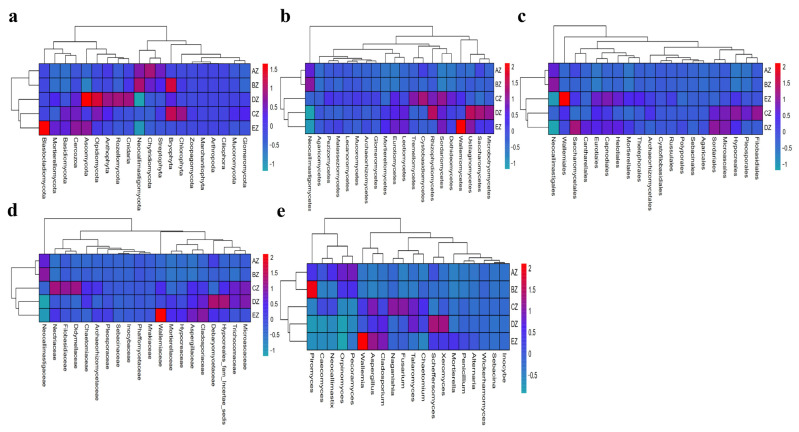
Comparison of the microbiota structures of the ST sheep in different taxa via grouping and a clustering heat map: (**a**) phylum; (**b**) class; (**c**) order; (**d**) family; (**e**) genus.

**Figure 6:**
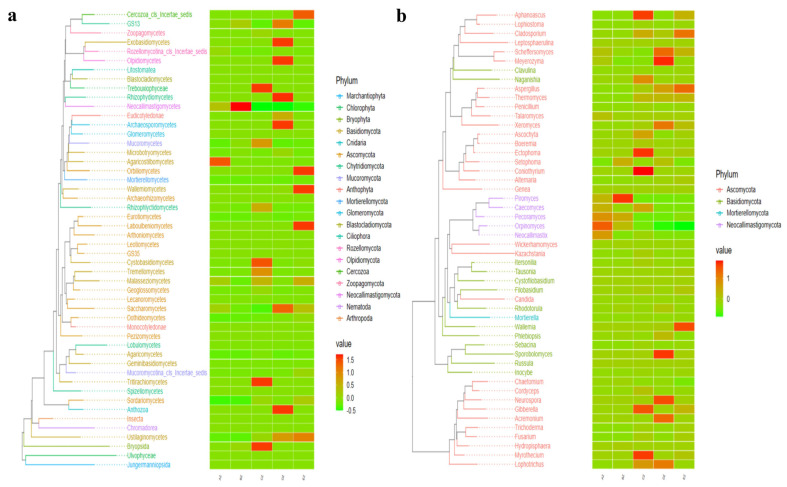
Comparison of the microbiota structure of the ST sheep in different taxa via an evolutionary tree of species with a heat map: (**a**) class; (**b**) genus.

A grouping and clustering heat map analysis found a higher abundance of phyla of Neocallimastigomycota and Chytridiomycota in AZ; Neocallimastigomycota and Bryophyta in BZ; Chlorophyta and Bryophyta in CZ; Ascomycota, Rozellomycota, Anthophyta, Cnidaria and Olpidiomycota in DZ; and Ascomycota, Cercozoa and Blastocladiomycota in EZ (Figure 5a). At the class level, the abundance of Neocallimastigomycetes was higher in AZ and BZ. The abundances of Sordariomycetes, Dothideomycetes, Tremellomycetes, Cystobasidiomycetes and Rhizophydiomycetes were higher in CZ; Saccharomycetes, Sordariomycetes, Microbotryomycetes, Ustilaginomycetes and Rhizophydiomycetes were higher in DZ; and Eurotiomycetes, Sordariomycetes, Wallemiomycetes, Leotiomycetes and Ustilaginomycetes were higher in EZ (Figure 5b). At the order level, Neocallimastigales was higher in AZ and BZ. Higher abundances of Pleosporales, Hypocreales, Filobasidiales, Sordariales and Microascales in CZ; Saccharomycetales, Sordariales, Capnodiales and Cantharellales in DZ; and Eurotiales, Wallemiales, Capnodiales and Helotiales in EZ were observed (Figure 5c). At the family level, Neocallimastigaceae was higher in AZ and BZ. Higher abundances of Filobasidiaceae, Nectriaceae, Trichocomaceae, Didymellaceae and Microascaceae in CZ; Debaryomycetaceae, Trichocomaceae, Cladosporiaceae, Microascaceae and Hypocreales_fam_Incertae_sedis in DZ; and Aspergillaceae, Wallemiaceae and Cladosporiaceae in EZ were observed (Figure 5d). At the genus level, higher abundances of *Orpinomyces* and *Pecoramyces* in AZ; *Piromyces* in BZ; Aspergillus, Naganishia, Talaromyces and Fusarium in CZ; Scheffersomyces, *Cladosporium*, *Xeromyces* and *Talaromyces* in DZ; and Aspergillus, Wallemia and Cladosporium in EZ were detected (Figure 5e). An evolutionary tree of species with a heat map revealed higher abundances of the classes of Agaricostilbomycetes in AZ; Neocallimastigomycetes in BZ; Bryopsida, Tritirachiomycetes and Trebouxiophyceae in CZ; Anthozoa, Archaeosporomycetes, Exobasidiomycetes, Olpidiomycetes, GS13 and Rhizophydiomycetes in DZ; and Ustilaginomycetes, Laboulbeniomycetes, Orbiliomycetes, Cercozoa_cls_Incertae_sedis and Wallemiomycetes in EZ (Figure 6a). At the genus level, higher abundances of *Piromyces* in BZ; *Myrothecium*, *Aphanoascus*, *Ectophoma* and *Coniothyrium* in CZ; *Lophotrichus*, *Acremonium*, Neurospora, Meyerozyma, Scheffersomyces and Xeromyces in DZ; and Cladosporium, *Aspergillus* and *Wallemia* in EZ were found (Figure 6b).

**Figure 7:**
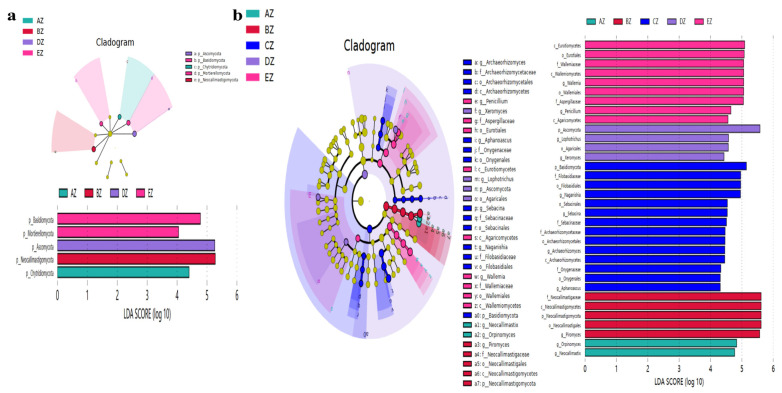
Revealing significant different fungi species among the five ST sheep groups in different taxa via LEfSe. (**a**) Phylum, (**b**) Genera.

**Figure 8:**
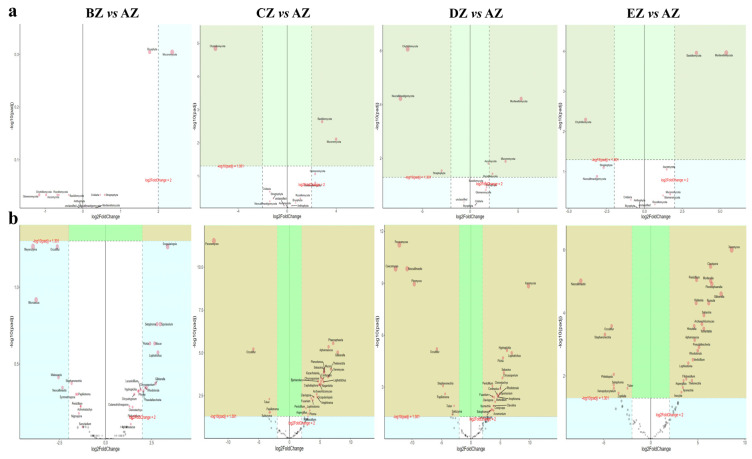
Significant different fungi species among the five ST sheep groups for different taxa according to the DESeq2 volcano map: (**a**) phylum; (**b**) genus.

**Figure 9:**
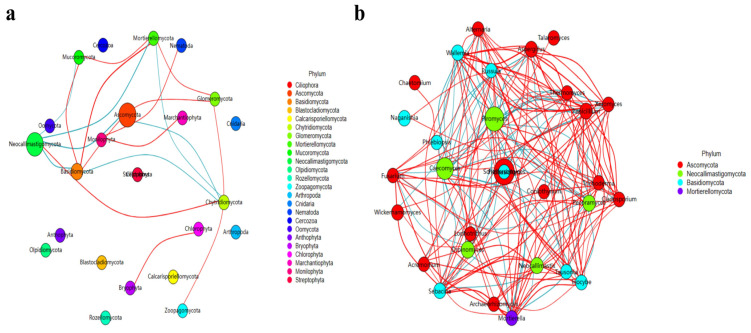
Network analysis of fungi microbiome of the ST sheep for different taxa: (**a**) phylum; (**b**) genus.

A LefSe analysis revealed that five and 34 observably higher abundances of phyla and genus, respectively, were observed among the sheep groups (Table 2, Figure 7). Furthermore, an analysis of the DESeq2 volcano map was performed, and it was found that compared with the AZ group, eight phyla and 56 genera were obviously different in the other ST sheep groups (Table 3, Figure 8). A network analysis showed that the phyla of Basidiomycota, Glomeromycota, Nematoda, Bryophyta and Marchantiophyta were positively related to the microbiota of the ST sheep, while Ascomycota, Mortierellomycota and Mucoromycota were negative phyla (Figure 9a). At the genus level, *Piromyces*, *Caecomyces*, *Penicillium*, *Aspergillus*, *Wallemia*, *Naganishia*, *Sebacina*, *Mortierella*, *Inocybe*, *Cladosporium*, *Xeromyces*, *Talaromyces*, *Alternaria*, *Fusarium*, *Acremonium*, *Archaeorhizomyces*, *Tausonia*, *Trichoderma* and *Thermomyces* were positively related to the fungi microbiota of the ST sheep, while *Scheffersomyces*, *Orpinomyces*, *Neocallimastix* and *Pecoramyces* were negative genera (Figure 9b).

### 3.4. Functional Analysis of Fungal Microbiota in the ST Sheep in Different Feeding Groups

**Figure 10:**
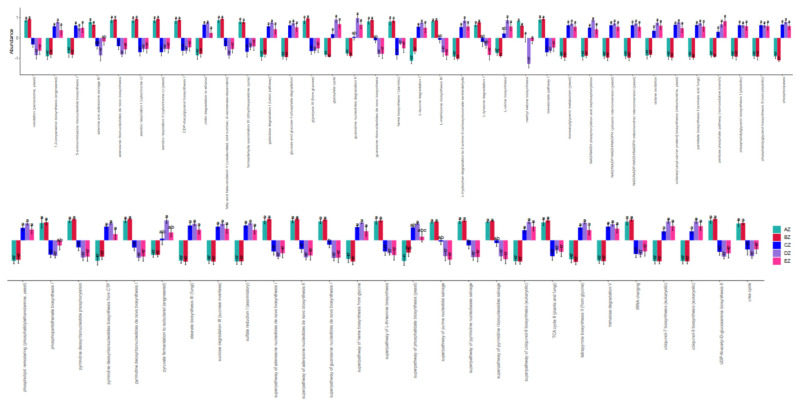
Comparison of the fungi microbiota function of metaCys pathways in the ST sheep for different taxa. Different lowercase letters indicate significant difference.

Function comparison through a metaCys pathways analysis showed that there were 63 significantly different pathways among the five ST sheep groups (Figure 10). An enzyme abundance analysis found that there were 770 markedly different enzymes among the current ST sheep groups (Table S5).

## 4. Discussion

In the plateau region of China, food animals are considered to be important agricultural resources. Sheep are among the popular food animals, with more than 326 million heads that provided 5.25 million tons of mutton in China in 2022 [24]. Therefore, due to an increase in the demand for quality meat in China, efficient farming is of great importance. Gut microbiome analysis is considered to be an indicator of animal health [25]. Similar to bacteria in gut microbiota, fungi also play a vital role in homoeostasis, health status and assembling co-residing bacteria [9].

In this study, we found that the average weights of the ST sheep fed with concentrated feeds were higher, especially in the DZ group (Figure S1). After performing a fungal microbiome analysis of Suffolk crossed with Tibetan sheep with different feeds, 2,781,461 raw reads and 2,333,239 clean reads were obtained (Table 1). Compared with the sheep in groups AZ and BZ (164), the shared ASVs between AZ and CZ (109), AZ (113), DZ (118), and AZ and EZ were found to be fewer (Figure 1), which indicated a difference in the microbiota of the ST sheep fed with concentrated feed. No remarkable differences in the Alpha diversity index were found among the ST sheep groups, which is in accordance with Hu lambs fed with different diets [26] and dogs fed with different diets [27]. However, these results are not in line with those reported in Tibetan sheep fed with different forage types [28]. The current results and previous findings indicate that diet is one of the factors that may affect the richness and diversity of the microbiota. The ANOSIM analysis demonstrated prominent differences among the sheep groups (Figure S2) that were in accordance with the results in dogs fed with different diets [27] and yaks supplemented with dietaries [29]. These findings reveal that diet affects the intestinal microbiota. Structural differences in the microbiota among the five ST sheep groups were revealed through an analysis of a grouped percentage chart (Figure 4), a grouping and clustering heat map (Figure 5) and an evolutionary tree of species with a heat map (Figure 6). Moreover, different fungi species were examined through LEfSe (Figure 7) and a DESeq2 volcano map (Figure 8). The shifted fungi of the microbiota also affected its function (Figure 10 and Table S2).

In the ST sheep, the principal phyla in AZ (48.51%, 22.81%) and BZ (57.76%, 14.09%) were Neocallimastigomycota and Ascomycota, whereas Ascomycota and Neocallimastigomycota were the main phyla in CZ (33.32%, 22.24%). Ascomycota and Basidiomycota were the primary phyla in DZ (73.28%, 5.90%) and EZ (51.40%, 13.26%), respectively (Figure 4a). However, Ascomycota was the dominant phyla in all ST sheep, but its higher abundance was found in CZ, DZ and EZ. These results demonstrated that differences in feed altered the dominant fungi phyla in the sheep, as the fungal mycobiome was less stable and affected by the environment [30,31]. The current results are not in agreement with the results found in yaks using different feed models [32]. The phylum Ascomycota is commonly known due to its function in degrading lignin and keratin, which explains the higher weight gain in the ST sheep in the concentrated feed groups. Among the ST sheep groups eight phyla and 56 genera were examined and compared with free-range sheep in AZ (Figure 8). Among those genera, a higher abundance of *Xeromyces* was reported in healthy children compared with depressed people [33]. The higher abundance of this genus in the CZ, DZ and EZ groups indicates that the concentrated feed promoted ruminant health by increasing the abundance of those fungi. Kazachstania is related to epithelial glycolysis [34], and the higher abundance of this genera in CZ means that concentrated feed #1 promoted glycometabolism in the ST sheep. Species from Cordyceps are widely used in medicines that increase the abundance of useful gut bacteria [35,36]. The abundance of this genera in sheep in the CZ and DZ groups shows that concentrated feeds #1 and #2 promoted sheep health by regulating the intestinal microbiota. Rhodotorula produces nutrients like proteins and vitamins, which benefit host health [37]; the higher abundance of this genera in the DZ and EZ groups revealed that concentrated feeds #2 and #3 promoted the colonization of this useful microflora. *Pichia* and *Sporisorium* are two major genera in healthy humans [31,38], while higher amounts of *Lecanicillium* were found in healthy Tibetan pigs compared with diarrheal animals [39]; the higher abundances of these genera in the DZ groups means that concentrated feed #2 increased the abundance of beneficial fungi in the sheep. A higher abundance of *Mortierella* was detected in healthy yaks compared with ruminants infected with *Cryptosporidium parvum* [40]. A lower abundance of *Plectosphaerella* was examined in fluoride-induced mice [41,42], *Tomentella* was positively related to the generation of short-chain fatty acids [43,44,45] and *Filobasidium* reduced ulcerative colitis [46]; the higher abundance of this genus in the EZ group revealed that concentrated feed #3 promoted sheep health by mediating fungi abundance. The genera of Aphanoascus, Gibberella, *Thelonectria* and *Sebacina* are commonly reported in the environment [47,48,49,50]. However, these genera are not pathogenic, but the abundant changes could affect the microbiota function, which eventually affect host status and weight gains. So, these genera are commonly recognized as beneficial or dominant genera in healthy animals. Therefore, it is recommended that ST sheep may be fed with concentrated feed to increase weight gain through the regulation of fungi.

## 5. Conclusions

Based on the above, we concluded that differences in feed in Suffolk crossed with Tibetan sheep altered their gut microbiota. It was also found that different concentrations of phyla (eight) and genera (56) were observed among the different groups. Higher abundances of the phyla Ascomycota and the genera *Kazachstania* and *Rhodotorula* were found in the ST sheep fed with concentrated feed, which revealed that the concentrated feeds promoted weight gain in the sheep through the regulation of fungi. The results of this study may provide new insights regarding sheep farming on the plateau in China.

## Figures and Tables

**Table 1:** The main species in different ST sheep groups for different taxa.

Taxonomy	Group	Species	Percent (%)
Phylum	AZ	Ascomycota	22.81%
	AZ	Neocallimastigomycota	48.51%
	AZ	Chytridiomycota	7.10%
	BZ	Ascomycota	14.09%
	BZ	Neocallimastigomycota	57.76%
	BZ	Chytridiomycota	4.55%
	CZ	Ascomycota	33.32%
	CZ	Neocallimastigomycota	22.24%
	CZ	Basidiomycota	14.57%
	DZ	Ascomycota	73.28%
	DZ	Neocallimastigomycota	4.29%
	DZ	Basidiomycota	5.90%
	EZ	Ascomycota	51.40%
	EZ	Neocallimastigomycota	4.29%
	EZ	Basidiomycota	13.26%
Class	AZ	Neocallimastigomycetes	48.51%
	AZ	Saccharomycetes	16.30%
	AZ	Sordariomycetes	1.36%
	BZ	Neocallimastigomycetes	57.76%
	BZ	Saccharomycetes	8.50%
	BZ	Sordariomycetes	1.23%
	CZ	Neocallimastigomycetes	22.24%
	CZ	Eurotiomycetes	9.73%
	CZ	Sordariomycetes	9.50%
	DZ	Saccharomycetes	44.07%
	DZ	Eurotiomycetes	9.43%
	DZ	Sordariomycetes	7.43%
	EZ	Saccharomycetes	22.15%
	EZ	Eurotiomycetes	11.17%
	EZ	Sordariomycetes	8.11%
Order	AZ	Neocallimastigales	48.51%
	AZ	Saccharomycetales	16.30%
	AZ	Eurotiales	9.60%
	BZ	Neocallimastigales	57.76%
	BZ	Saccharomycetales	8.50%
	BZ	Pleosporales	0.46%
	CZ	Neocallimastigales	22.24%
	CZ	Filobasidiales	9.15%
	CZ	Eurotiales	8.79%
	DZ	Saccharomycetales	44.07%
	DZ	Eurotiales	9.08%
	DZ	Hypocreales	3.40%
	EZ	Saccharomycetales	22.15%
	EZ	Eurotiales	10.84%
	EZ	Wallemiales	8.59%
Family	AZ	Neocallimastigaceae	48.51%
	AZ	Debaryomycetaceae	16.28%
	AZ	Aspergillaceae	0.59%
	BZ	Neocallimastigaceae	57.76%
	BZ	Debaryomycetaceae	8.02%
	BZ	Phaffomycetaceae	0.37%
	CZ	Neocallimastigaceae	22.24%
	CZ	Filobasidiaceae	9.15%
	CZ	Aspergillaceae	7.25%
	DZ	Debaryomycetaceae	43.14%
	DZ	Aspergillaceae	7.57%
	DZ	Wallemiaceae	1.63%
	EZ	Debaryomycetaceae	21.76%
	EZ	Aspergillaceae	10.26%
	EZ	Wallemiaceae	8.59%
Genera	AZ	*Piromyces*	18.77%
	AZ	*Scheffersomyces*	16.12%
	AZ	*Caecomyces*	9.65%
	BZ	*Scheffersomyces*	7.97%
	BZ	*Orpinomyces*	4.14%
	BZ	*Piromyces*	4.12%
	CZ	*Caecomyces*	10.57%
	CZ	*Naganishia*	9.04%
	CZ	*Aspergillus*	5.29%
	DZ	*Scheffersomyces*	42.80%
	DZ	*Aspergillus*	3.26%
	DZ	*Xeromyces*	2.85%
	EZ	*Scheffersomyces*	21.55%
	EZ	*Wallemia*	8.59%
	EZ	*Aspergillus*	6.12%

**Table 2:** Significant different species among the sheep groups according to LefSe.

Taxonomy	Group	Species	*p*-Value	Significance
Phylum	AZ	Chytridiomycota	<0.01	↑
	BZ	Neocallimastigomycota	<0.01	↑
	DZ	Ascomycota	<0.01	↑
	EZ	Basidiomycota	<0.05	↑
	EZ	Mortierellomycota	<0.05	↑
Genera	AZ	*Orpinomyces*	<0.01	↑
	AZ	*Neocallimastix*	<0.01	↑
	BZ	*Neocallimastigaceae*	<0.01	↑
	BZ	*Neocallimastigomycetes*	<0.01	↑
	BZ	*Neocallimastigales*	<0.01	↑
	BZ	*Neocallimastigomycota*	<0.01	↑
	BZ	*Piromyces*	<0.001	↑
	CZ	*Sebacina*	<0.05	↑
	CZ	*Archaeorhizomycetales*	<0.05	↑
	CZ	*Basidiomycota*	<0.05	↑
	CZ	*Archaeorhizomyces*	<0.05	↑
	CZ	*Archaeorhizomycetes*	<0.05	↑
	CZ	*Sebacinales*	<0.05	↑
	CZ	*Aphanoascus*	<0.05	↑
	CZ	*Onygenaceae*	<0.05	↑
	CZ	*Archaeorhizomycetaceae*	<0.05	↑
	CZ	*Naganishia*	<0.05	↑
	CZ	*Filobasidiales*	<0.05	↑
	CZ	*Sebacinaceae*	<0.05	↑
	CZ	*Filobasidiaceae*	<0.05	↑
	CZ	*Onygenales*	<0.05	↑
	DZ	*Ascomycota*	<0.01	↑
	DZ	*Agaricales*	<0.05	↑
	DZ	*Lophotrichus*	<0.05	↑
	DZ	*Xeromyces*	<0.05	↑
	EZ	*Eurotiales*	<0.05	↑
	EZ	*Eurotiomycetes*	<0.05	↑
	EZ	*Wallemiales*	<0.05	↑
	EZ	*Wallemia*	<0.05	↑
	EZ	*Penicillium*	<0.05	↑
	EZ	*Aspergillaceae*	<0.05	↑
	EZ	*Wallemiomycetes*	<0.05	↑
	EZ	*Wallemiaceae*	<0.05	↑
	EZ	*Agaricomycetes*	<0.01	↑

**Table 3:** Significant different species among the sheep groups according to DESeq2.

Taxonomy	Group	Species	*p*-Value	Significance
Phylum	CZ	Basidiomycota	<0.01	↑
	CZ	Mucoromycota	<0.01	↑
	CZ	Chytridiomycota	<0.0001	↓
	DZ	Mortierellomycota	<0.0001	↑
	DZ	Mucoromycota	<0.05	↑
	DZ	Ascomycota	<0.05	↑
	DZ	Rozellomycota	<0.05	↑
	DZ	Chytridiomycota	<0.0001	↓
	DZ	Neocallimastigomycota	<0.0001	↓
	DZ	Streptophyta	<0.05	↓
	EZ	Basidiomycota	<0.001	↑
	EZ	Mortierellomycota	<0.001	↑
	EZ	Chytridiomycota	<0.001	↓
	EZ	Chytridiomycota	<0.01	↓
Genera	CZ	*Phaeosphaeria*	<0.0001	↑
	CZ	*Aphanoascus*	<0.0001	↑
	CZ	*Gibberella*	<0.0001	↑
	CZ	*Plenodomus*	<0.0001	↑
	CZ	*Thelonectria*	<0.0001	↑
	CZ	*Mucor*	<0.001	↑
	CZ	*Sebacina*	<0.001	↑
	CZ	*Xeromyces*	<0.001	↑
	CZ	*Kazachstania*	<0.001	↑
	CZ	*Cordyceps*	<0.001	↑
	CZ	*Chrysosporium*	<0.001	↑
	CZ	*Bjerkandera*	<0.001	↑
	CZ	*Lophotrichus*	<0.001	↑
	CZ	*Naganishia*	<0.001	↑
	CZ	*Archaeorhizomyces*	<0.001	↑
	CZ	*Cephaliophora*	<0.01	↑
	CZ	*Scopulariopsis*	<0.01	↑
	CZ	*Clavispora*	<0.01	↑
	CZ	*Amphinema*	<0.01	↑
	CZ	*Fusarium*	<0.01	↑
	CZ	*Lophiostoma*	<0.01	↑
	CZ	*Penicillium*	<0.05	↑
	CZ	*Phoma*	<0.05	↑
	CZ	*Aspergillus*	<0.05	↑
	CZ	*Pecoramyces*	<0.001	↓
	CZ	*Occultifur*	<0.001	↓
	CZ	*Tuber*	<0.01	↓
	CZ	*Papiliotrema*	<0.05	↓
	CZ	*Saitozyma*	<0.05	↓
	DZ	*Xeromyces*	<0.0001	↑
	DZ	*Hyphopichia*	<0.0001	↑
	DZ	*Lophotrichus*	<0.0001	↑
	DZ	*Pichia*	<0.0001	↑
	DZ	*Sebacina*	<0.001	↑
	DZ	*Chrysosporium*	<0.001	↑
	DZ	*Penicillium*	<0.001	↑
	DZ	*Clonostachys*	<0.01	↑
	DZ	*Rhodotorula*	<0.01	↑
	DZ	*Cortinarius*	<0.01	↑
	DZ	*Sporisorium*	<0.01	↑
	DZ	*Fusarium*	<0.01	↑
	DZ	*Amphinema*	<0.01	↑
	DZ	*Clavispora*	<0.01	↑
	DZ	*Verticillium*	<0.01	↑
	DZ	*Clavulina*	<0.05	↑
	DZ	*Ilyonectria*	<0.05	↑
	DZ	*Cordyceps*	<0.05	↑
	DZ	*Setophoma*	<0.05	↑
	DZ	*Acremonium*	<0.05	↑
	DZ	*Lecanicillium*	<0.05	↑
	DZ	*Pecoramyces*	<0.0001	↓
	DZ	*Caecomyces*	<0.0001	↓
	DZ	*Neocallimastix*	<0.0001	↓
	DZ	*Piromyces*	<0.0001	↓
	DZ	*Occultifur*	<0.0001	↓
	DZ	*Stephanonectria*	<0.001	↓
	DZ	*Papiliotrema*	<0.01	↓
	DZ	*Tuber*	<0.05	↓
	DZ	*Colletotrichum*	<0.05	↓
	DZ	*Saitozyma*	<0.05	↓
	EZ	*Xeromyces*	<0.0001	↑
	EZ	*Clavispora*	<0.0001	↑
	EZ	*Penicillium*	<0.0001	↑
	EZ	*Mortierella*	<0.0001	↑
	EZ	*Plectosphaerella*	<0.0001	↑
	EZ	*Gibberella*	<0.0001	↑
	EZ	*Russula*	<0.0001	↑
	EZ	*Wallemia*	<0.0001	↑
	EZ	*Sebacina*	<0.001	↑
	EZ	*Archaeorhizomyces*	<0.001	↑
	EZ	*Hirsutella*	<0.001	↑
	EZ	*Tomentella*	<0.001	↑
	EZ	*Aphanoascus*	<0.001	↑
	EZ	*Pseudallescheria*	<0.001	↑
	EZ	*Rhodotorula*	<0.01	↑
	EZ	*Verticillium*	<0.01	↑
	EZ	*Lophiostoma*	<0.01	↑
	EZ	*Filobasidium*	<0.05	↑
	EZ	*Aspergillus*	<0.05	↑
	EZ	*Thelonectria*	<0.05	↑
	EZ	*Ilyonectria*	<0.05	↑
	EZ	*Inocybe*	<0.05	↑
	EZ	*Neocallimastix*	<0.0001	↓
	EZ	*Occultifur*	<0.001	↓
	EZ	*Stephanonectria*	<0.001	↓
	EZ	*Phlebiopsis*	<0.01	↓
	EZ	*Setophoma*	<0.05	↓
	EZ	*Xenopolyscytalum*	<0.05	↓
	EZ	*Tuber*	<0.05	↓
	EZ	*Zopfiella*	<0.05	↓

## Data Availability

All raw sequence data were deposited in the NCBI Sequence Read Archive database under accession number PRJNA1008147.

## Supplementary Materials

The following supporting information can be downloaded at: https://www.mdpi.com/article/10.3390/life13112210/s1, Table S1: Nutritional ingredient information for the feeds used in the current study; Table S2: Detailed PCR information for this study; Table S3: Sequencing data generated in the current study; Table S4: Statistical analysis of the Alpha diversity index in the ST sheep groups; Table S5: Comparison of fungi microbiota function of enzyme abundances in ST sheep in different feeding groups; Figure S1. Body weights of the ST sheep in different groups. Significance is presented as * *p* < 0.05 and *** *p* < 0.001; data are presented as the mean ± SEM (*n* = 6); Figure S2. Comparing analyses of the Alpha diversity index in the ST sheep groups: (a) chao1; (b) faith_pd; (c) observed_features; (d) shannon_entropy; (e) Simpson. ns: non-significant; Figure S3. Pairwise ANOSIM analysis of Qiime2 in the five ST sheep groups.
