# Supplementary material for: Effect of Different Feeds on the Fungi Microbiome of Suffolk Crossed with Tibetan Sheep
Source: Life (Basel). 2023 Nov 14;13(11):2210. doi: 10.3390/life13112210 (PMC10672365; doi:10.3390/life13112210)
Supplement: Supplementary file 1 [file life-13-02210-s001.zip › life-2665624-supplementary.pdf]

Table S1. Nutritional ingredient information for the feeds used in the current study.

Feed Ingredient/Value	Concentrated Feed #1 (%)	Concentrated Feed #2 (%)	Concentrated Feed #3 (%)
Corn	35	45	50
Wheat bran	23.5	11.8	5.9
Soybean oil	0	2.5	3.8
Bean pulp	10	14.3	16.2
Alfalfa	20	17.4	16.3
Highland barley straw	10.5	8	6.8
Premix	0.5	0.5	0.5
Salt	0.5	0.5	0.5
Total	100	100	100
Water	6.50	6.50	7.90
Crude ash	13.30	13.20	12.40
Crude protein	9.42	8.62	8.87
Crude fat	35.00	33.00	40.00
Carbohydrate	35.78	38.68	30.83
Crude fiber	15.90	15.10	14.70
Acid detergent lignin	5.50	4.80	4.80
Neutral detergent fiber	46.50	41.40	44.20
Acid detergent fiber	25.60	21.50	21.50
Calcium	1.84	2.02	2.02
Phosphorus	0.27	0.24	0.24

Table S2. Detailed PCR information for this study.

	Reagents	Volume (μL)
Mixture	2.5 mM dNTPs (TransGen Biotech, China)	2
	5xFastPfu buffer (TransGen Biotech, China)	4
	FastPfu DNA Polymerase (TransGen Biotech, China)	0.4
	DNA template product	1
	ITS1 F	0.4
	ITS1 R	0.4
	Distilled water	11.8
	Total	20
Reaction	Steps	Time
27 cycles	Initial denaturation (95 °C)	3 min
	Denaturation (95 °C)	30 s
	Annealing (55 °C)	30 s
	Extension (72 °C)	30 s
	Extension (72 °C)	10 min

Table S3. Sequencing data generated in the current study.

Sample ID	Input	Filtered	Percentage of Input that Passed Filter	Denoised	Merged	Percentage of Input Merged	Non-Chimeric	Percentage of Non-Chimeric Input
AZ1	106,985	83,418	77.97	82,875	68,916	64.42	67,983	63.54
AZ2	127,675	107,567	84.25	106,744	89,827	70.36	89,825	70.35
AZ3	73,479	61,573	83.80	61,042	47,863	65.14	47,826	65.09
AZ4	133,556	112,144	83.97	111,183	82,943	62.10	82,891	62.06
AZ5	132,711	115,063	86.70	112,384	107,648	81.11	106,536	80.28

BZ1	114,309	96,159	84.12	94,817	76,879	67.26	76,512	66.93
BZ2	135,563	113,999	84.09	112,481	100,328	74.01	99,102	73.10
BZ3	105,663	89,654	84.85	88,280	76,650	72.54	76,401	72.31
BZ4	125,450	104,920	83.63	103,398	88,642	70.66	88,493	70.54
BZ5	137,999	118,030	85.53	117,097	107,519	77.91	106,397	77.10
CZ1	75,827	64,179	84.64	63,408	61,571	81.20	61,103	80.58
CZ2	83,885	71,770	85.56	70,870	68,007	81.07	67,007	79.88
CZ3	50,826	41,264	81.19	40,790	37,454	73.69	37,318	73.42
CZ4	82,031	65,256	79.55	64,465	63,167	77.00	62,851	76.62
CZ5	74,058	61,685	83.29	60,875	56,893	76.82	56,723	76.59
DZ1	160,885	142,520	88.59	141,197	130,430	81.07	128,799	80.06
DZ2	84,462	74,725	88.47	73,660	72,171	85.45	71,818	85.03
DZ3	144,371	122,026	84.52	120,533	117,150	81.15	109,909	76.13
DZ4	158,962	131,807	82.92	130,518	125,890	79.20	122,955	77.35
DZ5	101,303	82,166	81.11	81,079	74,626	73.67	72,686	71.75
EZ1	161,119	137,087	85.08	134,215	129,788	80.55	127,008	78.83
EZ2	160,324	123,694	77.15	122,910	120,506	75.16	119,838	74.75
EZ3	134,226	115,604	86.13	114,534	106,322	79.21	104,428	77.80
EZ4	62,310	53,843	86.41	52,937	51,369	82.44	51,047	81.92
EZ5	53,482	43,086	80.56	41,941	40,362	75.47	38,499	71.98

Table S4. Statistical analysis of the Alpha diversity index in the ST sheep groups.

Sample	chao1	faith_pd	observed_features	shannon_entropy	Simpson
AZ1	135	30.30201837	135	4.184765421	0.903904095
AZ2	198	39.94587916	198	4.545931900	0.932746861
AZ3	105	21.13236541	105	4.261718745	0.925120176
AZ4	196	48.12695369	196	4.525674383	0.936532637
AZ5	343	78.51589695	343	3.950515182	0.755557375
BZ1	314	60.95170897	314	5.002819171	0.941507561
BZ2	255	61.5090516	255	4.564360126	0.914389386
BZ3	196	48.77441271	196	4.366933470	0.908789477
BZ4	190	43.02040271	190	3.563486979	0.802307536
BZ5	210	45.13994307	210	4.006353752	0.858627985
CZ1	277	48.81506241	277	5.561021794	0.931097328
CZ2	384	47.51557581	384	7.158246222	0.98635995
CZ3	154	29.86679489	154	4.048319309	0.876663685
CZ4	246	47.90667354	246	5.313414881	0.943519086
CZ5	207	47.42970281	207	4.996391213	0.930689045
DZ1	168	45.74432897	168	1.882440470	0.452769305
DZ2	189	50.74399507	189	1.898805092	0.433119129
DZ3	432	61.87250318	432	7.173194506	0.988832848
DZ4	263	47.58961621	263	2.966428793	0.610751670
DZ5	105	27.80599644	105	5.102872495	0.962086167
EZ1	495	84.06189545	495	4.643088381	0.751595398
EZ2	210	33.28028689	210	5.712930128	0.957193016
EZ3	152	35.77818851	152	2.834970958	0.668057691
EZ4	294	45.17737199	294	5.407603476	0.932955730
EZ5	388	60.16791782	388	6.630918580	0.978107556

Table S5. Comparison of fungi microbiota function of enzyme abundances in ST sheep in different feeding groups.

Feature	Group	Mean	SE	Duncan significance
O55528, L_SENDO	AZ	0.868785	0.101617	a
O55528, L_SENDO	BZ	0.960813	0.033028	a
O55528, L_SENDO	CZ	-0.36937	0.171259	b
O55528, L_SENDO	DZ	-0.78839	0.112974	b
O55528, L_SENDO	EZ	-0.67183	0.205244	b
Q06996, L_SENDF	AZ	-0.90319	0.140407	b
Q06996, L_SENDF	BZ	-0.87225	0.17922	b
Q06996, L_SENDF	CZ	0.601327	0.12491	a
Q06996, L_SENDF	DZ	0.692534	0.071604	a
Q06996, L_SENDF	EZ	0.48158	0.186954	a
(+)-neomenthol dehydrogenase	AZ	-0.9386	0.116555	b
(+)-neomenthol dehydrogenase	BZ	-0.93074	0.165107	b
(+)-neomenthol dehydrogenase	CZ	0.613474	0.076258	a
(+)-neomenthol dehydrogenase	DZ	0.493999	0.06809	a
(+)-neomenthol dehydrogenase	EZ	0.761863	0.203224	a
(decarboxylating)	AZ	-0.82661	0.095036	b
(decarboxylating)	BZ	-0.81694	0.069423	b
(decarboxylating)	CZ	0.359886	0.170183	a
(decarboxylating)	DZ	0.499311	0.108662	a
(decarboxylating)	EZ	0.784344	0.261045	a
(R,R)-butanediol dehydrogenase	AZ	-0.94789	0.172882	b
(R,R)-butanediol dehydrogenase	BZ	-0.80561	0.10263	b
(R,R)-butanediol dehydrogenase	CZ	0.480497	0.12514	a
(R,R)-butanediol dehydrogenase	DZ	0.802509	0.044393	a
(R,R)-butanediol dehydrogenase	EZ	0.470498	0.223068	a
(S)-2-haloacid dehalogenase	AZ	-0.90263	0.091082	b
(S)-2-haloacid dehalogenase	BZ	-1.07374	0.099481	b
(S)-2-haloacid dehalogenase	CZ	0.707393	0.113941	a
(S)-2-haloacid dehalogenase	DZ	0.674483	0.092258	a
(S)-2-haloacid dehalogenase	EZ	0.594485	0.191034	a
[Acyl-carrier-protein] S-malonyltransferase	AZ	0.798708	0.081445	a
[Acyl-carrier-protein] S-malonyltransferase	BZ	0.870202	0.026205	a
[Acyl-carrier-protein] S-malonyltransferase	CZ	-0.62356	0.279293	b
[Acyl-carrier-protein] S-malonyltransferase	DZ	-0.57455	0.100397	b
[Acyl-carrier-protein] S-malonyltransferase	EZ	-0.47079	0.166754	b
[Formate-C-acetyltransferase]-activating enzyme	AZ	-0.92001	0.089257	b
[Formate-C-acetyltransferase]-activating enzyme	BZ	-1.07806	0.098461	b
[Formate-C-acetyltransferase]-activating enzyme	CZ	0.691931	0.100971	a
[Formate-C-acetyltransferase]-activating enzyme	DZ	0.653679	0.079761	a
[Formate-C-acetyltransferase]-activating enzyme	EZ	0.652455	0.196882	a

[Pyruvate dehydrogenase (acetyl-transferring)]-phosphatase	AZ	-0.94044	0.132781	b
[Pyruvate dehydrogenase (acetyl-transferring)]-phosphatase	BZ	-0.99541	0.114632	b
[Pyruvate dehydrogenase (acetyl-transferring)]-phosphatase	CZ	0.721605	0.123914	a
[Pyruvate dehydrogenase (acetyl-transferring)]-phosphatase	DZ	0.745665	0.054465	a
[Pyruvate dehydrogenase (acetyl-transferring)]-phosphatase	EZ	0.468576	0.179804	a
[Ribulose-bisphosphate carboxylase]-lysine N-methyltransferase	AZ	-0.99421	0.147532	b
[Ribulose-bisphosphate carboxylase]-lysine N-methyltransferase	BZ	-0.86166	0.077661	b
[Ribulose-bisphosphate carboxylase]-lysine N-methyltransferase	CZ	0.562258	0.111959	a
[Ribulose-bisphosphate carboxylase]-lysine N-methyltransferase	DZ	0.958879	0.040416	a
[Ribulose-bisphosphate carboxylase]-lysine N-methyltransferase	EZ	0.334723	0.210021	a
1-acylglycerol-3-phosphate O-acyltransferase	AZ	0.849033	0.113254	a
1-acylglycerol-3-phosphate O-acyltransferase	BZ	0.952843	0.036724	a
1-acylglycerol-3-phosphate O-acyltransferase	CZ	-0.4421	0.182474	b
1-acylglycerol-3-phosphate O-acyltransferase	DZ	-0.80786	0.128023	b
1-acylglycerol-3-phosphate O-acyltransferase	EZ	-0.55191	0.192523	b
1-alkyl-2-acetylglycerophosphocholine esterase	AZ	0.882539	0.074319	a
1-alkyl-2-acetylglycerophosphocholine esterase	BZ	0.964239	0.021636	a
1-alkyl-2-acetylglycerophosphocholine esterase	CZ	-0.40076	0.173143	b
1-alkyl-2-acetylglycerophosphocholine esterase	DZ	-0.7695	0.137679	b
1-alkyl-2-acetylglycerophosphocholine esterase	EZ	-0.67652	0.197793	b
1-aminocyclopropane-1-carboxylate synthase	AZ	0.760842	0.104223	ab
1-aminocyclopropane-1-carboxylate synthase	BZ	0.932409	0.036392	a
1-aminocyclopropane-1-carboxylate synthase	CZ	-0.0496	0.122862	bc
1-aminocyclopropane-1-carboxylate synthase	DZ	-1.08175	0.183892	d
1-aminocyclopropane-1-carboxylate synthase	EZ	-0.56191	0.17908	cd
1-phosphatidylinositol-4-phosphate 5-kinase	AZ	0.896964	0.068937	a
1-phosphatidylinositol-4-phosphate 5-kinase	BZ	0.961304	0.020695	a
1-phosphatidylinositol-4-phosphate 5-kinase	CZ	-0.36173	0.173177	b
1-phosphatidylinositol-4-phosphate 5-kinase	DZ	-0.7467	0.117534	b
1-phosphatidylinositol-4-phosphate 5-kinase	EZ	-0.74983	0.205059	b
1,3-beta-glucan synthase	AZ	-0.94024	0.160129	b
1,3-beta-glucan synthase	BZ	-0.88153	0.105356	b
1,3-beta-glucan synthase	CZ	0.506743	0.09901	a
1,3-beta-glucan synthase	DZ	0.847554	0.050612	a
1,3-beta-glucan synthase	EZ	0.467474	0.219439	a
1,4-alpha-glucan branching enzyme	AZ	0.866802	0.103111	a
1,4-alpha-glucan branching enzyme	BZ	0.962972	0.033353	a
1,4-alpha-glucan branching enzyme	CZ	-0.38555	0.173323	b
1,4-alpha-glucan branching enzyme	DZ	-0.76463	0.109365	b
1,4-alpha-glucan branching enzyme	EZ	-0.67959	0.206309	b
15-hydroxyprostaglandin dehydrogenase (NAD(+))	AZ	-0.80474	0.142095	b
15-hydroxyprostaglandin dehydrogenase (NAD(+))	BZ	-0.94476	0.058246	b
15-hydroxyprostaglandin dehydrogenase (NAD(+))	CZ	0.514036	0.147743	a
15-hydroxyprostaglandin dehydrogenase (NAD(+))	DZ	0.562261	0.136494	a

15-hydroxyprostaglandin dehydrogenase (NAD(+))	EZ	0.673202	0.217418	a
2-acylglycerol O-acyltransferase	AZ	-0.89015	0.116878	b
2-acylglycerol O-acyltransferase	BZ	-0.97146	0.155143	b
2-acylglycerol O-acyltransferase	CZ	0.64138	0.1246	a
2-acylglycerol O-acyltransferase	DZ	0.692759	0.082785	a
2-acylglycerol O-acyltransferase	EZ	0.527478	0.186038	a
2-amino-4-hydroxy-6-hydroxymethyldihydropteridine diphosphokinase	AZ	-0.924	0.136053	c
2-amino-4-hydroxy-6-hydroxymethyldihydropteridine diphosphokinase	BZ	-0.45548	0.180335	bc
2-amino-4-hydroxy-6-hydroxymethyldihydropteridine diphosphokinase	CZ	0.238126	0.184639	ab
2-amino-4-hydroxy-6-hydroxymethyldihydropteridine diphosphokinase	DZ	1.014466	0.052674	a
2-amino-4-hydroxy-6-hydroxymethyldihydropteridine diphosphokinase	EZ	0.126879	0.209629	abc
2-aminomuconate deaminase	AZ	-0.90048	0.095242	b
2-aminomuconate deaminase	BZ	-1.04301	0.141592	b
2-aminomuconate deaminase	CZ	0.664868	0.10057	a
2-aminomuconate deaminase	DZ	0.611296	0.07722	a
2-aminomuconate deaminase	EZ	0.667329	0.191867	a
2-deoxy-D-gluconate 3-dehydrogenase	AZ	-0.9385	0.158605	b
2-deoxy-D-gluconate 3-dehydrogenase	BZ	-0.87207	0.100589	b
2-deoxy-D-gluconate 3-dehydrogenase	CZ	0.578711	0.11192	a
2-deoxy-D-gluconate 3-dehydrogenase	DZ	0.773373	0.044777	a
2-deoxy-D-gluconate 3-dehydrogenase	EZ	0.458483	0.225765	a
2-methylacyl-CoA dehydrogenase	AZ	-0.87787	0.103406	b
2-methylacyl-CoA dehydrogenase	BZ	-1.00487	0.139632	b
2-methylacyl-CoA dehydrogenase	CZ	0.685816	0.133192	a
2-methylacyl-CoA dehydrogenase	DZ	0.708469	0.092184	a
2-methylacyl-CoA dehydrogenase	EZ	0.488456	0.184412	a
2-methylcitrate dehydratase	AZ	-0.90846	0.153034	b
2-methylcitrate dehydratase	BZ	-0.93471	0.097663	b
2-methylcitrate dehydratase	CZ	0.578496	0.104604	a
2-methylcitrate dehydratase	DZ	0.810674	0.056247	a
2-methylcitrate dehydratase	EZ	0.453998	0.219294	a
2,3-dihydro-2,3-dihydroxybenzoate dehydrogenase	AZ	-1.01316	0.179962	c
2,3-dihydro-2,3-dihydroxybenzoate dehydrogenase	BZ	-0.64245	0.117927	bc
2,3-dihydro-2,3-dihydroxybenzoate dehydrogenase	CZ	0.476082	0.116825	a
2,3-dihydro-2,3-dihydroxybenzoate dehydrogenase	DZ	0.815864	0.05479	a
2,3-dihydro-2,3-dihydroxybenzoate dehydrogenase	EZ	0.363659	0.232097	ab
2,4-dienoyl-CoA reductase (NADPH)	AZ	0.845961	0.10174	a
2,4-dienoyl-CoA reductase (NADPH)	BZ	0.976177	0.035923	a
2,4-dienoyl-CoA reductase (NADPH)	CZ	-0.4716	0.17971	b
2,4-dienoyl-CoA reductase (NADPH)	DZ	-0.76476	0.132542	b
2,4-dienoyl-CoA reductase (NADPH)	EZ	-0.58577	0.193769	b
2.7.11.30 and 2.7.12.1	AZ	0.852737	0.096074	a
2.7.11.30 and 2.7.12.1	BZ	0.956348	0.031586	a
2.7.11.30 and 2.7.12.1	CZ	-0.5068	0.177683	b

2.7.11.30 and 2.7.12.1	DZ	-0.63359	0.136888	b
2.7.11.30 and 2.7.12.1	EZ	-0.6687	0.20519	b
2'-hydroxyisoflavone reductase	AZ	-0.87367	0.121866	b
2'-hydroxyisoflavone reductase	BZ	-0.49316	0.20247	b
2'-hydroxyisoflavone reductase	CZ	0.168614	0.188644	ab
2'-hydroxyisoflavone reductase	DZ	0.918442	0.070813	a
2'-hydroxyisoflavone reductase	EZ	0.279781	0.213813	ab
3-carboxy-cis,cis-muconate cycloisomerase	AZ	-0.5086	0.176774	ab
3-carboxy-cis,cis-muconate cycloisomerase	BZ	-1.0104	0.153114	b
3-carboxy-cis,cis-muconate cycloisomerase	CZ	0.464457	0.161095	a
3-carboxy-cis,cis-muconate cycloisomerase	DZ	0.579774	0.110724	a
3-carboxy-cis,cis-muconate cycloisomerase	EZ	0.474772	0.210363	a
3-dehydroquinate dehydratase	AZ	-0.95762	0.161203	b
3-dehydroquinate dehydratase	BZ	-0.81603	0.100756	b
3-dehydroquinate dehydratase	CZ	0.552326	0.101849	a
3-dehydroquinate dehydratase	DZ	0.815465	0.058521	a
3-dehydroquinate dehydratase	EZ	0.405866	0.232761	a
3-dehydrosphinganine reductase	AZ	0.725484	0.084922	a
3-dehydrosphinganine reductase	BZ	0.856233	0.019798	a
3-dehydrosphinganine reductase	CZ	0.110176	0.086461	ab
3-dehydrosphinganine reductase	DZ	-0.86398	0.199888	b
3-dehydrosphinganine reductase	EZ	-0.82792	0.223138	b
3-demethylubiquinol 3-O-methyltransferase	AZ	-0.89651	0.15924	b
3-demethylubiquinol 3-O-methyltransferase	BZ	-0.88362	0.099869	b
3-demethylubiquinol 3-O-methyltransferase	CZ	0.483009	0.18628	a
3-demethylubiquinol 3-O-methyltransferase	DZ	0.84042	0.075369	a
3-demethylubiquinol 3-O-methyltransferase	EZ	0.4567	0.166293	a
3-hydroxyacyl-CoA dehydrogenase	AZ	0.546019	0.049276	a
3-hydroxyacyl-CoA dehydrogenase	BZ	0.20976	0.06328	a
3-hydroxyacyl-CoA dehydrogenase	CZ	0.345269	0.222887	a
3-hydroxyacyl-CoA dehydrogenase	DZ	-1.18908	0.278514	b
3-hydroxyacyl-CoA dehydrogenase	EZ	0.088028	0.110314	a
3-hydroxyanthranilate 3,4-dioxygenase	AZ	0.742527	0.100591	a
3-hydroxyanthranilate 3,4-dioxygenase	BZ	0.984221	0.033067	a
3-hydroxyanthranilate 3,4-dioxygenase	CZ	-0.33364	0.171146	b
3-hydroxyanthranilate 3,4-dioxygenase	DZ	-0.66276	0.171641	b
3-hydroxyanthranilate 3,4-dioxygenase	EZ	-0.73035	0.199589	b
3-hydroxyisobutyrate dehydrogenase	AZ	-0.77683	0.150178	b
3-hydroxyisobutyrate dehydrogenase	BZ	-0.91205	0.059318	b
3-hydroxyisobutyrate dehydrogenase	CZ	0.486076	0.13607	a
3-hydroxyisobutyrate dehydrogenase	DZ	0.401465	0.126581	a
3-hydroxyisobutyrate dehydrogenase	EZ	0.80134	0.235893	a
3-hydroxyisobutyryl-CoA hydrolase	AZ	-0.90841	0.123829	b
3-hydroxyisobutyryl-CoA hydrolase	BZ	-0.94353	0.150456	b

3-hydroxyisobutyryl-CoA hydrolase	CZ	0.638615	0.12396	a
3-hydroxyisobutyryl-CoA hydrolase	DZ	0.709639	0.07942	a
3-hydroxyisobutyryl-CoA hydrolase	EZ	0.50368	0.190599	a
3-isopropylmalate dehydratase	AZ	-0.96988	0.158385	b
3-isopropylmalate dehydratase	BZ	-0.83856	0.099886	b
3-isopropylmalate dehydratase	CZ	0.552833	0.111198	a
3-isopropylmalate dehydratase	DZ	0.826346	0.042996	a
3-isopropylmalate dehydratase	EZ	0.42926	0.22337	a
3-isopropylmalate dehydrogenase	AZ	-0.89443	0.164687	b
3-isopropylmalate dehydrogenase	BZ	-0.89409	0.106388	b
3-isopropylmalate dehydrogenase	CZ	0.589939	0.11853	a
3-isopropylmalate dehydrogenase	DZ	0.773226	0.050438	a
3-isopropylmalate dehydrogenase	EZ	0.425352	0.22058	a
3-mercaptopyruvate sulfurtransferase	AZ	-1.02877	0.15774	b
3-mercaptopyruvate sulfurtransferase	BZ	-0.22402	0.205929	ab
3-mercaptopyruvate sulfurtransferase	CZ	0.315487	0.184408	a
3-mercaptopyruvate sulfurtransferase	DZ	0.814878	0.11712	a
3-mercaptopyruvate sulfurtransferase	EZ	0.12243	0.175021	ab
3-methyl-2-oxobutanoate dehydrogenase (2-methylpropanoyl-transferring)	AZ	-0.67831	0.13214	bc
3-methyl-2-oxobutanoate dehydrogenase (2-methylpropanoyl-transferring)	BZ	-0.90648	0.031547	c
3-methyl-2-oxobutanoate dehydrogenase (2-methylpropanoyl-transferring)	CZ	0.287492	0.22581	ab
3-methyl-2-oxobutanoate dehydrogenase (2-methylpropanoyl-transferring)	DZ	1.040566	0.126578	a
3-methyl-2-oxobutanoate dehydrogenase (2-methylpropanoyl-transferring)	EZ	0.256738	0.169922	ab
3-methyl-2-oxobutanoate hydroxymethyltransferase	AZ	0.801537	0.125182	a
3-methyl-2-oxobutanoate hydroxymethyltransferase	BZ	0.911049	0.03934	a
3-methyl-2-oxobutanoate hydroxymethyltransferase	CZ	-0.25136	0.195845	b
3-methyl-2-oxobutanoate hydroxymethyltransferase	DZ	-0.80016	0.098401	b
3-methyl-2-oxobutanoate hydroxymethyltransferase	EZ	-0.66106	0.209995	b
3-oxo-5-alpha-steroid 4-dehydrogenase (NADP(+))	AZ	-0.89015	0.116878	b
3-oxo-5-alpha-steroid 4-dehydrogenase (NADP(+))	BZ	-0.97146	0.155143	b
3-oxo-5-alpha-steroid 4-dehydrogenase (NADP(+))	CZ	0.64138	0.1246	a
3-oxo-5-alpha-steroid 4-dehydrogenase (NADP(+))	DZ	0.692759	0.082785	a
3-oxo-5-alpha-steroid 4-dehydrogenase (NADP(+))	EZ	0.527478	0.186038	a
3-oxoacid CoA-transferase	AZ	-0.92755	0.162304	b
3-oxoacid CoA-transferase	BZ	-0.90297	0.094453	b
3-oxoacid CoA-transferase	CZ	0.600575	0.105088	a
3-oxoacid CoA-transferase	DZ	0.808763	0.062495	a
3-oxoacid CoA-transferase	EZ	0.421181	0.21539	a
3-oxoacyl-[acyl-carrier-protein] reductase	AZ	-0.87808	0.148988	b
3-oxoacyl-[acyl-carrier-protein] reductase	BZ	-0.96961	0.064726	b
3-oxoacyl-[acyl-carrier-protein] reductase	CZ	0.482323	0.127316	a
3-oxoacyl-[acyl-carrier-protein] reductase	DZ	0.798095	0.082617	a
3-oxoacyl-[acyl-carrier-protein] reductase	EZ	0.567271	0.213192	a
3-phytase	AZ	-0.89582	0.098159	b

3-phytase	BZ	-1.04781	0.110129	b
3-phytase	CZ	0.689888	0.123216	a
3-phytase	DZ	0.705726	0.087445	a
3-phytase	EZ	0.548011	0.191501	a
3',5'-cyclic-nucleotide phosphodiesterase	AZ	0.884821	0.064693	a
3',5'-cyclic-nucleotide phosphodiesterase	BZ	0.953264	0.018811	a
3',5'-cyclic-nucleotide phosphodiesterase	CZ	-0.33093	0.181172	b
3',5'-cyclic-nucleotide phosphodiesterase	DZ	-0.77218	0.124458	b
3',5'-cyclic-nucleotide phosphodiesterase	EZ	-0.73497	0.200671	b
4-aminobutyrate--2-oxoglutarate transaminase	AZ	-0.92832	0.161736	b
4-aminobutyrate--2-oxoglutarate transaminase	BZ	-0.87581	0.101735	b
4-aminobutyrate--2-oxoglutarate transaminase	CZ	0.544279	0.117331	a
4-aminobutyrate--2-oxoglutarate transaminase	DZ	0.804938	0.051326	a
4-aminobutyrate--2-oxoglutarate transaminase	EZ	0.454909	0.219305	a
4-carboxymuconolactone decarboxylase	AZ	-0.72824	0.069855	b
4-carboxymuconolactone decarboxylase	BZ	-0.77055	0.071878	b
4-carboxymuconolactone decarboxylase	CZ	0.617903	0.033573	a
4-carboxymuconolactone decarboxylase	DZ	-0.03075	0.280906	ab
4-carboxymuconolactone decarboxylase	EZ	0.911644	0.181719	a
4-coumarate--CoA ligase	AZ	-0.80479	0.136435	b
4-coumarate--CoA ligase	BZ	-0.94622	0.046719	b
4-coumarate--CoA ligase	CZ	0.403603	0.143484	a
4-coumarate--CoA ligase	DZ	0.594699	0.129728	a
4-coumarate--CoA ligase	EZ	0.7527	0.224676	a
4-hydroxybenzoate polyprenyltransferase	AZ	-0.91656	0.118197	b
4-hydroxybenzoate polyprenyltransferase	BZ	-0.97067	0.144817	b
4-hydroxybenzoate polyprenyltransferase	CZ	0.628638	0.111633	a
4-hydroxybenzoate polyprenyltransferase	DZ	0.673986	0.077173	a
4-hydroxybenzoate polyprenyltransferase	EZ	0.584604	0.194848	a
4-nitrophenylphosphatase	AZ	0.878353	0.085004	a
4-nitrophenylphosphatase	BZ	0.968281	0.024621	a
4-nitrophenylphosphatase	CZ	-0.36492	0.165003	b
4-nitrophenylphosphatase	DZ	-0.76481	0.128795	b
4-nitrophenylphosphatase	EZ	-0.7169	0.203218	b
4a-hydroxytetrahydrobiopterin dehydratase	AZ	0.817588	0.075649	a
4a-hydroxytetrahydrobiopterin dehydratase	BZ	0.869721	0.025161	a
4a-hydroxytetrahydrobiopterin dehydratase	CZ	-0.44106	0.203456	b
4a-hydroxytetrahydrobiopterin dehydratase	DZ	-0.99052	0.188157	b
4a-hydroxytetrahydrobiopterin dehydratase	EZ	-0.25573	0.153463	b
5-amino-6-(5-phosphoribosylamino)uracil reductase	AZ	0.854312	0.061466	a
5-amino-6-(5-phosphoribosylamino)uracil reductase	BZ	0.930703	0.015196	a
5-amino-6-(5-phosphoribosylamino)uracil reductase	CZ	-0.48146	0.210348	b
5-amino-6-(5-phosphoribosylamino)uracil reductase	DZ	-0.69709	0.154286	b
5-amino-6-(5-phosphoribosylamino)uracil reductase	EZ	-0.60647	0.184014	b

5-aminolevulinate synthase	AZ	0.866659	0.102881	a
5-aminolevulinate synthase	BZ	0.962763	0.033284	a
5-aminolevulinate synthase	CZ	-0.38295	0.172962	b
5-aminolevulinate synthase	DZ	-0.76987	0.110636	b
5-aminolevulinate synthase	EZ	-0.6766	0.205898	b
5-carboxymethyl-2-hydroxymuconate Delta-isomerase	AZ	-0.86771	0.069463	b
5-carboxymethyl-2-hydroxymuconate Delta-isomerase	BZ	-0.85437	0.037858	b
5-carboxymethyl-2-hydroxymuconate Delta-isomerase	CZ	0.704846	0.237206	a
5-carboxymethyl-2-hydroxymuconate Delta-isomerase	DZ	0.562696	0.145386	a
5-carboxymethyl-2-hydroxymuconate Delta-isomerase	EZ	0.454537	0.176614	a
5-formyltetrahydrofolate cyclo-ligase	AZ	0.866798	0.103114	a
5-formyltetrahydrofolate cyclo-ligase	BZ	0.96297	0.033354	a
5-formyltetrahydrofolate cyclo-ligase	CZ	-0.38558	0.173327	b
5-formyltetrahydrofolate cyclo-ligase	DZ	-0.76467	0.109368	b
5-formyltetrahydrofolate cyclo-ligase	EZ	-0.67951	0.206306	b
5-oxoprolinase (ATP-hydrolyzing)	AZ	-0.84861	0.099351	b
5-oxoprolinase (ATP-hydrolyzing)	BZ	-1.00458	0.030789	b
5-oxoprolinase (ATP-hydrolyzing)	CZ	0.459431	0.131482	a
5-oxoprolinase (ATP-hydrolyzing)	DZ	0.712783	0.140114	a
5-oxoprolinase (ATP-hydrolyzing)	EZ	0.680976	0.215992	a
5'-nucleotidase	AZ	0.84852	0.078195	a
5'-nucleotidase	BZ	0.918683	0.031917	a
5'-nucleotidase	CZ	-0.36782	0.184946	b
5'-nucleotidase	DZ	-0.54345	0.122745	b
5'-nucleotidase	EZ	-0.85594	0.217336	b
6-phosphofructo-2-kinase	AZ	0.871831	0.07449	a
6-phosphofructo-2-kinase	BZ	0.957877	0.019346	a
6-phosphofructo-2-kinase	CZ	-0.45365	0.213625	b
6-phosphofructo-2-kinase	DZ	-0.75505	0.104492	b
6-phosphofructo-2-kinase	EZ	-0.621	0.188585	b
6-phosphofructokinase	AZ	0.849964	0.106549	a
6-phosphofructokinase	BZ	0.982286	0.033704	a
6-phosphofructokinase	CZ	-0.44347	0.178775	b
6-phosphofructokinase	DZ	-0.71598	0.101052	b
6-phosphofructokinase	EZ	-0.6728	0.207064	b
6-phosphogluconolactonase	AZ	0.815887	0.106006	a
6-phosphogluconolactonase	BZ	0.963728	0.036899	a
6-phosphogluconolactonase	CZ	-0.34295	0.190561	b
6-phosphogluconolactonase	DZ	-0.8691	0.110658	b
6-phosphogluconolactonase	EZ	-0.56756	0.198409	b
7-dehydrocholesterol reductase	AZ	0.76398	0.078966	a
7-dehydrocholesterol reductase	BZ	0.807358	0.029013	a
7-dehydrocholesterol reductase	CZ	-0.3341	0.216877	b
7-dehydrocholesterol reductase	DZ	-0.94692	0.212424	b

7-dehydrocholesterol reductase	EZ	-0.29032	0.156077	b
8-amino-7-oxononanoate synthase	AZ	0.724009	0.075366	a
8-amino-7-oxononanoate synthase	BZ	0.798601	0.059547	a
8-amino-7-oxononanoate synthase	CZ	0.082487	0.062249	ab
8-amino-7-oxononanoate synthase	DZ	-0.55836	0.127426	bc
8-amino-7-oxononanoate synthase	EZ	-1.04673	0.286099	c
Acetate kinase	AZ	-0.92242	0.157569	b
Acetate kinase	BZ	-0.89903	0.097209	b
Acetate kinase	CZ	0.557964	0.102889	a
Acetate kinase	DZ	0.843406	0.051023	a
Acetate kinase	EZ	0.420081	0.222757	a
Acetate--CoA ligase	AZ	-0.11601	0.038818	a
Acetate--CoA ligase	BZ	0.314628	0.128264	a
Acetate--CoA ligase	CZ	0.638771	0.201298	a
Acetate--CoA ligase	DZ	0.378298	0.191059	a
Acetate--CoA ligase	EZ	-1.21568	0.19479	b
Acetoacetate--CoA ligase	AZ	-0.69165	0.166661	bc
Acetoacetate--CoA ligase	BZ	-0.94618	0.047433	c
Acetoacetate--CoA ligase	CZ	0.187057	0.102806	ab
Acetoacetate--CoA ligase	DZ	0.816573	0.180614	a
Acetoacetate--CoA ligase	EZ	0.634196	0.20713	a
Acetyl-CoA C-acetyltransferase	AZ	0.905191	0.087418	a
Acetyl-CoA C-acetyltransferase	BZ	0.959291	0.032546	a
Acetyl-CoA C-acetyltransferase	CZ	-0.44501	0.191458	b
Acetyl-CoA C-acetyltransferase	DZ	-0.87239	0.078018	b
Acetyl-CoA C-acetyltransferase	EZ	-0.54708	0.19661	b
Acetyl-CoA hydrolase	AZ	0.791576	0.081117	a
Acetyl-CoA hydrolase	BZ	0.8819	0.020402	a
Acetyl-CoA hydrolase	CZ	-0.631	0.278897	b
Acetyl-CoA hydrolase	DZ	-0.57816	0.100154	b
Acetyl-CoA hydrolase	EZ	-0.46432	0.166047	b
Acetylcholinesterase	AZ	-0.86188	0.126618	b
Acetylcholinesterase	BZ	-0.90906	0.059623	b
Acetylcholinesterase	CZ	0.264927	0.156742	a
Acetylcholinesterase	DZ	0.934334	0.106546	a
Acetylcholinesterase	EZ	0.571672	0.206236	a
Acetylornithine deacetylase	AZ	-0.96443	0.15407	b
Acetylornithine deacetylase	BZ	-0.85747	0.094761	b
Acetylornithine deacetylase	CZ	0.554523	0.103517	a
Acetylornithine deacetylase	DZ	0.77179	0.064101	a
Acetylornithine deacetylase	EZ	0.495589	0.227488	a
Acetylornithine transaminase	AZ	0.918532	0.100256	a
Acetylornithine transaminase	BZ	0.894741	0.063502	a
Acetylornithine transaminase	CZ	-0.43012	0.206591	b

Acetylornithine transaminase	DZ	-0.82256	0.056604	b
Acetylornithine transaminase	EZ	-0.56059	0.197549	b
Acetylserotonin O-methyltransferase	AZ	-0.85427	0.130181	b
Acetylserotonin O-methyltransferase	BZ	-0.44374	0.213826	b
Acetylserotonin O-methyltransferase	CZ	0.210786	0.2007	ab
Acetylserotonin O-methyltransferase	DZ	0.957759	0.061869	a
Acetylserotonin O-methyltransferase	EZ	0.129469	0.194843	ab
Aconitate decarboxylase	AZ	-0.86325	0.081759	b
Aconitate decarboxylase	BZ	-1.18338	0.063704	b
Aconitate decarboxylase	CZ	0.715246	0.102477	a
Aconitate decarboxylase	DZ	0.639747	0.089177	a
Aconitate decarboxylase	EZ	0.691636	0.183203	a
Aconitate hydratase	AZ	0.880259	0.101058	a
Aconitate hydratase	BZ	0.94252	0.035349	a
Aconitate hydratase	CZ	-0.54797	0.200071	b
Aconitate hydratase	DZ	-0.66979	0.097226	b
Aconitate hydratase	EZ	-0.60502	0.200373	b
Acyl-CoA oxidase	AZ	0.85663	0.071288	a
Acyl-CoA oxidase	BZ	0.927819	0.022358	a
Acyl-CoA oxidase	CZ	-0.26787	0.147	b
Acyl-CoA oxidase	DZ	-0.8677	0.186179	b
Acyl-CoA oxidase	EZ	-0.64888	0.190075	b
Acylaminoacyl-peptidase	AZ	0.829307	0.076249	ab
Acylaminoacyl-peptidase	BZ	0.886384	0.027962	a
Acylaminoacyl-peptidase	CZ	-0.07713	0.116808	bc
Acylaminoacyl-peptidase	DZ	-0.98648	0.200399	c
Acylaminoacyl-peptidase	EZ	-0.65208	0.188981	c
Acylglycerol lipase	AZ	0.87498	0.079928	a
Acylglycerol lipase	BZ	0.947376	0.024396	a
Acylglycerol lipase	CZ	-0.30992	0.161839	b
Acylglycerol lipase	DZ	-0.8419	0.147815	b
Acylglycerol lipase	EZ	-0.67054	0.197729	b
Acylglycerone-phosphate reductase	AZ	-0.93268	0.161856	b
Acylglycerone-phosphate reductase	BZ	-0.87443	0.096439	b
Acylglycerone-phosphate reductase	CZ	0.536719	0.108635	a
Acylglycerone-phosphate reductase	DZ	0.82935	0.056622	a
Acylglycerone-phosphate reductase	EZ	0.441046	0.221918	a
Acylphosphatase	AZ	-0.939	0.167387	b
Acylphosphatase	BZ	-0.8571	0.097372	b
Acylphosphatase	CZ	0.517209	0.093246	a
Acylphosphatase	DZ	0.808125	0.08202	a
Acylphosphatase	EZ	0.470758	0.221704	a
Acylpyruvate hydrolase	AZ	-0.89546	0.084654	b
Acylpyruvate hydrolase	BZ	-1.09751	0.102114	b

Acylpyruvate hydrolase	CZ	0.714532	0.108073	a
Acylpyruvate hydrolase	DZ	0.655814	0.090976	a
Acylpyruvate hydrolase	EZ	0.622626	0.188986	a
Adenine phosphoribosyltransferase	AZ	0.850296	0.112653	a
Adenine phosphoribosyltransferase	BZ	0.954259	0.036853	a
Adenine phosphoribosyltransferase	CZ	-0.47368	0.186904	b
Adenine phosphoribosyltransferase	DZ	-0.83542	0.124669	b
Adenine phosphoribosyltransferase	EZ	-0.49545	0.188166	b
Adenosine deaminase	AZ	-0.89759	0.158346	b
Adenosine deaminase	BZ	-0.92757	0.100243	b
Adenosine deaminase	CZ	0.582675	0.098058	a
Adenosine deaminase	DZ	0.777766	0.068548	a
Adenosine deaminase	EZ	0.464713	0.221905	a
Adenosine kinase	AZ	0.866836	0.103113	a
Adenosine kinase	BZ	0.96292	0.033339	a
Adenosine kinase	CZ	-0.38554	0.173326	b
Adenosine kinase	DZ	-0.76463	0.109367	b
Adenosine kinase	EZ	-0.67959	0.206313	b
Adenosinetriphosphatase	AZ	0.898478	0.085302	a
Adenosinetriphosphatase	BZ	0.970118	0.02882	a
Adenosinetriphosphatase	CZ	-0.44172	0.175429	b
Adenosinetriphosphatase	DZ	-0.78409	0.11285	b
Adenosinetriphosphatase	EZ	-0.64279	0.19952	b
Adenosylhomocysteinase	AZ	0.866802	0.103111	a
Adenosylhomocysteinase	BZ	0.962972	0.033353	a
Adenosylhomocysteinase	CZ	-0.38555	0.173323	b
Adenosylhomocysteinase	DZ	-0.76463	0.109365	b
Adenosylhomocysteinase	EZ	-0.67959	0.206309	b
Adenosylmethionine decarboxylase	AZ	0.866802	0.103111	a
Adenosylmethionine decarboxylase	BZ	0.962972	0.033353	a
Adenosylmethionine decarboxylase	CZ	-0.38555	0.173323	b
Adenosylmethionine decarboxylase	DZ	-0.76463	0.109365	b
Adenosylmethionine decarboxylase	EZ	-0.67959	0.206309	b
Adenosylmethionine--8-amino-7-oxononanoate transaminase	AZ	-0.9427	0.161457	b
Adenosylmethionine--8-amino-7-oxononanoate transaminase	BZ	-0.88965	0.091169	b
Adenosylmethionine--8-amino-7-oxononanoate transaminase	CZ	0.595373	0.107227	a
Adenosylmethionine--8-amino-7-oxononanoate transaminase	DZ	0.819858	0.059157	a
Adenosylmethionine--8-amino-7-oxononanoate transaminase	EZ	0.417115	0.215833	a
Adenylate cyclase	AZ	0.885221	0.078712	a
Adenylate cyclase	BZ	0.967134	0.022833	a
Adenylate cyclase	CZ	-0.37167	0.169601	b
Adenylate cyclase	DZ	-0.80297	0.131711	b
Adenylate cyclase	EZ	-0.67771	0.19777	b
Adenylate kinase	AZ	0.875087	0.095509	a

Adenylate kinase	BZ	0.975609	0.027489	a
Adenylate kinase	CZ	-0.36227	0.162888	b
Adenylate kinase	DZ	-0.81198	0.120773	b
Adenylate kinase	EZ	-0.67644	0.201786	b
Adenylosuccinate lyase	AZ	0.866802	0.103111	a
Adenylosuccinate lyase	BZ	0.962972	0.033353	a
Adenylosuccinate lyase	CZ	-0.38555	0.173323	b
Adenylosuccinate lyase	DZ	-0.76463	0.109365	b
Adenylosuccinate lyase	EZ	-0.67959	0.206309	b
Adenylosuccinate synthase	AZ	0.863605	0.092215	a
Adenylosuccinate synthase	BZ	0.915556	0.039169	a
Adenylosuccinate synthase	CZ	-0.10877	0.153303	b
Adenylosuccinate synthase	DZ	-0.97245	0.104261	b
Adenylosuccinate synthase	EZ	-0.69794	0.210134	b
Adenylyl-sulfate kinase	AZ	0.845264	0.106492	a
Adenylyl-sulfate kinase	BZ	0.956745	0.030162	a
Adenylyl-sulfate kinase	CZ	-0.32892	0.163326	b
Adenylyl-sulfate kinase	DZ	-0.86585	0.139926	b
Adenylyl-sulfate kinase	EZ	-0.60724	0.196653	b
ADP-ribose diphosphatase	AZ	-0.93422	0.15413	b
ADP-ribose diphosphatase	BZ	-0.91465	0.097549	b
ADP-ribose diphosphatase	CZ	0.593943	0.10242	a
ADP-ribose diphosphatase	DZ	0.812451	0.061338	a
ADP-ribose diphosphatase	EZ	0.442483	0.215814	a
ADP-sugar diphosphatase	AZ	-0.90944	0.121377	b
ADP-sugar diphosphatase	BZ	-0.91925	0.166545	b
ADP-sugar diphosphatase	CZ	0.670619	0.113368	a
ADP-sugar diphosphatase	DZ	0.584272	0.092779	a
ADP-sugar diphosphatase	EZ	0.573801	0.190911	a
Agmatinase	AZ	-0.91258	0.163373	b
Agmatinase	BZ	-0.9022	0.109204	b
Agmatinase	CZ	0.531128	0.094462	a
Agmatinase	DZ	0.810703	0.058727	a
Agmatinase	EZ	0.472953	0.220277	a
Agmatine deiminase	AZ	-0.88284	0.086441	b
Agmatine deiminase	BZ	-1.15767	0.059599	b
Agmatine deiminase	CZ	0.744477	0.101531	a
Agmatine deiminase	DZ	0.637049	0.091586	a
Agmatine deiminase	EZ	0.658991	0.18596	a
Alanine transaminase	AZ	0.866802	0.103111	a
Alanine transaminase	BZ	0.962972	0.033353	a
Alanine transaminase	CZ	-0.38555	0.173323	b
Alanine transaminase	DZ	-0.76463	0.109365	b
Alanine transaminase	EZ	-0.67959	0.206309	b

Alanine--glyoxylate transaminase	AZ	0.833315	0.092173	a
Alanine--glyoxylate transaminase	BZ	0.915836	0.029702	a
Alanine--glyoxylate transaminase	CZ	-0.38509	0.17604	b
Alanine--glyoxylate transaminase	DZ	-0.87405	0.176188	b
Alanine--glyoxylate transaminase	EZ	-0.49001	0.185238	b
Alanine--tRNA ligase	AZ	0.866921	0.103381	a
Alanine--tRNA ligase	BZ	0.963281	0.03345	a
Alanine--tRNA ligase	CZ	-0.38432	0.172752	b
Alanine--tRNA ligase	DZ	-0.76825	0.109787	b
Alanine--tRNA ligase	EZ	-0.67763	0.206081	b
Alcohol dehydrogenase	AZ	-0.89029	0.149466	b
Alcohol dehydrogenase	BZ	-0.94853	0.072014	b
Alcohol dehydrogenase	CZ	0.502801	0.132829	a
Alcohol dehydrogenase	DZ	0.769377	0.072391	a
Alcohol dehydrogenase	EZ	0.566642	0.21606	a
Alcohol dehydrogenase (NADP(+))	AZ	-0.52826	0.010335	b
Alcohol dehydrogenase (NADP(+))	BZ	-0.7596	0.085392	b
Alcohol dehydrogenase (NADP(+))	CZ	0.938726	0.179561	a
Alcohol dehydrogenase (NADP(+))	DZ	0.234359	0.254608	ab
Alcohol dehydrogenase (NADP(+))	EZ	0.11478	0.212757	ab
Aldehyde dehydrogenase (NAD(+))	AZ	0.77193	0.047319	a
Aldehyde dehydrogenase (NAD(+))	BZ	0.80499	0.03553	a
Aldehyde dehydrogenase (NAD(+))	CZ	-0.22309	0.11892	ab
Aldehyde dehydrogenase (NAD(+))	DZ	-0.36635	0.178952	b
Aldehyde dehydrogenase (NAD(+))	EZ	-0.98748	0.264922	b
Aldehyde dehydrogenase (NAD(P)(+))	AZ	-0.8968	0.156489	b
Aldehyde dehydrogenase (NAD(P)(+))	BZ	-0.95382	0.096224	b
Aldehyde dehydrogenase (NAD(P)(+))	CZ	0.636348	0.101717	a
Aldehyde dehydrogenase (NAD(P)(+))	DZ	0.770264	0.074567	a
Aldehyde dehydrogenase (NAD(P)(+))	EZ	0.444015	0.211835	a
Aldehyde reductase	AZ	0.793509	0.106619	a
Aldehyde reductase	BZ	0.90139	0.03699	a
Aldehyde reductase	CZ	-0.00339	0.096463	ab
Aldehyde reductase	DZ	-1.00496	0.146269	c
Aldehyde reductase	EZ	-0.68655	0.22448	bc
Aldose 1-epimerase	AZ	-0.84678	0.11255	b
Aldose 1-epimerase	BZ	-0.9787	0.033489	b
Aldose 1-epimerase	CZ	0.32916	0.122168	a
Aldose 1-epimerase	DZ	0.92763	0.160093	a
Aldose 1-epimerase	EZ	0.568689	0.193129	a
Alkaline phosphatase	AZ	0.760335	0.107797	a
Alkaline phosphatase	BZ	0.880953	0.030842	a
Alkaline phosphatase	CZ	-0.44873	0.215645	b
Alkaline phosphatase	DZ	-0.55206	0.180943	b

Alkaline phosphatase	EZ	-0.6405	0.186165	b
Alkane 1-monooxygenase	AZ	-0.88194	0.175392	b
Alkane 1-monooxygenase	BZ	-0.81252	0.114699	b
Alkane 1-monooxygenase	CZ	0.369332	0.167002	a
Alkane 1-monooxygenase	DZ	0.73498	0.105703	a
Alkane 1-monooxygenase	EZ	0.590156	0.184048	a
All-trans-retinol dehydrogenase (NAD(+))	AZ	0.735606	0.079903	a
All-trans-retinol dehydrogenase (NAD(+))	BZ	0.831071	0.020421	a
All-trans-retinol dehydrogenase (NAD(+))	CZ	-0.24118	0.223006	ab
All-trans-retinol dehydrogenase (NAD(+))	DZ	-0.82223	0.191062	b
All-trans-retinol dehydrogenase (NAD(+))	EZ	-0.50326	0.189728	b
Allantoate deiminase	AZ	-0.64945	0.05381	b
Allantoate deiminase	BZ	-1.32807	0.163081	b
Allantoate deiminase	CZ	0.694551	0.085139	a
Allantoate deiminase	DZ	0.593633	0.082142	a
Allantoate deiminase	EZ	0.689333	0.147433	a
Allantoicase	AZ	0.846958	0.098581	a
Allantoicase	BZ	0.972545	0.034316	a
Allantoicase	CZ	-0.43422	0.1869	b
Allantoicase	DZ	-0.78425	0.112824	b
Allantoicase	EZ	-0.60102	0.200106	b
Allantoin racemase	AZ	-0.72824	0.069862	b
Allantoin racemase	BZ	-0.77056	0.071886	b
Allantoin racemase	CZ	0.61804	0.033576	a
Allantoin racemase	DZ	-0.03068	0.280935	ab
Allantoin racemase	EZ	0.911445	0.181693	a
Allantoinase	AZ	-0.93769	0.154765	b
Allantoinase	BZ	-0.90066	0.100384	b
Allantoinase	CZ	0.549694	0.106665	a
Allantoinase	DZ	0.837492	0.048612	a
Allantoinase	EZ	0.451165	0.217606	a
Alpha-1,3-glucan synthase	AZ	-0.96581	0.147854	b
Alpha-1,3-glucan synthase	BZ	-0.83551	0.099379	b
Alpha-1,3-glucan synthase	CZ	0.549295	0.11721	a
Alpha-1,3-glucan synthase	DZ	0.816812	0.040505	a
Alpha-1,3-glucan synthase	EZ	0.435212	0.231139	a
Alpha-amylase	AZ	-0.93923	0.157641	b
Alpha-amylase	BZ	-0.86373	0.098542	b
Alpha-amylase	CZ	0.555636	0.114115	a
Alpha-amylase	DZ	0.80658	0.048207	a
Alpha-amylase	EZ	0.440743	0.225845	a
Alpha-galactosidase	AZ	-0.96468	0.159844	b
Alpha-galactosidase	BZ	-0.86409	0.089795	b
Alpha-galactosidase	CZ	0.559489	0.109343	a

Alpha-galactosidase	DZ	0.840332	0.039418	a
Alpha-galactosidase	EZ	0.428956	0.221029	a
Alpha-glucosidase	AZ	-0.7074	0.170056	b
Alpha-glucosidase	BZ	-0.83967	0.063687	b
Alpha-glucosidase	CZ	0.481847	0.177351	a
Alpha-glucosidase	DZ	0.603448	0.196551	a
Alpha-glucosidase	EZ	0.461776	0.190231	a
Alpha-glucuronidase	AZ	-0.85176	0.088806	b
Alpha-glucuronidase	BZ	-1.17153	0.052557	b
Alpha-glucuronidase	CZ	0.744305	0.114847	a
Alpha-glucuronidase	DZ	0.664138	0.100401	a
Alpha-glucuronidase	EZ	0.614842	0.180355	a
Alpha-L-fucosidase	AZ	-0.79773	0.172644	b
Alpha-L-fucosidase	BZ	-0.96153	0.109987	b
Alpha-L-fucosidase	CZ	0.68229	0.108077	a
Alpha-L-fucosidase	DZ	0.581401	0.084515	a
Alpha-L-fucosidase	EZ	0.495561	0.220844	a
Alpha-L-rhamnosidase	AZ	-0.87526	0.090746	b
Alpha-L-rhamnosidase	BZ	-1.14863	0.057666	b
Alpha-L-rhamnosidase	CZ	0.748858	0.108557	a
Alpha-L-rhamnosidase	DZ	0.65185	0.096379	a
Alpha-L-rhamnosidase	EZ	0.62318	0.185452	a
Alpha-mannosidase	AZ	0.80542	0.107552	a
Alpha-mannosidase	BZ	0.900695	0.036966	a
Alpha-mannosidase	CZ	-0.36344	0.173677	b
Alpha-mannosidase	DZ	-0.63419	0.168236	b
Alpha-mannosidase	EZ	-0.70849	0.209259	b
Alpha-methylacyl-CoA racemase	AZ	-0.84034	0.107333	b
Alpha-methylacyl-CoA racemase	BZ	-0.99083	0.028674	b
Alpha-methylacyl-CoA racemase	CZ	0.379832	0.178788	a
Alpha-methylacyl-CoA racemase	DZ	0.820751	0.105028	a
Alpha-methylacyl-CoA racemase	EZ	0.630583	0.198718	a
Alpha-N-acetylglucosaminidase	AZ	-0.99589	0.157305	b
Alpha-N-acetylglucosaminidase	BZ	-0.70972	0.126247	b
Alpha-N-acetylglucosaminidase	CZ	0.280344	0.103604	a
Alpha-N-acetylglucosaminidase	DZ	0.996849	0.059628	a
Alpha-N-acetylglucosaminidase	EZ	0.428411	0.217856	a
Alpha,alpha-phosphotrehalase	AZ	-0.87941	0.134089	b
Alpha,alpha-phosphotrehalase	BZ	-0.38624	0.146626	b
Alpha,alpha-phosphotrehalase	CZ	0.273466	0.184423	ab
Alpha,alpha-phosphotrehalase	DZ	0.115406	0.155456	ab
Alpha,alpha-phosphotrehalase	EZ	0.876778	0.231027	a
Alpha,alpha-trehalase	AZ	0.836913	0.112511	a
Alpha,alpha-trehalase	BZ	0.955829	0.035861	a

Alpha,alpha-trehalase	CZ	-0.37811	0.165941	b
Alpha,alpha-trehalase	DZ	-0.86194	0.121365	b
Alpha,alpha-trehalase	EZ	-0.55269	0.208612	b
Amidase	AZ	-0.93997	0.15592	b
Amidase	BZ	-0.88479	0.096711	b
Amidase	CZ	0.593855	0.104979	a
Amidase	DZ	0.737203	0.061482	a
Amidase	EZ	0.4937	0.226323	a
Amidophosphoribosyltransferase	AZ	0.861107	0.107153	a
Amidophosphoribosyltransferase	BZ	0.961047	0.034661	a
Amidophosphoribosyltransferase	CZ	-0.43803	0.179481	b
Amidophosphoribosyltransferase	DZ	-0.77577	0.122213	b
Amidophosphoribosyltransferase	EZ	-0.60835	0.19678	b
Amino-acid N-acetyltransferase	AZ	0.8788	0.10373	a
Amino-acid N-acetyltransferase	BZ	0.940157	0.040595	a
Amino-acid N-acetyltransferase	CZ	-0.37448	0.173266	b
Amino-acid N-acetyltransferase	DZ	-0.75024	0.108158	b
Amino-acid N-acetyltransferase	EZ	-0.69424	0.20931	b
Aminoacyl-tRNA hydrolase	AZ	0.873553	0.101552	a
Aminoacyl-tRNA hydrolase	BZ	0.974185	0.031012	a
Aminoacyl-tRNA hydrolase	CZ	-0.47708	0.179476	b
Aminoacyl-tRNA hydrolase	DZ	-0.69822	0.106369	b
Aminoacyl-tRNA hydrolase	EZ	-0.67244	0.203048	b
Aminobutyraldehyde dehydrogenase	AZ	0.883301	0.066085	a
Aminobutyraldehyde dehydrogenase	BZ	0.894713	0.031558	a
Aminobutyraldehyde dehydrogenase	CZ	-0.31346	0.197802	b
Aminobutyraldehyde dehydrogenase	DZ	-0.82363	0.117642	b
Aminobutyraldehyde dehydrogenase	EZ	-0.64093	0.208002	b
Aminocarboxymuconate-semialdehyde decarboxylase	AZ	-0.91652	0.159754	b
Aminocarboxymuconate-semialdehyde decarboxylase	BZ	-0.86667	0.102834	b
Aminocarboxymuconate-semialdehyde decarboxylase	CZ	0.514978	0.109972	a
Aminocarboxymuconate-semialdehyde decarboxylase	DZ	0.803359	0.055229	a
Aminocarboxymuconate-semialdehyde decarboxylase	EZ	0.464859	0.229932	a
Aminomethyltransferase	AZ	0.875705	0.076128	a
Aminomethyltransferase	BZ	0.95596	0.023849	a
Aminomethyltransferase	CZ	-0.26731	0.153823	b
Aminomethyltransferase	DZ	-0.89716	0.134624	b
Aminomethyltransferase	EZ	-0.66719	0.204303	b
Aminopeptidase Y	AZ	-0.86897	0.13411	b
Aminopeptidase Y	BZ	-0.968	0.099594	b
Aminopeptidase Y	CZ	0.560967	0.083989	a
Aminopeptidase Y	DZ	0.553622	0.149273	a
Aminopeptidase Y	EZ	0.722378	0.20249	a
AMP deaminase	AZ	0.869025	0.10533	a

AMP deaminase	BZ	0.963664	0.034347	a
AMP deaminase	CZ	-0.40843	0.176468	b
AMP deaminase	DZ	-0.78356	0.110293	b
AMP deaminase	EZ	-0.64071	0.201652	b
Anthranilate phosphoribosyltransferase	AZ	0.742783	0.098462	a
Anthranilate phosphoribosyltransferase	BZ	0.982803	0.032113	a
Anthranilate phosphoribosyltransferase	CZ	-0.32153	0.168088	b
Anthranilate phosphoribosyltransferase	DZ	-0.63433	0.169481	b
Anthranilate phosphoribosyltransferase	EZ	-0.76973	0.203724	b
Anthranilate synthase	AZ	-0.91493	0.034543	b
Anthranilate synthase	BZ	-0.88388	0.01898	b
Anthranilate synthase	CZ	0.429592	0.215576	a
Anthranilate synthase	DZ	0.794756	0.137551	a
Anthranilate synthase	EZ	0.574459	0.18662	a
Apyrase	AZ	0.88203	0.058057	a
Apyrase	BZ	0.889519	0.023205	a
Apyrase	CZ	-0.29867	0.13967	b
Apyrase	DZ	-0.89417	0.203732	b
Apyrase	EZ	-0.57871	0.188238	b
Arabinan endo-1,5-alpha-L-arabinosidase	AZ	-0.90106	0.10254	b
Arabinan endo-1,5-alpha-L-arabinosidase	BZ	-1.01361	0.134814	b
Arabinan endo-1,5-alpha-L-arabinosidase	CZ	0.680444	0.119216	a
Arabinan endo-1,5-alpha-L-arabinosidase	DZ	0.661577	0.087218	a
Arabinan endo-1,5-alpha-L-arabinosidase	EZ	0.572647	0.189987	a
Arabinogalactan endo-beta-1,4-galactanase	AZ	-0.912	0.166805	b
Arabinogalactan endo-beta-1,4-galactanase	BZ	-0.89661	0.089719	b
Arabinogalactan endo-beta-1,4-galactanase	CZ	0.513202	0.09356	a
Arabinogalactan endo-beta-1,4-galactanase	DZ	0.862571	0.05819	a
Arabinogalactan endo-beta-1,4-galactanase	EZ	0.432838	0.224648	a
Arabinose-5-phosphate isomerase	AZ	-0.84104	0.193978	c
Arabinose-5-phosphate isomerase	BZ	-0.68239	0.188306	bc
Arabinose-5-phosphate isomerase	CZ	0.437362	0.118883	ab
Arabinose-5-phosphate isomerase	DZ	0.657	0.149206	a
Arabinose-5-phosphate isomerase	EZ	0.429065	0.177528	ab
Arachidonate 15-lipoxygenase	AZ	-1.01217	0.142246	b
Arachidonate 15-lipoxygenase	BZ	-0.35808	0.189886	ab
Arachidonate 15-lipoxygenase	CZ	0.234968	0.16442	a
Arachidonate 15-lipoxygenase	DZ	0.710749	0.126142	a
Arachidonate 15-lipoxygenase	EZ	0.424533	0.214705	a
Aralkylamine N-acetyltransferase	AZ	-0.91106	0.162697	b
Aralkylamine N-acetyltransferase	BZ	-0.87113	0.106174	b
Aralkylamine N-acetyltransferase	CZ	0.576124	0.101008	a
Aralkylamine N-acetyltransferase	DZ	0.778292	0.07561	a
Aralkylamine N-acetyltransferase	EZ	0.427773	0.225522	a

Arginase	AZ	0.531459	0.086381	a
Arginase	BZ	0.563479	0.043507	a
Arginase	CZ	0.457826	0.240053	a
Arginase	DZ	-1.21652	0.130166	b
Arginase	EZ	-0.33625	0.189605	ab
Argininosuccinate synthase	AZ	0.843346	0.101536	a
Argininosuccinate synthase	BZ	0.913378	0.039944	a
Argininosuccinate synthase	CZ	-0.14373	0.172863	b
Argininosuccinate synthase	DZ	-1.01319	0.111589	b
Argininosuccinate synthase	EZ	-0.59981	0.195289	b
Arginyltransferase	AZ	0.866659	0.102881	a
Arginyltransferase	BZ	0.962763	0.033284	a
Arginyltransferase	CZ	-0.38295	0.172962	b
Arginyltransferase	DZ	-0.76987	0.110636	b
Arginyltransferase	EZ	-0.6766	0.205898	b
Aromatic-L-amino-acid decarboxylase	AZ	0.870638	0.093979	a
Aromatic-L-amino-acid decarboxylase	BZ	0.878258	0.04422	a
Aromatic-L-amino-acid decarboxylase	CZ	-0.1055	0.157248	b
Aromatic-L-amino-acid decarboxylase	DZ	-0.86448	0.112855	b
Aromatic-L-amino-acid decarboxylase	EZ	-0.77892	0.216849	b
Arsenite methyltransferase	AZ	-1.12432	0.147484	b
Arsenite methyltransferase	BZ	-0.66541	0.095025	b
Arsenite methyltransferase	CZ	0.564915	0.12373	a
Arsenite methyltransferase	DZ	0.751213	0.054236	a
Arsenite methyltransferase	EZ	0.473605	0.224137	a
Arsenite-transporting ATPase	AZ	0.866641	0.102984	a
Arsenite-transporting ATPase	BZ	0.962692	0.033312	a
Arsenite-transporting ATPase	CZ	-0.38728	0.173973	b
Arsenite-transporting ATPase	DZ	-0.76278	0.10923	b
Arsenite-transporting ATPase	EZ	-0.67928	0.206236	b
Aryl-alcohol dehydrogenase (NADP(+))	AZ	-0.92898	0.143861	b
Aryl-alcohol dehydrogenase (NADP(+))	BZ	-0.85247	0.083249	b
Aryl-alcohol dehydrogenase (NADP(+))	CZ	0.219757	0.139053	a
Aryl-alcohol dehydrogenase (NADP(+))	DZ	1.091646	0.043192	a
Aryl-alcohol dehydrogenase (NADP(+))	EZ	0.470056	0.196568	a
Arylamine N-acetyltransferase	AZ	-0.87352	0.155856	b
Arylamine N-acetyltransferase	BZ	-0.92193	0.09937	b
Arylamine N-acetyltransferase	CZ	0.540936	0.09249	a
Arylamine N-acetyltransferase	DZ	0.781844	0.078542	a
Arylamine N-acetyltransferase	EZ	0.472669	0.232586	a
Arylformamidase	AZ	-0.92228	0.149612	b
Arylformamidase	BZ	-1.00836	0.076179	b
Arylformamidase	CZ	0.546806	0.109475	a
Arylformamidase	DZ	0.842484	0.11135	a

Arylformamidase	EZ	0.541348	0.170201	a
Arylsulfatase	AZ	-0.97632	0.158391	b
Arylsulfatase	BZ	-0.80861	0.099329	b
Arylsulfatase	CZ	0.552167	0.110364	a
Arylsulfatase	DZ	0.801388	0.052739	a
Arylsulfatase	EZ	0.431369	0.230375	a
Asparaginase	AZ	-0.8842	0.110601	b
Asparaginase	BZ	-0.96548	0.04262	b
Asparaginase	CZ	0.458214	0.160136	a
Asparaginase	DZ	0.807971	0.115746	a
Asparaginase	EZ	0.583498	0.202607	a
Asparagine synthase (glutamine-hydrolyzing)	AZ	-0.8403	0.114027	b
Asparagine synthase (glutamine-hydrolyzing)	BZ	-0.9392	0.054708	b
Asparagine synthase (glutamine-hydrolyzing)	CZ	0.331036	0.120993	a
Asparagine synthase (glutamine-hydrolyzing)	DZ	0.879047	0.170206	a
Asparagine synthase (glutamine-hydrolyzing)	EZ	0.569419	0.201638	a
Asparagine--tRNA ligase	AZ	0.867961	0.102485	a
Asparagine--tRNA ligase	BZ	0.962087	0.033225	a
Asparagine--tRNA ligase	CZ	-0.37875	0.172483	b
Asparagine--tRNA ligase	DZ	-0.77404	0.110452	b
Asparagine--tRNA ligase	EZ	-0.67726	0.205988	b
Aspartate kinase	AZ	0.849117	0.094825	a
Aspartate kinase	BZ	0.920569	0.036605	a
Aspartate kinase	CZ	-0.07753	0.146605	b
Aspartate kinase	DZ	-0.93681	0.110797	b
Aspartate kinase	EZ	-0.75535	0.211585	b
Aspartate--tRNA ligase	AZ	-0.86215	0.084155	b
Aspartate--tRNA ligase	BZ	-0.88649	0.04549	b
Aspartate--tRNA ligase	CZ	0.273846	0.164365	a
Aspartate--tRNA ligase	DZ	1.043286	0.155505	a
Aspartate--tRNA ligase	EZ	0.431503	0.188975	a
Aspartate-semialdehyde dehydrogenase	AZ	0.848002	0.086503	a
Aspartate-semialdehyde dehydrogenase	BZ	0.902721	0.032569	a
Aspartate-semialdehyde dehydrogenase	CZ	-0.31661	0.174151	b
Aspartate-semialdehyde dehydrogenase	DZ	-0.62862	0.093448	b
Aspartate-semialdehyde dehydrogenase	EZ	-0.8055	0.241833	b
Aspartyl aminopeptidase	AZ	0.869743	0.1032	a
Aspartyl aminopeptidase	BZ	0.965996	0.033382	a
Aspartyl aminopeptidase	CZ	-0.39257	0.170491	b
Aspartyl aminopeptidase	DZ	-0.76424	0.109688	b
Aspartyl aminopeptidase	EZ	-0.67892	0.206642	b
Assimilatory sulfite reductase (NADPH)	AZ	-0.9485	0.13672	b
Assimilatory sulfite reductase (NADPH)	BZ	-0.9462	0.1422	b
Assimilatory sulfite reductase (NADPH)	CZ	0.691779	0.119674	a

Assimilatory sulfite reductase (NADPH)	DZ	0.748346	0.055699	a
Assimilatory sulfite reductase (NADPH)	EZ	0.454571	0.177919	a
ATP adenylyltransferase	AZ	0.783189	0.106613	a
ATP adenylyltransferase	BZ	0.928594	0.043412	a
ATP adenylyltransferase	CZ	-0.17708	0.187515	b
ATP adenylyltransferase	DZ	-1.04292	0.118964	b
ATP adenylyltransferase	EZ	-0.49179	0.188697	b
ATP phosphoribosyltransferase	AZ	0.866892	0.103202	a
ATP phosphoribosyltransferase	BZ	0.962998	0.033377	a
ATP phosphoribosyltransferase	CZ	-0.38329	0.172762	b
ATP phosphoribosyltransferase	DZ	-0.76585	0.109444	b
ATP phosphoribosyltransferase	EZ	-0.68075	0.206457	b
ATP-dependent NAD(P)H-hydrate dehydratase	AZ	-0.89015	0.116878	b
ATP-dependent NAD(P)H-hydrate dehydratase	BZ	-0.97146	0.155143	b
ATP-dependent NAD(P)H-hydrate dehydratase	CZ	0.64138	0.1246	a
ATP-dependent NAD(P)H-hydrate dehydratase	DZ	0.692759	0.082785	a
ATP-dependent NAD(P)H-hydrate dehydratase	EZ	0.527478	0.186038	a
Benzaldehyde dehydrogenase (NAD(+))	AZ	-0.93908	0.158005	b
Benzaldehyde dehydrogenase (NAD(+))	BZ	-0.81562	0.102027	b
Benzaldehyde dehydrogenase (NAD(+))	CZ	0.479992	0.116097	a
Benzaldehyde dehydrogenase (NAD(+))	DZ	0.830058	0.055987	a
Benzaldehyde dehydrogenase (NAD(+))	EZ	0.444651	0.234097	a
Benzene 1,2-dioxygenase	AZ	-1.02366	0.154132	c
Benzene 1,2-dioxygenase	BZ	-0.59447	0.126501	bc
Benzene 1,2-dioxygenase	CZ	0.586184	0.193128	a
Benzene 1,2-dioxygenase	DZ	0.714372	0.047211	a
Benzene 1,2-dioxygenase	EZ	0.317569	0.207149	ab
Benzoate 4-monooxygenase	AZ	-0.91826	0.114315	b
Benzoate 4-monooxygenase	BZ	-0.94875	0.155933	b
Benzoate 4-monooxygenase	CZ	0.663864	0.113568	a
Benzoate 4-monooxygenase	DZ	0.641874	0.079349	a
Benzoate 4-monooxygenase	EZ	0.561275	0.194599	a
Beta-1,4-mannosyl-glycoprotein 4-beta-N-acetylglucosaminyltransferase	AZ	-0.80984	0.223631	b
Beta-1,4-mannosyl-glycoprotein 4-beta-N-acetylglucosaminyltransferase	BZ	-0.67046	0.122551	b
Beta-1,4-mannosyl-glycoprotein 4-beta-N-acetylglucosaminyltransferase	CZ	0.721507	0.178104	a
Beta-1,4-mannosyl-glycoprotein 4-beta-N-acetylglucosaminyltransferase	DZ	0.244861	0.16179	ab
Beta-1,4-mannosyl-glycoprotein 4-beta-N-acetylglucosaminyltransferase	EZ	0.513924	0.139906	a
Beta-fructofuranosidase	AZ	-0.96576	0.167019	b
Beta-fructofuranosidase	BZ	-0.77107	0.110724	b
Beta-fructofuranosidase	CZ	0.604078	0.126135	a
Beta-fructofuranosidase	DZ	0.753308	0.039894	a
Beta-fructofuranosidase	EZ	0.37944	0.228393	a
Beta-galactosidase	AZ	-0.94011	0.155754	b
Beta-galactosidase	BZ	-0.8924	0.091671	b

Beta-galactosidase	CZ	0.554047	0.101426	a
Beta-galactosidase	DZ	0.815657	0.055171	a
Beta-galactosidase	EZ	0.462809	0.225108	a
Beta-glucosidase	AZ	-0.91833	0.150375	b
Beta-glucosidase	BZ	-0.94213	0.071534	b
Beta-glucosidase	CZ	0.531486	0.126913	a
Beta-glucosidase	DZ	0.818262	0.059087	a
Beta-glucosidase	EZ	0.510715	0.213265	a
Beta-glucuronidase	AZ	-0.78492	0.170849	b
Beta-glucuronidase	BZ	-0.9597	0.111112	b
Beta-glucuronidase	CZ	0.582437	0.104676	a
Beta-glucuronidase	DZ	0.723002	0.084933	a
Beta-glucuronidase	EZ	0.439177	0.225158	a
Beta-ketoacyl-[acyl-carrier-protein] synthase I	AZ	0.853321	0.074439	a
Beta-ketoacyl-[acyl-carrier-protein] synthase I	BZ	0.923557	0.022738	a
Beta-ketoacyl-[acyl-carrier-protein] synthase I	CZ	-0.65572	0.240464	b
Beta-ketoacyl-[acyl-carrier-protein] synthase I	DZ	-0.64503	0.11365	b
Beta-ketoacyl-[acyl-carrier-protein] synthase I	EZ	-0.47613	0.174672	b
Beta-lactamase	AZ	-0.95693	0.167052	b
Beta-lactamase	BZ	-0.77153	0.105531	b
Beta-lactamase	CZ	0.483713	0.108949	a
Beta-lactamase	DZ	0.778946	0.053787	a
Beta-lactamase	EZ	0.465805	0.240332	a
Beta-mannosidase	AZ	-0.94505	0.157612	b
Beta-mannosidase	BZ	-0.89744	0.090853	b
Beta-mannosidase	CZ	0.540774	0.102915	a
Beta-mannosidase	DZ	0.82271	0.054391	a
Beta-mannosidase	EZ	0.479005	0.220209	a
Beta-N-acetylhexosaminidase	AZ	-0.92382	0.03554	b
Beta-N-acetylhexosaminidase	BZ	-0.86131	0.022823	b
Beta-N-acetylhexosaminidase	CZ	0.565215	0.181031	a
Beta-N-acetylhexosaminidase	DZ	0.747751	0.212259	a
Beta-N-acetylhexosaminidase	EZ	0.472166	0.159837	a
Beta-ureidopropionase	AZ	-0.96699	0.159129	b
Beta-ureidopropionase	BZ	-0.78669	0.106432	b
Beta-ureidopropionase	CZ	0.516784	0.114063	a
Beta-ureidopropionase	DZ	0.754331	0.048544	a
Beta-ureidopropionase	EZ	0.482558	0.23841	a
Betaine-aldehyde dehydrogenase	AZ	-0.88799	0.142415	b
Betaine-aldehyde dehydrogenase	BZ	-0.99847	0.108184	b
Betaine-aldehyde dehydrogenase	CZ	0.600539	0.062606	a
Betaine-aldehyde dehydrogenase	DZ	0.885966	0.096509	a
Betaine-aldehyde dehydrogenase	EZ	0.399955	0.200574	a
Biotin synthase	AZ	0.860426	0.108912	a

Biotin synthase	BZ	0.960778	0.035623	a
Biotin synthase	CZ	-0.42304	0.181638	b
Biotin synthase	DZ	-0.81998	0.111067	b
Biotin synthase	EZ	-0.57818	0.197707	b
Bis(5'-adenosyl)-triphosphatase	AZ	0.803174	0.086188	a
Bis(5'-adenosyl)-triphosphatase	BZ	0.900475	0.024597	a
Bis(5'-adenosyl)-triphosphatase	CZ	-0.17396	0.149048	b
Bis(5'-adenosyl)-triphosphatase	DZ	-0.65147	0.145705	b
Bis(5'-adenosyl)-triphosphatase	EZ	-0.87822	0.233295	b
Bis(5'-nucleosyl)-tetraphosphatase (asymmetrical)	AZ	-0.91495	0.116605	b
Bis(5'-nucleosyl)-tetraphosphatase (asymmetrical)	BZ	-0.96731	0.042308	b
Bis(5'-nucleosyl)-tetraphosphatase (asymmetrical)	CZ	0.36357	0.161124	a
Bis(5'-nucleosyl)-tetraphosphatase (asymmetrical)	DZ	0.978369	0.051947	a
Bis(5'-nucleosyl)-tetraphosphatase (asymmetrical)	EZ	0.540323	0.195565	a
Bis(5'-nucleosyl)-tetraphosphatase (symmetrical)	AZ	-1.07799	0.181277	c
Bis(5'-nucleosyl)-tetraphosphatase (symmetrical)	BZ	-0.63264	0.096381	bc
Bis(5'-nucleosyl)-tetraphosphatase (symmetrical)	CZ	0.516738	0.129993	a
Bis(5'-nucleosyl)-tetraphosphatase (symmetrical)	DZ	0.959188	0.050958	a
Bis(5'-nucleosyl)-tetraphosphatase (symmetrical)	EZ	0.2347	0.195277	ab
Bleomycin hydrolase	AZ	-0.85367	0.152406	b
Bleomycin hydrolase	BZ	-0.98439	0.109468	b
Bleomycin hydrolase	CZ	0.505836	0.107995	a
Bleomycin hydrolase	DZ	0.837481	0.054329	a
Bleomycin hydrolase	EZ	0.494751	0.212565	a
Branched-chain-amino-acid transaminase	AZ	-0.85912	0.1255	b
Branched-chain-amino-acid transaminase	BZ	-1.0001	0.048696	b
Branched-chain-amino-acid transaminase	CZ	0.468714	0.131872	a
Branched-chain-amino-acid transaminase	DZ	0.731775	0.114423	a
Branched-chain-amino-acid transaminase	EZ	0.658728	0.212193	a
Butyrate--CoA ligase	AZ	-1.11397	0.117556	b
Butyrate--CoA ligase	BZ	-0.64405	0.074623	b
Butyrate--CoA ligase	CZ	1.004513	0.097777	a
Butyrate--CoA ligase	DZ	0.311575	0.197385	a
Butyrate--CoA ligase	EZ	0.441931	0.155669	a
Caffeoyl-CoA O-methyltransferase	AZ	-1.04818	0.249568	c
Caffeoyl-CoA O-methyltransferase	BZ	-0.45756	0.137129	bc
Caffeoyl-CoA O-methyltransferase	CZ	0.138949	0.064539	ab
Caffeoyl-CoA O-methyltransferase	DZ	1.045825	0.110335	a
Caffeoyl-CoA O-methyltransferase	EZ	0.320966	0.127931	ab
Calcium-transporting ATPase	AZ	0.828478	0.110974	a
Calcium-transporting ATPase	BZ	0.9698	0.035176	a
Calcium-transporting ATPase	CZ	-0.25129	0.147256	b
Calcium-transporting ATPase	DZ	-0.80646	0.128789	b
Calcium-transporting ATPase	EZ	-0.74053	0.209134	b

Calmodulin-lysine N-methyltransferase	AZ	-0.8935	0.092213	b
Calmodulin-lysine N-methyltransferase	BZ	-1.12663	0.062809	b
Calmodulin-lysine N-methyltransferase	CZ	0.75014	0.104915	a
Calmodulin-lysine N-methyltransferase	DZ	0.639795	0.093471	a
Calmodulin-lysine N-methyltransferase	EZ	0.630199	0.189646	a
Carbamoyl-phosphate synthase (glutamine-hydrolyzing)	AZ	0.852229	0.098656	a
Carbamoyl-phosphate synthase (glutamine-hydrolyzing)	BZ	0.960514	0.028958	a
Carbamoyl-phosphate synthase (glutamine-hydrolyzing)	CZ	-0.27918	0.151455	b
Carbamoyl-phosphate synthase (glutamine-hydrolyzing)	DZ	-0.84297	0.13838	b
Carbamoyl-phosphate synthase (glutamine-hydrolyzing)	EZ	-0.6906	0.204019	b
Carbonate dehydratase	AZ	-0.7817	0.093503	bc
Carbonate dehydratase	BZ	-0.93459	0.030458	c
Carbonate dehydratase	CZ	0.095447	0.105928	ab
Carbonate dehydratase	DZ	0.972975	0.201499	a
Carbonate dehydratase	EZ	0.647867	0.188429	a
Carbonyl reductase (NADPH)	AZ	-0.8701	0.147846	b
Carbonyl reductase (NADPH)	BZ	-0.93194	0.073231	b
Carbonyl reductase (NADPH)	CZ	0.488082	0.149747	a
Carbonyl reductase (NADPH)	DZ	0.70424	0.066614	a
Carbonyl reductase (NADPH)	EZ	0.609717	0.222129	a
Carboxylesterase	AZ	-0.86904	0.151543	b
Carboxylesterase	BZ	-0.94298	0.067977	b
Carboxylesterase	CZ	0.451024	0.152925	a
Carboxylesterase	DZ	0.758872	0.041708	a
Carboxylesterase	EZ	0.602123	0.219028	a
Carboxymethylenebutenolidase	AZ	-0.90879	0.142338	b
Carboxymethylenebutenolidase	BZ	-0.97737	0.121746	b
Carboxymethylenebutenolidase	CZ	0.624968	0.090061	a
Carboxymethylenebutenolidase	DZ	0.735784	0.058956	a
Carboxymethylenebutenolidase	EZ	0.525408	0.209458	a
Carboxypeptidase A	AZ	-0.70838	0.122722	b
Carboxypeptidase A	BZ	-0.67116	0.04083	b
Carboxypeptidase A	CZ	0.480617	0.14755	ab
Carboxypeptidase A	DZ	0.751281	0.162456	a
Carboxypeptidase A	EZ	0.147644	0.294339	ab
Carboxypeptidase C	AZ	-0.83401	0.142075	b
Carboxypeptidase C	BZ	-0.97917	0.045413	b
Carboxypeptidase C	CZ	0.400749	0.136375	a
Carboxypeptidase C	DZ	0.795375	0.124936	a
Carboxypeptidase C	EZ	0.61706	0.205252	a
Carboxypeptidase D	AZ	-0.71676	0.088688	bc
Carboxypeptidase D	BZ	-0.84152	0.021971	c
Carboxypeptidase D	CZ	0.323376	0.195313	ab
Carboxypeptidase D	DZ	0.730483	0.236963	a

Carboxypeptidase D	EZ	0.504424	0.177839	a
Carnitine O-acetyltransferase	AZ	0.865964	0.086221	a
Carnitine O-acetyltransferase	BZ	0.965725	0.025132	a
Carnitine O-acetyltransferase	CZ	-0.32979	0.158751	b
Carnitine O-acetyltransferase	DZ	-0.73239	0.129987	b
Carnitine O-acetyltransferase	EZ	-0.76951	0.209391	b
Carnosine synthase	AZ	-0.61321	0.058247	b
Carnosine synthase	BZ	-0.80309	0.083048	b
Carnosine synthase	CZ	0.582324	0.021245	a
Carnosine synthase	DZ	-0.06382	0.306636	ab
Carnosine synthase	EZ	0.897801	0.163471	a
Catalase	AZ	-0.89071	0.155718	b
Catalase	BZ	-0.93413	0.102186	b
Catalase	CZ	0.559919	0.101042	a
Catalase	DZ	0.7776	0.063099	a
Catalase	EZ	0.487328	0.22369	a
Catechol 1,2-dioxygenase	AZ	-0.93214	0.116503	b
Catechol 1,2-dioxygenase	BZ	-0.94433	0.158955	b
Catechol 1,2-dioxygenase	CZ	0.661205	0.110203	a
Catechol 1,2-dioxygenase	DZ	0.617262	0.068915	a
Catechol 1,2-dioxygenase	EZ	0.597995	0.193507	a
Catechol O-methyltransferase	AZ	-0.92601	0.162823	b
Catechol O-methyltransferase	BZ	-0.83901	0.105833	b
Catechol O-methyltransferase	CZ	0.552196	0.106975	a
Catechol O-methyltransferase	DZ	0.747248	0.064266	a
Catechol O-methyltransferase	EZ	0.465579	0.23375	a
CDP-diacylglycerol--glycerol-3-phosphate 3-phosphatidyltransferase	AZ	0.866802	0.103111	a
CDP-diacylglycerol--glycerol-3-phosphate 3-phosphatidyltransferase	BZ	0.962972	0.033353	a
CDP-diacylglycerol--glycerol-3-phosphate 3-phosphatidyltransferase	CZ	-0.38555	0.173323	b
CDP-diacylglycerol--glycerol-3-phosphate 3-phosphatidyltransferase	DZ	-0.76463	0.109365	b
CDP-diacylglycerol--glycerol-3-phosphate 3-phosphatidyltransferase	EZ	-0.67959	0.206309	b
CDP-diacylglycerol--inositol 3-phosphatidyltransferase	AZ	0.739046	0.106773	a
CDP-diacylglycerol--inositol 3-phosphatidyltransferase	BZ	0.992872	0.036205	a
CDP-diacylglycerol--inositol 3-phosphatidyltransferase	CZ	-0.37641	0.17688	b
CDP-diacylglycerol--inositol 3-phosphatidyltransferase	DZ	-0.61917	0.158382	b
CDP-diacylglycerol--inositol 3-phosphatidyltransferase	EZ	-0.73633	0.202376	b
CDP-diacylglycerol--serine O-phosphatidyltransferase	AZ	0.88905	0.089029	a
CDP-diacylglycerol--serine O-phosphatidyltransferase	BZ	0.916993	0.03903	a
CDP-diacylglycerol--serine O-phosphatidyltransferase	CZ	-0.4202	0.202394	b
CDP-diacylglycerol--serine O-phosphatidyltransferase	DZ	-0.73718	0.088022	b
CDP-diacylglycerol--serine O-phosphatidyltransferase	EZ	-0.64867	0.207935	b
Cellobiose dehydrogenase (acceptor)	AZ	-0.88929	0.095777	b
Cellobiose dehydrogenase (acceptor)	BZ	-1.11157	0.081557	b
Cellobiose dehydrogenase (acceptor)	CZ	0.690808	0.106336	a

Cellobiose dehydrogenase (acceptor)	DZ	0.696123	0.084104	a
Cellobiose dehydrogenase (acceptor)	EZ	0.613933	0.193745	a
Cellulase	AZ	-0.93911	0.120853	b
Cellulase	BZ	-1.05544	0.089299	b
Cellulase	CZ	0.784967	0.105201	a
Cellulase	DZ	0.688178	0.061458	a
Cellulase	EZ	0.52141	0.186279	a
Cellulose 1,4-beta-cellobiosidase (non-reducing end)	AZ	-0.87495	0.090831	b
Cellulose 1,4-beta-cellobiosidase (non-reducing end)	BZ	-1.14858	0.05772	b
Cellulose 1,4-beta-cellobiosidase (non-reducing end)	CZ	0.750689	0.108659	a
Cellulose 1,4-beta-cellobiosidase (non-reducing end)	DZ	0.651431	0.096985	a
Cellulose 1,4-beta-cellobiosidase (non-reducing end)	EZ	0.621407	0.185107	a
Ceramidase	AZ	-0.96926	0.119314	b
Ceramidase	BZ	-0.97287	0.11533	b
Ceramidase	CZ	0.748673	0.073552	a
Ceramidase	DZ	0.739517	0.066878	a
Ceramidase	EZ	0.453943	0.206423	a
Ceramide glucosyltransferase	AZ	0.878944	0.103435	a
Ceramide glucosyltransferase	BZ	0.939538	0.040334	a
Ceramide glucosyltransferase	CZ	-0.37409	0.173512	b
Ceramide glucosyltransferase	DZ	-0.75433	0.109319	b
Ceramide glucosyltransferase	EZ	-0.69006	0.208798	b
Cerebroside-sulfatase	AZ	-1.03319	0.179349	b
Cerebroside-sulfatase	BZ	-0.46486	0.148758	ab
Cerebroside-sulfatase	CZ	0.68236	0.189009	a
Cerebroside-sulfatase	DZ	0.595744	0.088917	a
Cerebroside-sulfatase	EZ	0.219952	0.185607	a
Cerevisin	AZ	0.873493	0.07841	a
Cerevisin	BZ	0.879958	0.038404	a
Cerevisin	CZ	-0.47995	0.221295	b
Cerevisin	DZ	-0.62319	0.115737	b
Cerevisin	EZ	-0.65031	0.202642	b
Chitin deacetylase	AZ	0.834391	0.089542	ab
Chitin deacetylase	BZ	0.916393	0.029315	a
Chitin deacetylase	CZ	-0.04244	0.120766	bc
Chitin deacetylase	DZ	-0.92871	0.148047	c
Chitin deacetylase	EZ	-0.77963	0.210273	c
Chitin synthase	AZ	0.872745	0.08873	a
Chitin synthase	BZ	0.966543	0.026637	a
Chitin synthase	CZ	-0.3157	0.155651	b
Chitin synthase	DZ	-0.77076	0.125815	b
Chitin synthase	EZ	-0.75282	0.208667	b
Chitobiosyldiphosphodolichol beta-mannosyltransferase	AZ	0.867146	0.103132	a
Chitobiosyldiphosphodolichol beta-mannosyltransferase	BZ	0.96246	0.033215	a

Chitobiosyldiphosphodolichol beta-mannosyltransferase	CZ	-0.38545	0.173357	b
Chitobiosyldiphosphodolichol beta-mannosyltransferase	DZ	-0.7646	0.109387	b
Chitobiosyldiphosphodolichol beta-mannosyltransferase	EZ	-0.67955	0.206349	b
Chitosanase	AZ	-0.92726	0.087697	b
Chitosanase	BZ	-1.1131	0.069157	b
Chitosanase	CZ	0.739852	0.089107	a
Chitosanase	DZ	0.60104	0.080841	a
Chitosanase	EZ	0.699463	0.195494	a
Cholestenol Delta-isomerase	AZ	-0.88439	0.087917	b
Cholestenol Delta-isomerase	BZ	-0.97469	0.038674	b
Cholestenol Delta-isomerase	CZ	0.495604	0.152184	a
Cholestenol Delta-isomerase	DZ	0.643914	0.128197	a
Cholestenol Delta-isomerase	EZ	0.719553	0.213712	a
Cholesterol 7-alpha-monooxygenase	AZ	-1.02054	0.14795	b
Cholesterol 7-alpha-monooxygenase	BZ	-0.83223	0.120198	b
Cholesterol 7-alpha-monooxygenase	CZ	0.606055	0.082194	a
Cholesterol 7-alpha-monooxygenase	DZ	0.703955	0.086681	a
Cholesterol 7-alpha-monooxygenase	EZ	0.54276	0.211698	a
Cholesterol oxidase	AZ	-0.89549	0.159783	b
Cholesterol oxidase	BZ	-0.88846	0.09424	b
Cholesterol oxidase	CZ	0.492881	0.09437	a
Cholesterol oxidase	DZ	0.811684	0.080128	a
Cholesterol oxidase	EZ	0.479384	0.232638	a
Choline dehydrogenase	AZ	-0.94408	0.162731	b
Choline dehydrogenase	BZ	-0.89216	0.089786	b
Choline dehydrogenase	CZ	0.5176	0.102129	a
Choline dehydrogenase	DZ	0.865357	0.042888	a
Choline dehydrogenase	EZ	0.453281	0.218437	a
Choline kinase	AZ	-0.91248	0.15591	b
Choline kinase	BZ	-0.91034	0.096844	b
Choline kinase	CZ	0.532948	0.099417	a
Choline kinase	DZ	0.813646	0.063027	a
Choline kinase	EZ	0.476229	0.225272	a
Choline-phosphate cytidylyltransferase	AZ	0.93056	0.095447	a
Choline-phosphate cytidylyltransferase	BZ	0.909451	0.059675	a
Choline-phosphate cytidylyltransferase	CZ	-0.46484	0.199895	b
Choline-phosphate cytidylyltransferase	DZ	-0.82806	0.066387	b
Choline-phosphate cytidylyltransferase	EZ	-0.54711	0.195594	b
Choline-sulfatase	AZ	-0.91645	0.118267	b
Choline-sulfatase	BZ	-0.9706	0.144903	b
Choline-sulfatase	CZ	0.629655	0.111699	a
Choline-sulfatase	DZ	0.674037	0.077536	a
Choline-sulfatase	EZ	0.583354	0.194618	a
Cholinesterase	AZ	-0.87033	0.15858	b

Cholinesterase	BZ	-0.91225	0.102927	b
Cholinesterase	CZ	0.565005	0.109256	a
Cholinesterase	DZ	0.75008	0.06576	a
Cholinesterase	EZ	0.467494	0.231287	a
Chorismate mutase	AZ	0.866703	0.10302	a
Chorismate mutase	BZ	0.962936	0.033329	a
Chorismate mutase	CZ	-0.3878	0.173891	b
Chorismate mutase	DZ	-0.76341	0.109285	b
Chorismate mutase	EZ	-0.67843	0.206158	b
Cinnamoyl-CoA reductase	AZ	-0.56855	0.054568	bc
Cinnamoyl-CoA reductase	BZ	-0.98743	0.147652	c
Cinnamoyl-CoA reductase	CZ	0.558435	0.029317	a
Cinnamoyl-CoA reductase	DZ	0.079358	0.257277	ab
Cinnamoyl-CoA reductase	EZ	0.918183	0.158736	a
Cinnamyl-alcohol dehydrogenase	AZ	-0.94376	0.155571	b
Cinnamyl-alcohol dehydrogenase	BZ	-0.85215	0.101507	b
Cinnamyl-alcohol dehydrogenase	CZ	0.520724	0.125177	a
Cinnamyl-alcohol dehydrogenase	DZ	0.818214	0.034028	a
Cinnamyl-alcohol dehydrogenase	EZ	0.456964	0.224395	a
Citrate (Si)-synthase	AZ	0.217412	0.090619	a
Citrate (Si)-synthase	BZ	0.449131	0.07415	a
Citrate (Si)-synthase	CZ	-0.30174	0.150367	ab
Citrate (Si)-synthase	DZ	0.739167	0.168119	a
Citrate (Si)-synthase	EZ	-1.10397	0.264874	b
Cob(I)yrinic acid a,c-diamide adenosyltransferase	AZ	0.835083	0.040204	a
Cob(I)yrinic acid a,c-diamide adenosyltransferase	BZ	0.883621	0.010458	a
Cob(I)yrinic acid a,c-diamide adenosyltransferase	CZ	-0.14654	0.161629	b
Cob(I)yrinic acid a,c-diamide adenosyltransferase	DZ	-0.73186	0.183458	b
Cob(I)yrinic acid a,c-diamide adenosyltransferase	EZ	-0.8403	0.203534	b
Coproporphyrinogen oxidase	AZ	0.880109	0.086397	a
Coproporphyrinogen oxidase	BZ	0.947023	0.035047	a
Coproporphyrinogen oxidase	CZ	-0.49326	0.185339	b
Coproporphyrinogen oxidase	DZ	-0.52518	0.079381	b
Coproporphyrinogen oxidase	EZ	-0.80869	0.220064	b
CTP synthase (glutamine hydrolyzing)	AZ	0.816514	0.113618	a
CTP synthase (glutamine hydrolyzing)	BZ	0.921202	0.037162	a
CTP synthase (glutamine hydrolyzing)	CZ	-0.16432	0.177174	b
CTP synthase (glutamine hydrolyzing)	DZ	-0.91483	0.113615	b
CTP synthase (glutamine hydrolyzing)	EZ	-0.65857	0.201901	b
Cutinase	AZ	-0.89306	0.079973	b
Cutinase	BZ	-1.12633	0.080446	b
Cutinase	CZ	0.721755	0.103592	a
Cutinase	DZ	0.653864	0.080481	a
Cutinase	EZ	0.643763	0.19614	a

Cyanide hydratase	AZ	-0.90039	0.084789	b
Cyanide hydratase	BZ	-1.14336	0.063779	b
Cyanide hydratase	CZ	0.722926	0.096902	a
Cyanide hydratase	DZ	0.622192	0.085317	a
Cyanide hydratase	EZ	0.698638	0.19048	a
Cyclohexanone monooxygenase	AZ	-0.89967	0.161034	b
Cyclohexanone monooxygenase	BZ	-0.92166	0.098523	b
Cyclohexanone monooxygenase	CZ	0.619735	0.102768	a
Cyclohexanone monooxygenase	DZ	0.728019	0.064119	a
Cyclohexanone monooxygenase	EZ	0.473579	0.22322	a
Cyclopropane-fatty-acyl-phospholipid synthase	AZ	0.858167	0.0778	a
Cyclopropane-fatty-acyl-phospholipid synthase	BZ	0.804272	0.050932	a
Cyclopropane-fatty-acyl-phospholipid synthase	CZ	-0.47257	0.181795	b
Cyclopropane-fatty-acyl-phospholipid synthase	DZ	-0.43083	0.191204	b
Cyclopropane-fatty-acyl-phospholipid synthase	EZ	-0.75904	0.208148	b
Cystathionine beta-lyase	AZ	0.696846	0.086473	a
Cystathionine beta-lyase	BZ	0.804433	0.020012	a
Cystathionine beta-lyase	CZ	-0.5978	0.323419	b
Cystathionine beta-lyase	DZ	-0.63268	0.098814	b
Cystathionine beta-lyase	EZ	-0.2708	0.139907	ab
Cystathionine beta-synthase	AZ	0.873658	0.088349	a
Cystathionine beta-synthase	BZ	0.966711	0.025594	a
Cystathionine beta-synthase	CZ	-0.32623	0.161801	b
Cystathionine beta-synthase	DZ	-0.78466	0.125411	b
Cystathionine beta-synthase	EZ	-0.72948	0.205009	b
Cystathionine gamma-lyase	AZ	0.742417	0.098498	a
Cystathionine gamma-lyase	BZ	0.98223	0.032115	a
Cystathionine gamma-lyase	CZ	-0.31907	0.168159	b
Cystathionine gamma-lyase	DZ	-0.63508	0.16951	b
Cystathionine gamma-lyase	EZ	-0.7705	0.203759	b
Cysteine--tRNA ligase	AZ	0.743752	0.09879	a
Cysteine--tRNA ligase	BZ	0.982104	0.032525	a
Cysteine--tRNA ligase	CZ	-0.31201	0.168066	b
Cysteine--tRNA ligase	DZ	-0.64823	0.170728	b
Cysteine--tRNA ligase	EZ	-0.76561	0.201787	b
Cytidine deaminase	AZ	0.713748	0.108714	a
Cytidine deaminase	BZ	0.974077	0.035077	a
Cytidine deaminase	CZ	-0.42833	0.183618	b
Cytidine deaminase	DZ	-0.6972	0.191323	b
Cytidine deaminase	EZ	-0.56229	0.185556	b
Cytochrome-c oxidase	AZ	0.873297	0.087413	a
Cytochrome-c oxidase	BZ	0.954915	0.026801	a
Cytochrome-c oxidase	CZ	-0.4945	0.190394	b
Cytochrome-c oxidase	DZ	-0.67086	0.11716	b

Cytochrome-c oxidase	EZ	-0.66285	0.203079	b
Cytochrome-c peroxidase	AZ	0.831567	0.084125	a
Cytochrome-c peroxidase	BZ	0.953658	0.025063	a
Cytochrome-c peroxidase	CZ	-0.19722	0.14718	b
Cytochrome-c peroxidase	DZ	-0.96226	0.165811	b
Cytochrome-c peroxidase	EZ	-0.62575	0.187807	b
Cytosine deaminase	AZ	0.687586	0.086645	a
Cytosine deaminase	BZ	0.890953	0.017799	a
Cytosine deaminase	CZ	-0.55942	0.254785	b
Cytosine deaminase	DZ	-0.47207	0.178363	b
Cytosine deaminase	EZ	-0.54705	0.174257	b
D-amino-acid oxidase	AZ	-0.92004	0.094401	b
D-amino-acid oxidase	BZ	-1.06094	0.100953	b
D-amino-acid oxidase	CZ	0.683861	0.106409	a
D-amino-acid oxidase	DZ	0.67866	0.082884	a
D-amino-acid oxidase	EZ	0.618459	0.196377	a
D-arabinono-1,4-lactone oxidase	AZ	-0.93387	0.12289	b
D-arabinono-1,4-lactone oxidase	BZ	-0.88461	0.178934	b
D-arabinono-1,4-lactone oxidase	CZ	0.622037	0.115551	a
D-arabinono-1,4-lactone oxidase	DZ	0.66427	0.075051	a
D-arabinono-1,4-lactone oxidase	EZ	0.53218	0.188661	a
D-arabinose 1-dehydrogenase (NAD(+))	AZ	-0.90841	0.123826	b
D-arabinose 1-dehydrogenase (NAD(+))	BZ	-0.94353	0.150452	b
D-arabinose 1-dehydrogenase (NAD(+))	CZ	0.638575	0.123957	a
D-arabinose 1-dehydrogenase (NAD(+))	DZ	0.709598	0.079418	a
D-arabinose 1-dehydrogenase (NAD(+))	EZ	0.503772	0.190606	a
D-aspartate oxidase	AZ	-0.84023	0.120951	b
D-aspartate oxidase	BZ	-0.92817	0.047503	b
D-aspartate oxidase	CZ	0.434708	0.206741	a
D-aspartate oxidase	DZ	0.854441	0.087746	a
D-aspartate oxidase	EZ	0.479252	0.191996	a
D-lactate dehydrogenase	AZ	-0.99278	0.146119	b
D-lactate dehydrogenase	BZ	-0.85781	0.078079	b
D-lactate dehydrogenase	CZ	0.549636	0.096037	a
D-lactate dehydrogenase	DZ	0.88534	0.068949	a
D-lactate dehydrogenase	EZ	0.415616	0.222643	a
D-lactate dehydrogenase (cytochrome)	AZ	-0.9378	0.150298	b
D-lactate dehydrogenase (cytochrome)	BZ	-0.94063	0.09748	b
D-lactate dehydrogenase (cytochrome)	CZ	0.657035	0.100277	a
D-lactate dehydrogenase (cytochrome)	DZ	0.779019	0.061728	a
D-lactate dehydrogenase (cytochrome)	EZ	0.44237	0.210041	a
D-ribulokinase	AZ	-0.93462	0.157391	b
D-ribulokinase	BZ	-0.89926	0.10283	b
D-ribulokinase	CZ	0.565438	0.103747	a

D-ribulokinase	DZ	0.790598	0.055103	a
D-ribulokinase	EZ	0.477848	0.219293	a
D-stereospecific aminopeptidase	AZ	-0.9166	0.175526	b
D-stereospecific aminopeptidase	BZ	-0.83568	0.103661	b
D-stereospecific aminopeptidase	CZ	0.56724	0.108736	a
D-stereospecific aminopeptidase	DZ	0.711244	0.068499	a
D-stereospecific aminopeptidase	EZ	0.473795	0.228907	a
D-xylose 1-dehydrogenase (NADP(+))	AZ	-0.92589	0.163536	b
D-xylose 1-dehydrogenase (NADP(+))	BZ	-0.87969	0.092501	b
D-xylose 1-dehydrogenase (NADP(+))	CZ	0.442281	0.098137	a
D-xylose 1-dehydrogenase (NADP(+))	DZ	0.868375	0.048791	a
D-xylose 1-dehydrogenase (NADP(+))	EZ	0.494924	0.226716	a
D-xylulose reductase	AZ	-0.62131	0.162597	b
D-xylulose reductase	BZ	-0.98693	0.176339	b
D-xylulose reductase	CZ	0.535891	0.127282	a
D-xylulose reductase	DZ	0.552035	0.093545	a
D-xylulose reductase	EZ	0.520311	0.215361	a
dCMP deaminase	AZ	0.865906	0.10123	a
dCMP deaminase	BZ	0.96052	0.032751	a
dCMP deaminase	CZ	-0.3651	0.170496	b
dCMP deaminase	DZ	-0.75641	0.111295	b
dCMP deaminase	EZ	-0.70491	0.208535	b
Deoxyhypusine monooxygenase	AZ	-0.90841	0.123829	b
Deoxyhypusine monooxygenase	BZ	-0.94353	0.150456	b
Deoxyhypusine monooxygenase	CZ	0.638615	0.12396	a
Deoxyhypusine monooxygenase	DZ	0.709639	0.07942	a
Deoxyhypusine monooxygenase	EZ	0.50368	0.190599	a
Deoxyribodipyrimidine photo-lyase	AZ	-0.83818	0.165499	b
Deoxyribodipyrimidine photo-lyase	BZ	-0.93859	0.11621	b
Deoxyribodipyrimidine photo-lyase	CZ	0.594367	0.112502	a
Deoxyribodipyrimidine photo-lyase	DZ	0.724439	0.056826	a
Deoxyribodipyrimidine photo-lyase	EZ	0.45797	0.22283	a
Deoxyribose-phosphate aldolase	AZ	-0.97741	0.155763	b
Deoxyribose-phosphate aldolase	BZ	-0.83236	0.096358	b
Deoxyribose-phosphate aldolase	CZ	0.537159	0.104918	a
Deoxyribose-phosphate aldolase	DZ	0.785127	0.06005	a
Deoxyribose-phosphate aldolase	EZ	0.48748	0.228695	a
Dephospho-CoA kinase	AZ	0.824091	0.101715	a
Dephospho-CoA kinase	BZ	0.931302	0.028927	a
Dephospho-CoA kinase	CZ	-0.58988	0.229794	b
Dephospho-CoA kinase	DZ	-0.58552	0.10611	b
Dephospho-CoA kinase	EZ	-0.57999	0.189924	b
Diacylglycerol O-acyltransferase	AZ	-0.87845	0.163223	b
Diacylglycerol O-acyltransferase	BZ	-0.87843	0.09759	b

Diacylglycerol O-acyltransferase	CZ	0.472243	0.094429	a
Diacylglycerol O-acyltransferase	DZ	0.776273	0.096372	a
Diacylglycerol O-acyltransferase	EZ	0.50836	0.232943	a
Diamine N-acetyltransferase	AZ	0.863384	0.094355	a
Diamine N-acetyltransferase	BZ	0.894808	0.043418	a
Diamine N-acetyltransferase	CZ	-0.08535	0.155745	b
Diamine N-acetyltransferase	DZ	-0.90812	0.100018	b
Diamine N-acetyltransferase	EZ	-0.76472	0.216352	b
Diaminopropionate ammonia-lyase	AZ	-0.72824	0.069858	b
Diaminopropionate ammonia-lyase	BZ	-0.77055	0.071882	b
Diaminopropionate ammonia-lyase	CZ	0.617972	0.033574	a
Diaminopropionate ammonia-lyase	DZ	-0.03072	0.280921	ab
Diaminopropionate ammonia-lyase	EZ	0.911544	0.181706	a
Dihydrofolate reductase	AZ	0.655807	0.132667	a
Dihydrofolate reductase	BZ	0.889341	0.022859	a
Dihydrofolate reductase	CZ	-0.22307	0.214459	ab
Dihydrofolate reductase	DZ	-0.82999	0.173291	b
Dihydrofolate reductase	EZ	-0.49209	0.189115	b
Dihydrolipoyl dehydrogenase	AZ	0.815499	0.106689	a
Dihydrolipoyl dehydrogenase	BZ	0.84664	0.045883	a
Dihydrolipoyl dehydrogenase	CZ	-0.20016	0.208602	b
Dihydrolipoyl dehydrogenase	DZ	-0.91823	0.107935	b
Dihydrolipoyl dehydrogenase	EZ	-0.54375	0.208115	b
Dihydrolipoyllysine-residue acetyltransferase	AZ	0.883488	0.081585	a
Dihydrolipoyllysine-residue acetyltransferase	BZ	0.933791	0.035267	a
Dihydrolipoyllysine-residue acetyltransferase	CZ	-0.35844	0.186738	b
Dihydrolipoyllysine-residue acetyltransferase	DZ	-0.8233	0.110406	b
Dihydrolipoyllysine-residue acetyltransferase	EZ	-0.63554	0.204703	b
Dihydrolipoyllysine-residue succinyltransferase	AZ	0.864777	0.102822	a
Dihydrolipoyllysine-residue succinyltransferase	BZ	0.90651	0.038662	a
Dihydrolipoyllysine-residue succinyltransferase	CZ	-0.15344	0.169198	b
Dihydrolipoyllysine-residue succinyltransferase	DZ	-0.96345	0.09462	b
Dihydrolipoyllysine-residue succinyltransferase	EZ	-0.6544	0.207053	b
Dihydroorotase	AZ	0.866802	0.103111	a
Dihydroorotase	BZ	0.962972	0.033353	a
Dihydroorotase	CZ	-0.38555	0.173323	b
Dihydroorotase	DZ	-0.76463	0.109365	b
Dihydroorotase	EZ	-0.67959	0.206309	b
Dihydropyrimidinase	AZ	-0.73859	0.163846	b
Dihydropyrimidinase	BZ	-0.92158	0.130386	b
Dihydropyrimidinase	CZ	0.460932	0.078836	a
Dihydropyrimidinase	DZ	0.532382	0.098237	a
Dihydropyrimidinase	EZ	0.666853	0.252028	a
Dihydroxy-acid dehydratase	AZ	-0.87292	0.126309	b

Dihydroxy-acid dehydratase	BZ	-0.96763	0.05593	b
Dihydroxy-acid dehydratase	CZ	0.418869	0.147337	a
Dihydroxy-acid dehydratase	DZ	0.910924	0.100522	a
Dihydroxy-acid dehydratase	EZ	0.510749	0.202202	a
Dipeptidyl-peptidase III	AZ	-0.96183	0.159487	b
Dipeptidyl-peptidase III	BZ	-0.8503	0.096049	b
Dipeptidyl-peptidase III	CZ	0.491324	0.101648	a
Dipeptidyl-peptidase III	DZ	0.853622	0.049901	a
Dipeptidyl-peptidase III	EZ	0.467181	0.225153	a
Dipeptidyl-peptidase IV	AZ	-1.05097	0.177448	b
Dipeptidyl-peptidase IV	BZ	-0.44373	0.146043	ab
Dipeptidyl-peptidase IV	CZ	0.628667	0.190386	a
Dipeptidyl-peptidase IV	DZ	0.601597	0.086984	a
Dipeptidyl-peptidase IV	EZ	0.264441	0.19221	a
diphosphate specific)	AZ	-0.97262	0.164095	b
diphosphate specific)	BZ	-0.81743	0.0965	b
diphosphate specific)	CZ	0.519702	0.1163	a
diphosphate specific)	DZ	0.851976	0.037579	a
diphosphate specific)	EZ	0.418365	0.222632	a
Diphosphomevalonate decarboxylase	AZ	0.866802	0.103111	a
Diphosphomevalonate decarboxylase	BZ	0.962972	0.033353	a
Diphosphomevalonate decarboxylase	CZ	-0.38555	0.173323	b
Diphosphomevalonate decarboxylase	DZ	-0.76463	0.109365	b
Diphosphomevalonate decarboxylase	EZ	-0.67959	0.206309	b
Diphthine synthase	AZ	0.866659	0.102881	a
Diphthine synthase	BZ	0.962763	0.033284	a
Diphthine synthase	CZ	-0.38295	0.172962	b
Diphthine synthase	DZ	-0.76987	0.110636	b
Diphthine synthase	EZ	-0.6766	0.205898	b
DNA (cytosine-5-)-methyltransferase	AZ	0.877879	0.076274	a
DNA (cytosine-5-)-methyltransferase	BZ	0.954228	0.023	a
DNA (cytosine-5-)-methyltransferase	CZ	-0.42481	0.178044	b
DNA (cytosine-5-)-methyltransferase	DZ	-0.72756	0.141883	b
DNA (cytosine-5-)-methyltransferase	EZ	-0.67974	0.197912	b
DNA ligase (ATP)	AZ	0.879506	0.088817	a
DNA ligase (ATP)	BZ	0.978186	0.025682	a
DNA ligase (ATP)	CZ	-0.47375	0.182827	b
DNA ligase (ATP)	DZ	-0.73537	0.11385	b
DNA ligase (ATP)	EZ	-0.64858	0.198123	b
DNA ligase (NAD(+))	AZ	0.835046	0.086852	a
DNA ligase (NAD(+))	BZ	0.92939	0.024518	a
DNA ligase (NAD(+))	CZ	-0.57938	0.190124	b
DNA ligase (NAD(+))	DZ	-0.80888	0.144687	b
DNA ligase (NAD(+))	EZ	-0.37618	0.200367	b

DNA topoisomerase	AZ	0.806338	0.113358	a
DNA topoisomerase	BZ	0.948682	0.030708	a
DNA topoisomerase	CZ	-0.32984	0.152494	b
DNA topoisomerase	DZ	-0.77436	0.169032	b
DNA topoisomerase	EZ	-0.65082	0.200678	b
DNA topoisomerase (ATP-hydrolyzing)	AZ	0.878896	0.103108	a
DNA topoisomerase (ATP-hydrolyzing)	BZ	0.968019	0.031129	a
DNA topoisomerase (ATP-hydrolyzing)	CZ	-0.39192	0.169772	b
DNA topoisomerase (ATP-hydrolyzing)	DZ	-0.83579	0.109078	b
DNA topoisomerase (ATP-hydrolyzing)	EZ	-0.6192	0.20106	b
DNA-(apurinic or apyrimidinic site) lyase	AZ	0.828237	0.110532	a
DNA-(apurinic or apyrimidinic site) lyase	BZ	0.863172	0.054667	a
DNA-(apurinic or apyrimidinic site) lyase	CZ	-0.52743	0.194249	b
DNA-(apurinic or apyrimidinic site) lyase	DZ	-0.53939	0.16924	b
DNA-(apurinic or apyrimidinic site) lyase	EZ	-0.62459	0.197244	b
DNA-3-methyladenine glycosylase II	AZ	-0.85219	0.152833	b
DNA-3-methyladenine glycosylase II	BZ	-0.99649	0.108609	b
DNA-3-methyladenine glycosylase II	CZ	0.569026	0.099645	a
DNA-3-methyladenine glycosylase II	DZ	0.793291	0.063493	a
DNA-3-methyladenine glycosylase II	EZ	0.486357	0.213174	a
DNA-directed DNA polymerase	AZ	0.895643	0.091354	a
DNA-directed DNA polymerase	BZ	0.97424	0.029609	a
DNA-directed DNA polymerase	CZ	-0.41314	0.169515	b
DNA-directed DNA polymerase	DZ	-0.78301	0.105506	b
DNA-directed DNA polymerase	EZ	-0.67373	0.203616	b
DNA-directed RNA polymerase	AZ	0.862055	0.097365	a
DNA-directed RNA polymerase	BZ	0.965308	0.031637	a
DNA-directed RNA polymerase	CZ	-0.45383	0.175629	b
DNA-directed RNA polymerase	DZ	-0.7051	0.12486	b
DNA-directed RNA polymerase	EZ	-0.66842	0.204547	b
Dodecenoyl-CoA isomerase	AZ	0.813185	0.10765	a
Dodecenoyl-CoA isomerase	BZ	0.892638	0.034714	a
Dodecenoyl-CoA isomerase	CZ	-0.20083	0.144657	b
Dodecenoyl-CoA isomerase	DZ	-0.90828	0.168754	b
Dodecenoyl-CoA isomerase	EZ	-0.59671	0.209358	b
Dolichyl-phosphate beta-D-mannosyltransferase	AZ	0.861107	0.107153	a
Dolichyl-phosphate beta-D-mannosyltransferase	BZ	0.961047	0.034661	a
Dolichyl-phosphate beta-D-mannosyltransferase	CZ	-0.43803	0.179481	b
Dolichyl-phosphate beta-D-mannosyltransferase	DZ	-0.77577	0.122213	b
Dolichyl-phosphate beta-D-mannosyltransferase	EZ	-0.60835	0.19678	b
Dolichyl-phosphate beta-glucosyltransferase	AZ	0.867047	0.10304	a
Dolichyl-phosphate beta-glucosyltransferase	BZ	0.962425	0.033191	a
Dolichyl-phosphate beta-glucosyltransferase	CZ	-0.3877	0.173925	b
Dolichyl-phosphate beta-glucosyltransferase	DZ	-0.76338	0.109307	b

Dolichyl-phosphate beta-glucosyltransferase	EZ	-0.67839	0.206199	b
Dolichyl-phosphate-mannose--protein mannosyltransferase	AZ	0.877333	0.094757	a
Dolichyl-phosphate-mannose--protein mannosyltransferase	BZ	0.980731	0.02674	a
Dolichyl-phosphate-mannose--protein mannosyltransferase	CZ	-0.39051	0.162942	b
Dolichyl-phosphate-mannose--protein mannosyltransferase	DZ	-0.77457	0.118107	b
Dolichyl-phosphate-mannose--protein mannosyltransferase	EZ	-0.69298	0.203949	b
Dolichyldiphosphatase	AZ	0.867368	0.100021	a
Dolichyldiphosphatase	BZ	0.958979	0.032277	a
Dolichyldiphosphatase	CZ	-0.35531	0.16965	b
Dolichyldiphosphatase	DZ	-0.76218	0.111714	b
Dolichyldiphosphatase	EZ	-0.70886	0.208836	b
dTDP-4-dehydrorhamnose reductase	AZ	-0.90825	0.123915	b
dTDP-4-dehydrorhamnose reductase	BZ	-0.9434	0.150561	b
dTDP-4-dehydrorhamnose reductase	CZ	0.639849	0.124046	a
dTDP-4-dehydrorhamnose reductase	DZ	0.709765	0.079869	a
dTDP-4-dehydrorhamnose reductase	EZ	0.502036	0.190272	a
dTDP-glucose 4,6-dehydratase	AZ	0.845853	0.04726	a
dTDP-glucose 4,6-dehydratase	BZ	0.817423	0.027576	a
dTDP-glucose 4,6-dehydratase	CZ	-0.49577	0.234614	b
dTDP-glucose 4,6-dehydratase	DZ	-0.5134	0.176644	b
dTDP-glucose 4,6-dehydratase	EZ	-0.65411	0.186322	b
dTMP kinase	AZ	0.869003	0.101853	a
dTMP kinase	BZ	0.961099	0.033099	a
dTMP kinase	CZ	-0.37196	0.17163	b
dTMP kinase	DZ	-0.78328	0.111643	b
dTMP kinase	EZ	-0.67486	0.205669	b
dUTP diphosphatase	AZ	0.808314	0.125982	a
dUTP diphosphatase	BZ	0.915774	0.041947	a
dUTP diphosphatase	CZ	-0.22114	0.174174	b
dUTP diphosphatase	DZ	-0.84952	0.125499	b
dUTP diphosphatase	EZ	-0.65343	0.20446	b
Electron-transferring-flavoprotein dehydrogenase	AZ	0.866802	0.103111	a
Electron-transferring-flavoprotein dehydrogenase	BZ	0.962972	0.033353	a
Electron-transferring-flavoprotein dehydrogenase	CZ	-0.38555	0.173323	b
Electron-transferring-flavoprotein dehydrogenase	DZ	-0.76463	0.109365	b
Electron-transferring-flavoprotein dehydrogenase	EZ	-0.67959	0.206309	b
Endo-1,3(4)-beta-glucanase	AZ	-0.8757	0.099626	b
Endo-1,3(4)-beta-glucanase	BZ	-1.09717	0.084218	b
Endo-1,3(4)-beta-glucanase	CZ	0.693528	0.120397	a
Endo-1,3(4)-beta-glucanase	DZ	0.736839	0.08846	a
Endo-1,3(4)-beta-glucanase	EZ	0.542509	0.19031	a
Endo-1,4-beta-xylanase	AZ	-0.86866	0.078365	b
Endo-1,4-beta-xylanase	BZ	-1.16082	0.069909	b
Endo-1,4-beta-xylanase	CZ	0.718428	0.109295	a

Endo-1,4-beta-xylanase	DZ	0.669971	0.088133	a
Endo-1,4-beta-xylanase	EZ	0.641081	0.188031	a
Endopeptidase Clp	AZ	0.878363	0.1008	a
Endopeptidase Clp	BZ	0.939057	0.039103	a
Endopeptidase Clp	CZ	-0.33163	0.168665	b
Endopeptidase Clp	DZ	-0.77716	0.109325	b
Endopeptidase Clp	EZ	-0.70864	0.210801	b
Endopeptidase La	AZ	0.88085	0.104085	a
Endopeptidase La	BZ	0.944358	0.040482	a
Endopeptidase La	CZ	-0.37057	0.173447	b
Endopeptidase La	DZ	-0.77217	0.102079	b
Endopeptidase La	EZ	-0.68247	0.208978	b
Endothelin-converting enzyme 1	AZ	0.835601	0.093681	a
Endothelin-converting enzyme 1	BZ	0.962459	0.026649	a
Endothelin-converting enzyme 1	CZ	-0.27264	0.153659	b
Endothelin-converting enzyme 1	DZ	-0.71203	0.119706	b
Endothelin-converting enzyme 1	EZ	-0.81339	0.221809	b
Enoyl-[acyl-carrier-protein] reductase (NADH)	AZ	-0.91871	0.120513	b
Enoyl-[acyl-carrier-protein] reductase (NADH)	BZ	-0.95718	0.147102	b
Enoyl-[acyl-carrier-protein] reductase (NADH)	CZ	0.638484	0.113559	a
Enoyl-[acyl-carrier-protein] reductase (NADH)	DZ	0.680863	0.076623	a
Enoyl-[acyl-carrier-protein] reductase (NADH)	EZ	0.55654	0.194965	a
Enoyl-CoA hydratase	AZ	-0.79318	0.121107	b
Enoyl-CoA hydratase	BZ	-0.93122	0.041263	b
Enoyl-CoA hydratase	CZ	0.570856	0.216209	a
Enoyl-CoA hydratase	DZ	0.531982	0.11245	a
Enoyl-CoA hydratase	EZ	0.62156	0.199156	a
Ethanolamine kinase	AZ	0.839879	0.060378	a
Ethanolamine kinase	BZ	0.911535	0.015855	a
Ethanolamine kinase	CZ	-0.14484	0.167494	b
Ethanolamine kinase	DZ	-0.79322	0.144907	b
Ethanolamine kinase	EZ	-0.81335	0.211306	b
Ethanolamine-phosphate cytidylyltransferase	AZ	0.815717	0.0599	a
Ethanolamine-phosphate cytidylyltransferase	BZ	0.910086	0.012595	a
Ethanolamine-phosphate cytidylyltransferase	CZ	-0.12323	0.161185	b
Ethanolamine-phosphate cytidylyltransferase	DZ	-0.82455	0.168466	b
Ethanolamine-phosphate cytidylyltransferase	EZ	-0.77802	0.205401	b
Ethanolaminephosphotransferase	AZ	0.873465	0.096645	a
Ethanolaminephosphotransferase	BZ	0.958418	0.029897	a
Ethanolaminephosphotransferase	CZ	-0.53098	0.205701	b
Ethanolaminephosphotransferase	DZ	-0.72306	0.100966	b
Ethanolaminephosphotransferase	EZ	-0.57784	0.190396	b
Exodeoxyribonuclease I	AZ	-0.81427	0.150466	b
Exodeoxyribonuclease I	BZ	-0.93303	0.053071	b

Exodeoxyribonuclease I	CZ	0.396306	0.174563	a
Exodeoxyribonuclease I	DZ	0.773478	0.092986	a
Exodeoxyribonuclease I	EZ	0.57751	0.210486	a
Exodeoxyribonuclease III	AZ	-0.90714	0.128328	b
Exodeoxyribonuclease III	BZ	-0.9203	0.166701	b
Exodeoxyribonuclease III	CZ	0.604986	0.122648	a
Exodeoxyribonuclease III	DZ	0.730794	0.07671	a
Exodeoxyribonuclease III	EZ	0.491656	0.185024	a
Exopolyphosphatase	AZ	0.852634	0.108233	a
Exopolyphosphatase	BZ	0.956631	0.035052	a
Exopolyphosphatase	CZ	-0.43332	0.178934	b
Exopolyphosphatase	DZ	-0.71809	0.122164	b
Exopolyphosphatase	EZ	-0.65785	0.202977	b
Exoribonuclease II	AZ	0.846506	0.075677	a
Exoribonuclease II	BZ	0.911938	0.027105	a
Exoribonuclease II	CZ	-0.40036	0.166708	b
Exoribonuclease II	DZ	-0.79575	0.140891	b
Exoribonuclease II	EZ	-0.56233	0.230217	b
Factor independent urate hydroxylase	AZ	-0.92404	0.15385	b
Factor independent urate hydroxylase	BZ	-0.91082	0.10043	b
Factor independent urate hydroxylase	CZ	0.543322	0.107363	a
Factor independent urate hydroxylase	DZ	0.838247	0.049594	a
Factor independent urate hydroxylase	EZ	0.453288	0.218905	a
FAD synthetase	AZ	0.795408	0.129881	a
FAD synthetase	BZ	0.944773	0.051275	a
FAD synthetase	CZ	-0.21661	0.161352	b
FAD synthetase	DZ	-0.84861	0.113737	b
FAD synthetase	EZ	-0.67496	0.210085	b
Farnesol dehydrogenase	AZ	-0.56988	0.054405	bc
Farnesol dehydrogenase	BZ	-0.9875	0.147211	c
Farnesol dehydrogenase	CZ	0.553739	0.02923	a
Farnesol dehydrogenase	DZ	0.081259	0.25686	ab
Farnesol dehydrogenase	EZ	0.922386	0.159186	a
Fatty-acid synthase	AZ	-0.92131	0.126391	b
Fatty-acid synthase	BZ	-0.98216	0.08409	b
Fatty-acid synthase	CZ	0.642496	0.102338	a
Fatty-acid synthase	DZ	0.70546	0.078685	a
Fatty-acid synthase	EZ	0.555513	0.220599	a
Fatty-acyl-CoA synthase	AZ	-0.90969	0.115267	b
Fatty-acyl-CoA synthase	BZ	-0.95414	0.141032	b
Fatty-acyl-CoA synthase	CZ	0.685066	0.13735	a
Fatty-acyl-CoA synthase	DZ	0.723194	0.085305	a
Fatty-acyl-CoA synthase	EZ	0.455572	0.183825	a
Ferredoxin--NADP(+) reductase	AZ	0.819251	0.088611	a

Ferredoxin--NADP(+) reductase	BZ	0.898189	0.026884	a
Ferredoxin--NADP(+) reductase	CZ	-0.36224	0.189937	b
Ferredoxin--NADP(+) reductase	DZ	-0.8341	0.177618	b
Ferredoxin--NADP(+) reductase	EZ	-0.5211	0.187033	b
Ferrochelatase	AZ	0.80222	0.142181	a
Ferrochelatase	BZ	0.915018	0.049803	a
Ferrochelatase	CZ	-0.19186	0.163065	b
Ferrochelatase	DZ	-0.85798	0.103863	b
Ferrochelatase	EZ	-0.66739	0.212056	b
Ferroxidase	AZ	-0.90952	0.123718	b
Ferroxidase	BZ	-0.94461	0.150321	b
Ferroxidase	CZ	0.645753	0.123083	a
Ferroxidase	DZ	0.707076	0.079349	a
Ferroxidase	EZ	0.501301	0.190429	a
Feruloyl esterase	AZ	-0.93148	0.110323	b
Feruloyl esterase	BZ	-0.97411	0.146873	b
Feruloyl esterase	CZ	0.640422	0.098296	a
Feruloyl esterase	DZ	0.611997	0.070341	a
Feruloyl esterase	EZ	0.653175	0.200633	a
Flavanone 3-dioxygenase	AZ	-0.95673	0.15581	b
Flavanone 3-dioxygenase	BZ	-0.8687	0.090217	b
Flavanone 3-dioxygenase	CZ	0.590792	0.119343	a
Flavanone 3-dioxygenase	DZ	0.804119	0.045373	a
Flavanone 3-dioxygenase	EZ	0.430522	0.220758	a
Flavin-containing monooxygenase	AZ	-0.93897	0.138805	b
Flavin-containing monooxygenase	BZ	-0.95143	0.061363	b
Flavin-containing monooxygenase	CZ	0.520097	0.131968	a
Flavin-containing monooxygenase	DZ	0.814861	0.062894	a
Flavin-containing monooxygenase	EZ	0.55545	0.21	a
Flavonoid 3'-monooxygenase	AZ	-0.86256	0.215038	b
Flavonoid 3'-monooxygenase	BZ	-0.73753	0.07898	b
Flavonoid 3'-monooxygenase	CZ	0.125479	0.104025	ab
Flavonoid 3'-monooxygenase	DZ	0.960877	0.179714	a
Flavonoid 3'-monooxygenase	EZ	0.513735	0.140915	a
Formamidase	AZ	-0.97125	0.157303	b
Formamidase	BZ	-0.87098	0.08543	b
Formamidase	CZ	0.481677	0.095764	a
Formamidase	DZ	0.885375	0.056545	a
Formamidase	EZ	0.475178	0.220018	a
Formate dehydrogenase	AZ	-0.98872	0.163148	b
Formate dehydrogenase	BZ	-0.82584	0.098056	b
Formate dehydrogenase	CZ	0.539672	0.109791	a
Formate dehydrogenase	DZ	0.842627	0.046846	a
Formate dehydrogenase	EZ	0.432262	0.217104	a

Formyltetrahydrofolate deformylase	AZ	-0.89245	0.163891	b
Formyltetrahydrofolate deformylase	BZ	-0.87555	0.103226	b
Formyltetrahydrofolate deformylase	CZ	0.556006	0.094107	a
Formyltetrahydrofolate deformylase	DZ	0.786547	0.078955	a
Formyltetrahydrofolate deformylase	EZ	0.425447	0.231951	a
Fructose-2,6-bisphosphate 2-phosphatase	AZ	-0.75631	0.137009	c
Fructose-2,6-bisphosphate 2-phosphatase	BZ	-0.89133	0.035196	c
Fructose-2,6-bisphosphate 2-phosphatase	CZ	-0.03959	0.140602	bc
Fructose-2,6-bisphosphate 2-phosphatase	DZ	1.033722	0.10827	a
Fructose-2,6-bisphosphate 2-phosphatase	EZ	0.653511	0.212869	ab
Fructose-bisphosphatase	AZ	0.866802	0.103111	a
Fructose-bisphosphatase	BZ	0.962972	0.033353	a
Fructose-bisphosphatase	CZ	-0.38555	0.173323	b
Fructose-bisphosphatase	DZ	-0.76463	0.109365	b
Fructose-bisphosphatase	EZ	-0.67959	0.206309	b
Fructose-bisphosphate aldolase	AZ	0.866714	0.088581	a
Fructose-bisphosphate aldolase	BZ	0.968508	0.023669	a
Fructose-bisphosphate aldolase	CZ	-0.29082	0.167617	b
Fructose-bisphosphate aldolase	DZ	-0.87798	0.121653	b
Fructose-bisphosphate aldolase	EZ	-0.66643	0.198144	b
Fumarate hydratase	AZ	0.866798	0.103114	a
Fumarate hydratase	BZ	0.96297	0.033354	a
Fumarate hydratase	CZ	-0.38558	0.173327	b
Fumarate hydratase	DZ	-0.76467	0.109368	b
Fumarate hydratase	EZ	-0.67951	0.206306	b
Galactinol--sucrose galactosyltransferase	AZ	-0.90064	0.150004	b
Galactinol--sucrose galactosyltransferase	BZ	-0.80898	0.156116	b
Galactinol--sucrose galactosyltransferase	CZ	0.748152	0.116684	a
Galactinol--sucrose galactosyltransferase	DZ	0.408587	0.141122	a
Galactinol--sucrose galactosyltransferase	EZ	0.552882	0.186275	a
Galactokinase	AZ	0.866641	0.102984	a
Galactokinase	BZ	0.962692	0.033312	a
Galactokinase	CZ	-0.38728	0.173973	b
Galactokinase	DZ	-0.76278	0.10923	b
Galactokinase	EZ	-0.67928	0.206236	b
Galactonate dehydratase	AZ	-0.9096	0.155051	b
Galactonate dehydratase	BZ	-0.93544	0.094067	b
Galactonate dehydratase	CZ	0.580966	0.097432	a
Galactonate dehydratase	DZ	0.791781	0.066693	a
Galactonate dehydratase	EZ	0.472287	0.220623	a
Galactose oxidase	AZ	-0.86577	0.081599	b
Galactose oxidase	BZ	-1.18528	0.06358	b
Galactose oxidase	CZ	0.734458	0.099367	a
Galactose oxidase	DZ	0.633332	0.089641	a

Galactose oxidase	EZ	0.683261	0.182478	a
Galacturan 1,4-alpha-galacturonidase	AZ	-0.8738	0.166675	b
Galacturan 1,4-alpha-galacturonidase	BZ	-0.88604	0.106361	b
Galacturan 1,4-alpha-galacturonidase	CZ	0.533786	0.121649	a
Galacturan 1,4-alpha-galacturonidase	DZ	0.794108	0.045592	a
Galacturan 1,4-alpha-galacturonidase	EZ	0.431948	0.226923	a
Gamma-butyrobetaine dioxygenase	AZ	0.915639	0.098987	a
Gamma-butyrobetaine dioxygenase	BZ	0.894311	0.062651	a
Gamma-butyrobetaine dioxygenase	CZ	-0.40948	0.204425	b
Gamma-butyrobetaine dioxygenase	DZ	-0.83573	0.062484	b
Gamma-butyrobetaine dioxygenase	EZ	-0.56473	0.198546	b
Gamma-glutamyltransferase	AZ	0.861891	0.084074	a
Gamma-glutamyltransferase	BZ	0.899508	0.037382	a
Gamma-glutamyltransferase	CZ	-0.43404	0.217634	b
Gamma-glutamyltransferase	DZ	-0.7563	0.126786	b
Gamma-glutamyltransferase	EZ	-0.57106	0.190189	b
Gastricsin	AZ	0.807492	0.061268	a
Gastricsin	BZ	0.857597	0.023432	a
Gastricsin	CZ	-0.38441	0.171767	b
Gastricsin	DZ	-0.29148	0.151981	b
Gastricsin	EZ	-0.9892	0.230557	b
GDP-Man:Man(1)GlcNAc(2)-PP-dolichol alpha-1,3-mannosyltransferase	AZ	0.862866	0.080861	a
GDP-Man:Man(1)GlcNAc(2)-PP-dolichol alpha-1,3-mannosyltransferase	BZ	0.925127	0.028705	a
GDP-Man:Man(1)GlcNAc(2)-PP-dolichol alpha-1,3-mannosyltransferase	CZ	-0.4468	0.205433	b
GDP-Man:Man(1)GlcNAc(2)-PP-dolichol alpha-1,3-mannosyltransferase	DZ	-0.71895	0.120563	b
GDP-Man:Man(1)GlcNAc(2)-PP-dolichol alpha-1,3-mannosyltransferase	EZ	-0.62224	0.201728	b
GDP-Man:Man(3)GlcNAc(2)-PP-dolichol alpha-1,2-mannosyltransferase	AZ	-0.93975	0.159633	b
GDP-Man:Man(3)GlcNAc(2)-PP-dolichol alpha-1,2-mannosyltransferase	BZ	-0.88984	0.09832	b
GDP-Man:Man(3)GlcNAc(2)-PP-dolichol alpha-1,2-mannosyltransferase	CZ	0.514665	0.110285	a
GDP-Man:Man(3)GlcNAc(2)-PP-dolichol alpha-1,2-mannosyltransferase	DZ	0.878008	0.03771	a
GDP-Man:Man(3)GlcNAc(2)-PP-dolichol alpha-1,2-mannosyltransferase	EZ	0.436918	0.214906	a
GDP-mannose 4,6-dehydratase	AZ	0.817677	0.057639	a
GDP-mannose 4,6-dehydratase	BZ	0.882654	0.016472	a
GDP-mannose 4,6-dehydratase	CZ	-0.10255	0.151111	ab
GDP-mannose 4,6-dehydratase	DZ	-0.71348	0.168882	b
GDP-mannose 4,6-dehydratase	EZ	-0.8843	0.218149	b
Gentisate 1,2-dioxygenase	AZ	-0.86261	0.070201	b
Gentisate 1,2-dioxygenase	BZ	-1.18146	0.085393	b
Gentisate 1,2-dioxygenase	CZ	0.760114	0.097335	a
Gentisate 1,2-dioxygenase	DZ	0.59939	0.091215	a
Gentisate 1,2-dioxygenase	EZ	0.684561	0.180847	a
Gibberellin 2-beta-dioxygenase	AZ	-0.93036	0.161832	b
Gibberellin 2-beta-dioxygenase	BZ	-0.93533	0.094238	b
Gibberellin 2-beta-dioxygenase	CZ	0.571708	0.100574	a

Gibberellin 2-beta-dioxygenase	DZ	0.815236	0.04286	a
Gibberellin 2-beta-dioxygenase	EZ	0.478747	0.211952	a
Gibberellin 3-beta-dioxygenase	AZ	-0.72824	0.069862	b
Gibberellin 3-beta-dioxygenase	BZ	-0.77056	0.071886	b
Gibberellin 3-beta-dioxygenase	CZ	0.61804	0.033576	a
Gibberellin 3-beta-dioxygenase	DZ	-0.03068	0.280935	ab
Gibberellin 3-beta-dioxygenase	EZ	0.911445	0.181693	a
Gibberellin-44 dioxygenase	AZ	-0.96755	0.090782	b
Gibberellin-44 dioxygenase	BZ	-0.99187	0.155889	b
Gibberellin-44 dioxygenase	CZ	0.584763	0.076445	a
Gibberellin-44 dioxygenase	DZ	0.609108	0.055558	a
Gibberellin-44 dioxygenase	EZ	0.765554	0.193847	a
Glucan 1,3-alpha-glucosidase	AZ	0.78523	0.121314	a
Glucan 1,3-alpha-glucosidase	BZ	0.912185	0.041996	a
Glucan 1,3-alpha-glucosidase	CZ	-0.23037	0.144654	b
Glucan 1,3-alpha-glucosidase	DZ	-0.70523	0.124307	b
Glucan 1,3-alpha-glucosidase	EZ	-0.76182	0.241184	b
Glucan 1,3-beta-glucosidase	AZ	-0.93853	0.162661	b
Glucan 1,3-beta-glucosidase	BZ	-0.87701	0.104616	b
Glucan 1,3-beta-glucosidase	CZ	0.576822	0.108998	a
Glucan 1,3-beta-glucosidase	DZ	0.76485	0.046522	a
Glucan 1,3-beta-glucosidase	EZ	0.473871	0.221221	a
Glucan endo-1,3-alpha-glucosidase	AZ	-0.95184	0.152322	b
Glucan endo-1,3-alpha-glucosidase	BZ	-0.87213	0.091409	b
Glucan endo-1,3-alpha-glucosidase	CZ	0.565191	0.110217	a
Glucan endo-1,3-alpha-glucosidase	DZ	0.860792	0.049356	a
Glucan endo-1,3-alpha-glucosidase	EZ	0.397989	0.222684	a
Glucan endo-1,3-beta-D-glucosidase	AZ	-0.90787	0.145186	b
Glucan endo-1,3-beta-D-glucosidase	BZ	-0.88086	0.165966	b
Glucan endo-1,3-beta-D-glucosidase	CZ	0.600739	0.120863	a
Glucan endo-1,3-beta-D-glucosidase	DZ	0.715926	0.081683	a
Glucan endo-1,3-beta-D-glucosidase	EZ	0.472067	0.187816	a
Glucomannan 4-beta-mannosyltransferase	AZ	-0.79159	0.103441	b
Glucomannan 4-beta-mannosyltransferase	BZ	-1.15576	0.057951	b
Glucomannan 4-beta-mannosyltransferase	CZ	0.790028	0.133696	a
Glucomannan 4-beta-mannosyltransferase	DZ	0.702436	0.118271	a
Glucomannan 4-beta-mannosyltransferase	EZ	0.454893	0.172436	a
Gluconate 2-dehydrogenase	AZ	-0.91357	0.158928	b
Gluconate 2-dehydrogenase	BZ	-0.87659	0.097108	b
Gluconate 2-dehydrogenase	CZ	0.54477	0.102262	a
Gluconate 2-dehydrogenase	DZ	0.821432	0.078104	a
Gluconate 2-dehydrogenase	EZ	0.423955	0.225921	a
Gluconolactonase	AZ	-0.94246	0.154226	b
Gluconolactonase	BZ	-0.89683	0.10965	b

Gluconolactonase	CZ	0.59767	0.102439	a
Gluconolactonase	DZ	0.758877	0.052702	a
Gluconolactonase	EZ	0.482746	0.219056	a
Glucosamine-6-phosphate deaminase	AZ	0.783763	0.072476	a
Glucosamine-6-phosphate deaminase	BZ	0.802653	0.036455	a
Glucosamine-6-phosphate deaminase	CZ	0.041779	0.155641	ab
Glucosamine-6-phosphate deaminase	DZ	-0.63434	0.125425	bc
Glucosamine-6-phosphate deaminase	EZ	-0.99386	0.246864	c
Glucosamine-phosphate N-acetyltransferase	AZ	0.878719	0.088422	a
Glucosamine-phosphate N-acetyltransferase	BZ	0.973118	0.025689	a
Glucosamine-phosphate N-acetyltransferase	CZ	-0.33655	0.156037	b
Glucosamine-phosphate N-acetyltransferase	DZ	-0.84935	0.128979	b
Glucosamine-phosphate N-acetyltransferase	EZ	-0.66593	0.201684	b
Glucose-1-phosphate adenylyltransferase	AZ	-1.13404	0.171314	c
Glucose-1-phosphate adenylyltransferase	BZ	-0.52784	0.098721	bc
Glucose-1-phosphate adenylyltransferase	CZ	0.225731	0.096971	ab
Glucose-1-phosphate adenylyltransferase	DZ	1.029677	0.084282	a
Glucose-1-phosphate adenylyltransferase	EZ	0.406464	0.205782	ab
Glucose-6-phosphate 1-epimerase	AZ	-0.90841	0.123829	b
Glucose-6-phosphate 1-epimerase	BZ	-0.94353	0.150456	b
Glucose-6-phosphate 1-epimerase	CZ	0.638615	0.12396	a
Glucose-6-phosphate 1-epimerase	DZ	0.709639	0.07942	a
Glucose-6-phosphate 1-epimerase	EZ	0.50368	0.190599	a
Glucose-6-phosphate dehydrogenase (NADP(+))	AZ	0.863637	0.102324	a
Glucose-6-phosphate dehydrogenase (NADP(+))	BZ	0.958034	0.033545	a
Glucose-6-phosphate dehydrogenase (NADP(+))	CZ	-0.38893	0.173283	b
Glucose-6-phosphate dehydrogenase (NADP(+))	DZ	-0.74616	0.11751	b
Glucose-6-phosphate dehydrogenase (NADP(+))	EZ	-0.68658	0.205945	b
Glucose-6-phosphate isomerase	AZ	0.866802	0.103111	a
Glucose-6-phosphate isomerase	BZ	0.962972	0.033353	a
Glucose-6-phosphate isomerase	CZ	-0.38555	0.173323	b
Glucose-6-phosphate isomerase	DZ	-0.76463	0.109365	b
Glucose-6-phosphate isomerase	EZ	-0.67959	0.206309	b
Glucose-fructose oxidoreductase	AZ	-0.63625	0.057188	b
Glucose-fructose oxidoreductase	BZ	-1.36217	0.135688	c
Glucose-fructose oxidoreductase	CZ	0.731602	0.094291	a
Glucose-fructose oxidoreductase	DZ	0.615538	0.093711	a
Glucose-fructose oxidoreductase	EZ	0.651273	0.14393	a
Glucosylceramidase	AZ	-0.97169	0.162065	b
Glucosylceramidase	BZ	-0.7997	0.100039	b
Glucosylceramidase	CZ	0.427171	0.100955	a
Glucosylceramidase	DZ	0.870011	0.043178	a
Glucosylceramidase	EZ	0.47421	0.232965	a
Glucuronosyltransferase	AZ	-1.09081	0.145887	b

Glucuronosyltransferase	BZ	-0.72525	0.103126	b
Glucuronosyltransferase	CZ	0.615164	0.114142	a
Glucuronosyltransferase	DZ	0.74122	0.041721	a
Glucuronosyltransferase	EZ	0.459681	0.224862	a
Glutamate 5-kinase	AZ	0.866802	0.103111	a
Glutamate 5-kinase	BZ	0.962972	0.033353	a
Glutamate 5-kinase	CZ	-0.38555	0.173323	b
Glutamate 5-kinase	DZ	-0.76463	0.109365	b
Glutamate 5-kinase	EZ	-0.67959	0.206309	b
Glutamate carboxypeptidase II	AZ	-0.94952	0.157366	b
Glutamate carboxypeptidase II	BZ	-0.89225	0.099568	b
Glutamate carboxypeptidase II	CZ	0.569074	0.105251	a
Glutamate carboxypeptidase II	DZ	0.805314	0.050005	a
Glutamate carboxypeptidase II	EZ	0.467387	0.217525	a
Glutamate decarboxylase	AZ	0.660097	0.168691	a
Glutamate decarboxylase	BZ	0.736256	0.064393	a
Glutamate decarboxylase	CZ	-0.24374	0.1177	ab
Glutamate decarboxylase	DZ	-1.09078	0.240718	b
Glutamate decarboxylase	EZ	-0.06183	0.155102	ab
Glutamate dehydrogenase	AZ	0.867003	0.102901	a
Glutamate dehydrogenase	BZ	0.962252	0.033146	a
Glutamate dehydrogenase	CZ	-0.38285	0.172995	b
Glutamate dehydrogenase	DZ	-0.76985	0.110658	b
Glutamate dehydrogenase	EZ	-0.67656	0.205938	b
Glutamate dehydrogenase (NADP(+))	AZ	0.886955	0.097269	a
Glutamate dehydrogenase (NADP(+))	BZ	0.98034	0.028829	a
Glutamate dehydrogenase (NADP(+))	CZ	-0.3227	0.174557	b
Glutamate dehydrogenase (NADP(+))	DZ	-0.87218	0.061465	b
Glutamate dehydrogenase (NADP(+))	EZ	-0.67241	0.205482	b
Glutamate synthase (NADPH)	AZ	0.890243	0.078287	a
Glutamate synthase (NADPH)	BZ	0.954318	0.026527	a
Glutamate synthase (NADPH)	CZ	-0.50955	0.190823	b
Glutamate synthase (NADPH)	DZ	-0.74683	0.12109	b
Glutamate synthase (NADPH)	EZ	-0.58817	0.196878	b
Glutamate--ammonia ligase	AZ	0.554805	0.106874	ab
Glutamate--ammonia ligase	BZ	0.653671	0.045707	a
Glutamate--ammonia ligase	CZ	-0.78602	0.236465	c
Glutamate--ammonia ligase	DZ	0.214148	0.142552	abc
Glutamate--ammonia ligase	EZ	-0.6366	0.245051	bc
Glutamate--cysteine ligase	AZ	0.887212	0.089179	a
Glutamate--cysteine ligase	BZ	0.960341	0.027275	a
Glutamate--cysteine ligase	CZ	-0.59135	0.200393	b
Glutamate--cysteine ligase	DZ	-0.67322	0.109674	b
Glutamate--cysteine ligase	EZ	-0.58298	0.19107	b

Glutamate--tRNA ligase	AZ	0.848744	0.106068	a
Glutamate--tRNA ligase	BZ	0.954321	0.031294	a
Glutamate--tRNA ligase	CZ	-0.51568	0.203355	b
Glutamate--tRNA ligase	DZ	-0.66662	0.101607	b
Glutamate--tRNA ligase	EZ	-0.62077	0.199387	b
Glutamate-1-semialdehyde 2,1-aminomutase	AZ	-0.82627	0.149378	b
Glutamate-1-semialdehyde 2,1-aminomutase	BZ	-1.00032	0.099386	b
Glutamate-1-semialdehyde 2,1-aminomutase	CZ	0.592646	0.090828	a
Glutamate-1-semialdehyde 2,1-aminomutase	DZ	0.722774	0.0989	a
Glutamate-1-semialdehyde 2,1-aminomutase	EZ	0.511172	0.221049	a
Glutamate-5-semialdehyde dehydrogenase	AZ	0.867146	0.103132	a
Glutamate-5-semialdehyde dehydrogenase	BZ	0.96246	0.033215	a
Glutamate-5-semialdehyde dehydrogenase	CZ	-0.38545	0.173357	b
Glutamate-5-semialdehyde dehydrogenase	DZ	-0.7646	0.109387	b
Glutamate-5-semialdehyde dehydrogenase	EZ	-0.67955	0.206349	b
Glutamine--fructose-6-phosphate transaminase (isomerizing)	AZ	0.882772	0.101468	a
Glutamine--fructose-6-phosphate transaminase (isomerizing)	BZ	0.954976	0.0333	a
Glutamine--fructose-6-phosphate transaminase (isomerizing)	CZ	-0.38945	0.172247	b
Glutamine--fructose-6-phosphate transaminase (isomerizing)	DZ	-0.76095	0.107357	b
Glutamine--fructose-6-phosphate transaminase (isomerizing)	EZ	-0.68735	0.20659	b
Glutamine--tRNA ligase	AZ	0.866802	0.103111	a
Glutamine--tRNA ligase	BZ	0.962972	0.033353	a
Glutamine--tRNA ligase	CZ	-0.38555	0.173323	b
Glutamine--tRNA ligase	DZ	-0.76463	0.109365	b
Glutamine--tRNA ligase	EZ	-0.67959	0.206309	b
Glutathione peroxidase	AZ	0.837249	0.101346	a
Glutathione peroxidase	BZ	0.946523	0.03192	a
Glutathione peroxidase	CZ	-0.40272	0.196493	b
Glutathione peroxidase	DZ	-0.77642	0.106778	b
Glutathione peroxidase	EZ	-0.60463	0.20573	b
Glutathione synthase	AZ	0.818437	0.121571	a
Glutathione synthase	BZ	0.927729	0.042981	a
Glutathione synthase	CZ	-0.36716	0.160245	b
Glutathione synthase	DZ	-0.90335	0.110629	b
Glutathione synthase	EZ	-0.47566	0.223775	b
Glutathione transferase	AZ	0.753181	0.066262	a
Glutathione transferase	BZ	0.824104	0.036157	a
Glutathione transferase	CZ	-0.55059	0.263303	b
Glutathione transferase	DZ	-0.43061	0.161623	b
Glutathione transferase	EZ	-0.59609	0.185297	b
Gly-Xaa carboxypeptidase	AZ	-0.97161	0.162281	b
Gly-Xaa carboxypeptidase	BZ	-0.85201	0.092438	b
Gly-Xaa carboxypeptidase	CZ	0.60292	0.130163	a
Gly-Xaa carboxypeptidase	DZ	0.79761	0.040028	a

Gly-Xaa carboxypeptidase	EZ	0.423092	0.210102	a
Glycerate 3-kinase	AZ	-0.94296	0.156996	b
Glycerate 3-kinase	BZ	-0.97047	0.105375	b
Glycerate 3-kinase	CZ	0.814873	0.091633	a
Glycerate 3-kinase	DZ	0.646209	0.052277	a
Glycerate 3-kinase	EZ	0.45235	0.193024	a
Glycerate dehydrogenase	AZ	-0.92315	0.105171	b
Glycerate dehydrogenase	BZ	-0.99899	0.135507	b
Glycerate dehydrogenase	CZ	0.689992	0.105193	a
Glycerate dehydrogenase	DZ	0.59664	0.088575	a
Glycerate dehydrogenase	EZ	0.635508	0.193286	a
Glycerol 2-dehydrogenase (NADP(+))	AZ	-0.8922	0.087089	b
Glycerol 2-dehydrogenase (NADP(+))	BZ	-1.11274	0.077012	b
Glycerol 2-dehydrogenase (NADP(+))	CZ	0.699928	0.11094	a
Glycerol 2-dehydrogenase (NADP(+))	DZ	0.734206	0.087046	a
Glycerol 2-dehydrogenase (NADP(+))	EZ	0.570814	0.192742	a
Glycerol-1-phosphatase	AZ	-0.90508	0.156097	b
Glycerol-1-phosphatase	BZ	-0.88246	0.112363	b
Glycerol-1-phosphatase	CZ	0.517105	0.093165	a
Glycerol-1-phosphatase	DZ	0.79341	0.07873	a
Glycerol-1-phosphatase	EZ	0.47702	0.228064	a
Glycerol-3-phosphate 1-O-acyltransferase	AZ	0.852449	0.080906	a
Glycerol-3-phosphate 1-O-acyltransferase	BZ	0.914041	0.029837	a
Glycerol-3-phosphate 1-O-acyltransferase	CZ	-0.4674	0.207567	b
Glycerol-3-phosphate 1-O-acyltransferase	DZ	-0.71518	0.14645	b
Glycerol-3-phosphate 1-O-acyltransferase	EZ	-0.58391	0.190737	b
Glycerol-3-phosphate dehydrogenase (NAD(+))	AZ	0.743484	0.100913	a
Glycerol-3-phosphate dehydrogenase (NAD(+))	BZ	0.980619	0.030288	a
Glycerol-3-phosphate dehydrogenase (NAD(+))	CZ	-0.2905	0.160295	b
Glycerol-3-phosphate dehydrogenase (NAD(+))	DZ	-0.65612	0.175523	b
Glycerol-3-phosphate dehydrogenase (NAD(+))	EZ	-0.77748	0.201765	b
Glycerone kinase	AZ	-0.65592	0.183793	bc
Glycerone kinase	BZ	-0.90112	0.060421	c
Glycerone kinase	CZ	0.424093	0.180571	ab
Glycerone kinase	DZ	0.600098	0.137721	a
Glycerone kinase	EZ	0.532851	0.218377	a
Glycerone-phosphate O-acyltransferase	AZ	-1.10568	0.150598	b
Glycerone-phosphate O-acyltransferase	BZ	-0.68077	0.098675	b
Glycerone-phosphate O-acyltransferase	CZ	0.520744	0.091504	a
Glycerone-phosphate O-acyltransferase	DZ	0.936611	0.062127	a
Glycerone-phosphate O-acyltransferase	EZ	0.329091	0.219583	a
Glycerophosphodiester phosphodiesterase	AZ	0.740755	0.079716	a
Glycerophosphodiester phosphodiesterase	BZ	0.687938	0.139715	a
Glycerophosphodiester phosphodiesterase	CZ	0.096993	0.183431	ab

Glycerophosphodiester phosphodiesterase	DZ	-1.0501	0.145906	c
Glycerophosphodiester phosphodiesterase	EZ	-0.47559	0.202917	bc
Glycine amidinotransferase	AZ	-1.02877	0.15774	b
Glycine amidinotransferase	BZ	-0.22402	0.205929	ab
Glycine amidinotransferase	CZ	0.315487	0.184408	a
Glycine amidinotransferase	DZ	0.814878	0.11712	a
Glycine amidinotransferase	EZ	0.12243	0.175021	ab
Glycine C-acetyltransferase	AZ	-1.02877	0.15774	b
Glycine C-acetyltransferase	BZ	-0.22402	0.205929	ab
Glycine C-acetyltransferase	CZ	0.315487	0.184408	a
Glycine C-acetyltransferase	DZ	0.814878	0.11712	a
Glycine C-acetyltransferase	EZ	0.12243	0.175021	ab
Glycine dehydrogenase (aminomethyl-transferring)	AZ	0.869089	0.103772	a
Glycine dehydrogenase (aminomethyl-transferring)	BZ	0.966895	0.033148	a
Glycine dehydrogenase (aminomethyl-transferring)	CZ	-0.38175	0.173273	b
Glycine dehydrogenase (aminomethyl-transferring)	DZ	-0.7822	0.102128	b
Glycine dehydrogenase (aminomethyl-transferring)	EZ	-0.67203	0.206661	b
Glycine hydroxymethyltransferase	AZ	0.879192	0.094096	a
Glycine hydroxymethyltransferase	BZ	0.968567	0.027837	a
Glycine hydroxymethyltransferase	CZ	-0.4402	0.195687	b
Glycine hydroxymethyltransferase	DZ	-0.80937	0.094359	b
Glycine hydroxymethyltransferase	EZ	-0.59819	0.193092	b
Glycine--tRNA ligase	AZ	0.879942	0.103234	a
Glycine--tRNA ligase	BZ	0.954686	0.033673	a
Glycine--tRNA ligase	CZ	-0.40492	0.177225	b
Glycine--tRNA ligase	DZ	-0.78354	0.110004	b
Glycine--tRNA ligase	EZ	-0.64617	0.201597	b
Glycogen phosphorylase	AZ	0.867368	0.100021	a
Glycogen phosphorylase	BZ	0.958979	0.032277	a
Glycogen phosphorylase	CZ	-0.35531	0.16965	b
Glycogen phosphorylase	DZ	-0.76218	0.111714	b
Glycogen phosphorylase	EZ	-0.70886	0.208836	b
Glycogen(starch) synthase	AZ	0.866802	0.103111	a
Glycogen(starch) synthase	BZ	0.962972	0.033353	a
Glycogen(starch) synthase	CZ	-0.38555	0.173323	b
Glycogen(starch) synthase	DZ	-0.76463	0.109365	b
Glycogen(starch) synthase	EZ	-0.67959	0.206309	b
Glycogenin glucosyltransferase	AZ	-0.71781	0.171038	bc
Glycogenin glucosyltransferase	BZ	-0.97794	0.045777	c
Glycogenin glucosyltransferase	CZ	0.217543	0.12639	ab
Glycogenin glucosyltransferase	DZ	0.877773	0.127132	a
Glycogenin glucosyltransferase	EZ	0.60043	0.209801	a
Glycosylphosphatidylinositol phospholipase D	AZ	-0.83796	0.044477	b
Glycosylphosphatidylinositol phospholipase D	BZ	-0.73318	0.074604	b

Glycosylphosphatidylinositol phospholipase D	CZ	0.167932	0.216462	ab
Glycosylphosphatidylinositol phospholipase D	DZ	0.408171	0.198568	a
Glycosylphosphatidylinositol phospholipase D	EZ	0.995041	0.15792	a
Glycylpeptide N-tetradecanoyltransferase	AZ	0.860411	0.108978	a
Glycylpeptide N-tetradecanoyltransferase	BZ	0.960824	0.035645	a
Glycylpeptide N-tetradecanoyltransferase	CZ	-0.42267	0.181424	b
Glycylpeptide N-tetradecanoyltransferase	DZ	-0.82102	0.111135	b
Glycylpeptide N-tetradecanoyltransferase	EZ	-0.57755	0.197735	b
Glyoxylate reductase (NADP(+))	AZ	0.832569	0.05711	a
Glyoxylate reductase (NADP(+))	BZ	0.911065	0.015591	a
Glyoxylate reductase (NADP(+))	CZ	-0.38806	0.205374	b
Glyoxylate reductase (NADP(+))	DZ	-0.55479	0.138285	b
Glyoxylate reductase (NADP(+))	EZ	-0.80079	0.209884	b
GTP cyclohydrolase I	AZ	0.502608	0.129286	a
GTP cyclohydrolase I	BZ	0.840185	0.056291	a
GTP cyclohydrolase I	CZ	0.264424	0.122944	ab
GTP cyclohydrolase I	DZ	-0.88463	0.212123	c
GTP cyclohydrolase I	EZ	-0.72258	0.208201	bc
GTP cyclohydrolase II	AZ	0.866802	0.103111	a
GTP cyclohydrolase II	BZ	0.962972	0.033353	a
GTP cyclohydrolase II	CZ	-0.38555	0.173323	b
GTP cyclohydrolase II	DZ	-0.76463	0.109365	b
GTP cyclohydrolase II	EZ	-0.67959	0.206309	b
Guanidinoacetate N-methyltransferase	AZ	0.864899	0.080376	a
Guanidinoacetate N-methyltransferase	BZ	0.942408	0.024594	a
Guanidinoacetate N-methyltransferase	CZ	-0.56896	0.173898	b
Guanidinoacetate N-methyltransferase	DZ	-0.71101	0.130276	b
Guanidinoacetate N-methyltransferase	EZ	-0.52733	0.220333	b
Guanine deaminase	AZ	-0.90045	0.103174	b
Guanine deaminase	BZ	-0.93103	0.047377	b
Guanine deaminase	CZ	0.384686	0.178379	a
Guanine deaminase	DZ	0.867458	0.094841	a
Guanine deaminase	EZ	0.579337	0.20152	a
Guanylate cyclase	AZ	0.838082	0.03536	a
Guanylate cyclase	BZ	0.880644	0.009228	a
Guanylate cyclase	CZ	-0.10081	0.163378	b
Guanylate cyclase	DZ	-0.69486	0.16228	b
Guanylate cyclase	EZ	-0.92306	0.210927	b
Guanylate kinase	AZ	0.847386	0.094124	a
Guanylate kinase	BZ	0.913804	0.036424	a
Guanylate kinase	CZ	-0.10731	0.163427	b
Guanylate kinase	DZ	-0.93465	0.10847	b
Guanylate kinase	EZ	-0.71923	0.208724	b
H(+)-transporting two-sector ATPase	AZ	0.871739	0.085301	a

H(+)-transporting two-sector ATPase	BZ	0.95201	0.028266	a
H(+)-transporting two-sector ATPase	CZ	-0.44045	0.184047	b
H(+)-transporting two-sector ATPase	DZ	-0.70149	0.124387	b
H(+)-transporting two-sector ATPase	EZ	-0.68181	0.204254	b
Histidine--tRNA ligase	AZ	0.846183	0.095903	a
Histidine--tRNA ligase	BZ	0.918826	0.03666	a
Histidine--tRNA ligase	CZ	-0.10571	0.159574	b
Histidine--tRNA ligase	DZ	-0.93561	0.110181	b
Histidine--tRNA ligase	EZ	-0.72368	0.208155	b
Histidinol dehydrogenase	AZ	0.754485	0.063259	a
Histidinol dehydrogenase	BZ	0.864298	0.029804	a
Histidinol dehydrogenase	CZ	-0.65808	0.232453	b
Histidinol dehydrogenase	DZ	-0.66221	0.214736	b
Histidinol dehydrogenase	EZ	-0.2985	0.145317	b
Histidinol-phosphatase	AZ	0.86708	0.104723	a
Histidinol-phosphatase	BZ	0.96381	0.033726	a
Histidinol-phosphatase	CZ	-0.40334	0.175711	b
Histidinol-phosphatase	DZ	-0.77752	0.108906	b
Histidinol-phosphatase	EZ	-0.65003	0.204046	b
Histidinol-phosphate transaminase	AZ	0.866802	0.103111	a
Histidinol-phosphate transaminase	BZ	0.962972	0.033353	a
Histidinol-phosphate transaminase	CZ	-0.38555	0.173323	b
Histidinol-phosphate transaminase	DZ	-0.76463	0.109365	b
Histidinol-phosphate transaminase	EZ	-0.67959	0.206309	b
Histone-lysine N-methyltransferase	AZ	0.698396	0.139375	a
Histone-lysine N-methyltransferase	BZ	0.80603	0.08567	a
Histone-lysine N-methyltransferase	CZ	0.110542	0.152212	ab
Histone-lysine N-methyltransferase	DZ	-0.86805	0.114951	b
Histone-lysine N-methyltransferase	EZ	-0.74692	0.231949	b
Holo-[acyl-carrier-protein] synthase	AZ	-0.81688	0.122688	b
Holo-[acyl-carrier-protein] synthase	BZ	-1.04921	0.162693	b
Holo-[acyl-carrier-protein] synthase	CZ	0.720598	0.129473	a
Holo-[acyl-carrier-protein] synthase	DZ	0.670313	0.05779	a
Holo-[acyl-carrier-protein] synthase	EZ	0.475185	0.173924	a
Holocytochrome-c synthase	AZ	0.859762	0.101164	a
Holocytochrome-c synthase	BZ	0.932587	0.034701	a
Holocytochrome-c synthase	CZ	-0.37252	0.176517	b
Holocytochrome-c synthase	DZ	-0.75794	0.128018	b
Holocytochrome-c synthase	EZ	-0.66189	0.20737	b
Homoaconitate hydratase	AZ	0.70283	0.06329	a
Homoaconitate hydratase	BZ	0.798983	0.053649	a
Homoaconitate hydratase	CZ	0.154516	0.23536	ab
Homoaconitate hydratase	DZ	-1.10737	0.121496	c
Homoaconitate hydratase	EZ	-0.54896	0.169613	bc

Homocysteine S-methyltransferase	AZ	0.764268	0.075362	ab
Homocysteine S-methyltransferase	BZ	0.965279	0.012897	a
Homocysteine S-methyltransferase	CZ	-0.1681	0.148288	bc
Homocysteine S-methyltransferase	DZ	-0.75501	0.174565	c
Homocysteine S-methyltransferase	EZ	-0.80643	0.208486	c
Homogentisate 1,2-dioxygenase	AZ	-0.76467	0.04718	b
Homogentisate 1,2-dioxygenase	BZ	-0.7675	0.014576	b
Homogentisate 1,2-dioxygenase	CZ	0.328994	0.27445	ab
Homogentisate 1,2-dioxygenase	DZ	0.595655	0.139864	a
Homogentisate 1,2-dioxygenase	EZ	0.60752	0.205868	a
Homoisocitrate dehydrogenase	AZ	0.680118	0.124564	a
Homoisocitrate dehydrogenase	BZ	0.915172	0.042369	a
Homoisocitrate dehydrogenase	CZ	-0.46851	0.199321	b
Homoisocitrate dehydrogenase	DZ	-0.71011	0.183558	b
Homoisocitrate dehydrogenase	EZ	-0.41668	0.198476	b
Homoserine dehydrogenase	AZ	0.848623	0.10933	a
Homoserine dehydrogenase	BZ	0.947227	0.035437	a
Homoserine dehydrogenase	CZ	-0.40044	0.169118	b
Homoserine dehydrogenase	DZ	-0.72964	0.137083	b
Homoserine dehydrogenase	EZ	-0.66577	0.204278	b
Homoserine kinase	AZ	0.850086	0.086086	a
Homoserine kinase	BZ	0.904289	0.032431	a
Homoserine kinase	CZ	-0.30946	0.173493	b
Homoserine kinase	DZ	-0.62731	0.090667	b
Homoserine kinase	EZ	-0.81761	0.241474	b
Homoserine O-acetyltransferase	AZ	0.583458	0.080044	a
Homoserine O-acetyltransferase	BZ	0.575154	0.067483	a
Homoserine O-acetyltransferase	CZ	0.642803	0.164972	a
Homoserine O-acetyltransferase	DZ	-1.05372	0.120574	b
Homoserine O-acetyltransferase	EZ	-0.74769	0.2227	b
Hydroxyacid-oxoacid transhydrogenase	AZ	-0.90841	0.123826	b
Hydroxyacid-oxoacid transhydrogenase	BZ	-0.94353	0.150452	b
Hydroxyacid-oxoacid transhydrogenase	CZ	0.638575	0.123957	a
Hydroxyacid-oxoacid transhydrogenase	DZ	0.709598	0.079418	a
Hydroxyacid-oxoacid transhydrogenase	EZ	0.503772	0.190606	a
Hydroxyacylglutathione hydrolase	AZ	0.848957	0.094514	a
Hydroxyacylglutathione hydrolase	BZ	0.961106	0.026955	a
Hydroxyacylglutathione hydrolase	CZ	-0.37061	0.167907	b
Hydroxyacylglutathione hydrolase	DZ	-0.58017	0.095068	b
Hydroxyacylglutathione hydrolase	EZ	-0.85929	0.224212	b
Hydroxymethylglutaryl-CoA lyase	AZ	0.766342	0.142327	ab
Hydroxymethylglutaryl-CoA lyase	BZ	0.840507	0.057434	a
Hydroxymethylglutaryl-CoA lyase	CZ	-0.22289	0.201637	bc
Hydroxymethylglutaryl-CoA lyase	DZ	-0.97595	0.125913	c

Hydroxymethylglutaryl-CoA lyase	EZ	-0.40801	0.191871	c
Hydroxymethylglutaryl-CoA reductase (NADPH)	AZ	0.713162	0.124354	a
Hydroxymethylglutaryl-CoA reductase (NADPH)	BZ	0.919731	0.040759	a
Hydroxymethylglutaryl-CoA reductase (NADPH)	CZ	-0.58558	0.21331	b
Hydroxymethylglutaryl-CoA reductase (NADPH)	DZ	-0.5458	0.143556	b
Hydroxymethylglutaryl-CoA reductase (NADPH)	EZ	-0.50151	0.21077	b
Hydroxyquinol 1,2-dioxygenase	AZ	-0.90489	0.158492	b
Hydroxyquinol 1,2-dioxygenase	BZ	-0.92398	0.085344	b
Hydroxyquinol 1,2-dioxygenase	CZ	0.505369	0.112323	a
Hydroxyquinol 1,2-dioxygenase	DZ	0.872448	0.032484	a
Hydroxyquinol 1,2-dioxygenase	EZ	0.451061	0.222025	a
Hypoxanthine phosphoribosyltransferase	AZ	0.835083	0.040204	a
Hypoxanthine phosphoribosyltransferase	BZ	0.883621	0.010458	a
Hypoxanthine phosphoribosyltransferase	CZ	-0.14654	0.161629	b
Hypoxanthine phosphoribosyltransferase	DZ	-0.73186	0.183458	b
Hypoxanthine phosphoribosyltransferase	EZ	-0.8403	0.203534	b
IgA-specific metalloendopeptidase	AZ	-0.87422	0.120872	b
IgA-specific metalloendopeptidase	BZ	-0.93975	0.172442	b
IgA-specific metalloendopeptidase	CZ	0.653094	0.124925	a
IgA-specific metalloendopeptidase	DZ	0.610103	0.097405	a
IgA-specific metalloendopeptidase	EZ	0.550776	0.181891	a
Imidazoleglycerol-phosphate dehydratase	AZ	0.762753	0.082782	a
Imidazoleglycerol-phosphate dehydratase	BZ	0.870541	0.02466	a
Imidazoleglycerol-phosphate dehydratase	CZ	-0.12155	0.13245	ab
Imidazoleglycerol-phosphate dehydratase	DZ	-0.68041	0.178758	b
Imidazoleglycerol-phosphate dehydratase	EZ	-0.83133	0.240037	b
Inorganic diphosphatase	AZ	-0.77736	0.026757	c
Inorganic diphosphatase	BZ	-0.4537	0.118564	bc
Inorganic diphosphatase	CZ	0.556349	0.199015	ab
Inorganic diphosphatase	DZ	0.853477	0.209573	a
Inorganic diphosphatase	EZ	-0.17876	0.218396	abc
Inositol 2-dehydrogenase	AZ	-0.96295	0.171554	c
Inositol 2-dehydrogenase	BZ	-0.65356	0.121085	bc
Inositol 2-dehydrogenase	CZ	0.592974	0.173922	a
Inositol 2-dehydrogenase	DZ	0.665423	0.031416	a
Inositol 2-dehydrogenase	EZ	0.358119	0.225428	ab
Inositol 3-alpha-galactosyltransferase	AZ	-0.92351	0.121566	b
Inositol 3-alpha-galactosyltransferase	BZ	-0.94531	0.145981	b
Inositol 3-alpha-galactosyltransferase	CZ	0.630319	0.118958	a
Inositol 3-alpha-galactosyltransferase	DZ	0.757608	0.08183	a
Inositol 3-alpha-galactosyltransferase	EZ	0.480892	0.188716	a
Inositol oxygenase	AZ	-0.97914	0.158775	b
Inositol oxygenase	BZ	-0.83835	0.093389	b
Inositol oxygenase	CZ	0.561252	0.110448	a

Inositol oxygenase	DZ	0.833681	0.04264	a
Inositol oxygenase	EZ	0.422555	0.222589	a
Inositol-3-phosphate synthase	AZ	0.866506	0.102672	a
Inositol-3-phosphate synthase	BZ	0.962563	0.033222	a
Inositol-3-phosphate synthase	CZ	-0.38382	0.173331	b
Inositol-3-phosphate synthase	DZ	-0.76722	0.110277	b
Inositol-3-phosphate synthase	EZ	-0.67802	0.206152	b
Inositol-phosphate phosphatase	AZ	-0.81442	0.13647	b
Inositol-phosphate phosphatase	BZ	-0.94213	0.052202	b
Inositol-phosphate phosphatase	CZ	0.388457	0.120552	a
Inositol-phosphate phosphatase	DZ	0.809627	0.173649	a
Inositol-phosphate phosphatase	EZ	0.558472	0.201258	a
Inositol-polyphosphate 5-phosphatase	AZ	-0.78371	0.069997	b
Inositol-polyphosphate 5-phosphatase	BZ	-0.73838	0.043541	b
Inositol-polyphosphate 5-phosphatase	CZ	0.210094	0.184282	ab
Inositol-polyphosphate 5-phosphatase	DZ	0.870315	0.236884	a
Inositol-polyphosphate 5-phosphatase	EZ	0.441681	0.187955	a
Insulysin	AZ	0.863141	0.082482	a
Insulysin	BZ	0.973343	0.023398	a
Insulysin	CZ	-0.39336	0.172294	b
Insulysin	DZ	-0.66787	0.121765	b
Insulysin	EZ	-0.77526	0.208391	b
Isocitrate dehydrogenase (NAD(+))	AZ	0.814465	0.10194	a
Isocitrate dehydrogenase (NAD(+))	BZ	0.980395	0.023461	a
Isocitrate dehydrogenase (NAD(+))	CZ	-0.35429	0.165675	b
Isocitrate dehydrogenase (NAD(+))	DZ	-0.70955	0.139457	b
Isocitrate dehydrogenase (NAD(+))	EZ	-0.73102	0.2068	b
Isocitrate dehydrogenase (NADP(+))	AZ	0.863637	0.102324	a
Isocitrate dehydrogenase (NADP(+))	BZ	0.958034	0.033545	a
Isocitrate dehydrogenase (NADP(+))	CZ	-0.38893	0.173283	b
Isocitrate dehydrogenase (NADP(+))	DZ	-0.74616	0.11751	b
Isocitrate dehydrogenase (NADP(+))	EZ	-0.68658	0.205945	b
Isocitrate lyase	AZ	-0.88237	0.104643	b
Isocitrate lyase	BZ	-0.96915	0.045289	b
Isocitrate lyase	CZ	0.40893	0.168197	a
Isocitrate lyase	DZ	0.765374	0.099571	a
Isocitrate lyase	EZ	0.67722	0.205718	a
Isoleucine--tRNA ligase	AZ	-0.88711	0.102329	b
Isoleucine--tRNA ligase	BZ	-0.96993	0.044881	b
Isoleucine--tRNA ligase	CZ	0.398805	0.171346	a
Isoleucine--tRNA ligase	DZ	0.872193	0.101672	a
Isoleucine--tRNA ligase	EZ	0.586039	0.195631	a
isomerase	AZ	0.866802	0.103111	a
isomerase	BZ	0.962972	0.033353	a

isomerase	CZ	-0.38555	0.173323	b
isomerase	DZ	-0.76463	0.109365	b
isomerase	EZ	-0.67959	0.206309	b
Isopentenyl-diphosphate Delta-isomerase	AZ	0.866802	0.103111	a
Isopentenyl-diphosphate Delta-isomerase	BZ	0.962972	0.033353	a
Isopentenyl-diphosphate Delta-isomerase	CZ	-0.38555	0.173323	b
Isopentenyl-diphosphate Delta-isomerase	DZ	-0.76463	0.109365	b
Isopentenyl-diphosphate Delta-isomerase	EZ	-0.67959	0.206309	b
Kanamycin kinase	AZ	-0.56855	0.054568	bc
Kanamycin kinase	BZ	-0.98743	0.147652	c
Kanamycin kinase	CZ	0.558435	0.029317	a
Kanamycin kinase	DZ	0.079358	0.257277	ab
Kanamycin kinase	EZ	0.918183	0.158736	a
Ketohexokinase	AZ	-1.07283	0.153018	b
Ketohexokinase	BZ	-0.81885	0.072479	b
Ketohexokinase	CZ	0.582827	0.083985	a
Ketohexokinase	DZ	0.893941	0.046879	a
Ketohexokinase	EZ	0.414906	0.213212	a
Ketol-acid reductoisomerase (NADP(+))	AZ	0.867261	0.104825	a
Ketol-acid reductoisomerase (NADP(+))	BZ	0.965196	0.034063	a
Ketol-acid reductoisomerase (NADP(+))	CZ	-0.38819	0.171743	b
Ketol-acid reductoisomerase (NADP(+))	DZ	-0.79403	0.111076	b
Ketol-acid reductoisomerase (NADP(+))	EZ	-0.65024	0.204038	b
Kexin	AZ	0.866305	0.103175	a
Kexin	BZ	0.962465	0.03336	a
Kexin	CZ	-0.38194	0.173266	b
Kexin	DZ	-0.76601	0.109416	b
Kexin	EZ	-0.68082	0.206397	b
Kynurenine 3-monooxygenase	AZ	-0.82065	0.138128	b
Kynurenine 3-monooxygenase	BZ	-0.92276	0.056226	b
Kynurenine 3-monooxygenase	CZ	0.277741	0.136488	a
Kynurenine 3-monooxygenase	DZ	0.92687	0.14067	a
Kynurenine 3-monooxygenase	EZ	0.538803	0.20437	a
Kynurenine--oxoglutarate transaminase	AZ	-0.93179	0.116355	b
Kynurenine--oxoglutarate transaminase	BZ	-0.97333	0.141017	b
Kynurenine--oxoglutarate transaminase	CZ	0.620966	0.106981	a
Kynurenine--oxoglutarate transaminase	DZ	0.723584	0.077079	a
Kynurenine--oxoglutarate transaminase	EZ	0.560573	0.192973	a
L-2-hydroxyglutarate dehydrogenase	AZ	-0.89841	0.105653	b
L-2-hydroxyglutarate dehydrogenase	BZ	-1.07924	0.085318	b
L-2-hydroxyglutarate dehydrogenase	CZ	0.691622	0.107711	a
L-2-hydroxyglutarate dehydrogenase	DZ	0.656412	0.098499	a
L-2-hydroxyglutarate dehydrogenase	EZ	0.629612	0.190731	a
L-amino-acid oxidase	AZ	-0.96289	0.116594	b

L-amino-acid oxidase	BZ	-0.91295	0.152111	b
L-amino-acid oxidase	CZ	0.67106	0.090439	a
L-amino-acid oxidase	DZ	0.526675	0.072801	a
L-amino-acid oxidase	EZ	0.678098	0.205868	a
L-aminoadipate-semialdehyde dehydrogenase	AZ	-0.92299	0.122024	b
L-aminoadipate-semialdehyde dehydrogenase	BZ	-0.908	0.075158	b
L-aminoadipate-semialdehyde dehydrogenase	CZ	0.379929	0.159509	a
L-aminoadipate-semialdehyde dehydrogenase	DZ	0.957793	0.076376	a
L-aminoadipate-semialdehyde dehydrogenase	EZ	0.49327	0.197774	a
L-arabinose isomerase	AZ	-0.88564	0.147881	b
L-arabinose isomerase	BZ	-0.92246	0.071505	b
L-arabinose isomerase	CZ	0.465933	0.15234	a
L-arabinose isomerase	DZ	0.778966	0.075426	a
L-arabinose isomerase	EZ	0.563202	0.212846	a
L-ascorbate oxidase	AZ	-0.91297	0.146497	b
L-ascorbate oxidase	BZ	-0.94114	0.096227	b
L-ascorbate oxidase	CZ	0.621916	0.095824	a
L-ascorbate oxidase	DZ	0.7878	0.068405	a
L-ascorbate oxidase	EZ	0.444401	0.221831	a
L-ascorbate peroxidase	AZ	-0.8802	0.18055	b
L-ascorbate peroxidase	BZ	-0.90346	0.099711	b
L-ascorbate peroxidase	CZ	0.526333	0.095779	a
L-ascorbate peroxidase	DZ	0.832041	0.054541	a
L-ascorbate peroxidase	EZ	0.425292	0.219662	a
L-fuculose-phosphate aldolase	AZ	-0.88483	0.169957	b
L-fuculose-phosphate aldolase	BZ	-0.81814	0.108604	b
L-fuculose-phosphate aldolase	CZ	0.478919	0.109697	a
L-fuculose-phosphate aldolase	DZ	0.695007	0.073641	a
L-fuculose-phosphate aldolase	EZ	0.529041	0.244204	a
L-gulonolactone oxidase	AZ	0.816816	0.073282	a
L-gulonolactone oxidase	BZ	0.87899	0.025557	a
L-gulonolactone oxidase	CZ	-0.11123	0.161028	ab
L-gulonolactone oxidase	DZ	-0.8206	0.164824	b
L-gulonolactone oxidase	EZ	-0.76398	0.213115	b
L-iditol 2-dehydrogenase	AZ	-0.89233	0.13822	b
L-iditol 2-dehydrogenase	BZ	-0.96155	0.063016	b
L-iditol 2-dehydrogenase	CZ	0.478347	0.140437	a
L-iditol 2-dehydrogenase	DZ	0.782741	0.074807	a
L-iditol 2-dehydrogenase	EZ	0.592788	0.214288	a
L-lactate dehydrogenase	AZ	0.835331	0.056618	a
L-lactate dehydrogenase	BZ	0.897736	0.015074	a
L-lactate dehydrogenase	CZ	-0.44808	0.205848	b
L-lactate dehydrogenase	DZ	-0.45139	0.137642	b
L-lactate dehydrogenase	EZ	-0.8336	0.212349	b

L-lactate dehydrogenase (cytochrome)	AZ	-0.86875	0.132781	b
L-lactate dehydrogenase (cytochrome)	BZ	-1.00209	0.050468	b
L-lactate dehydrogenase (cytochrome)	CZ	0.534386	0.150581	a
L-lactate dehydrogenase (cytochrome)	DZ	0.755237	0.085508	a
L-lactate dehydrogenase (cytochrome)	EZ	0.581222	0.20543	a
L-rhamnonate dehydratase	AZ	-0.90841	0.123829	b
L-rhamnonate dehydratase	BZ	-0.94353	0.150456	b
L-rhamnonate dehydratase	CZ	0.638615	0.12396	a
L-rhamnonate dehydratase	DZ	0.709639	0.07942	a
L-rhamnonate dehydratase	EZ	0.50368	0.190599	a
L-rhamnose 1-dehydrogenase	AZ	-0.91262	0.107057	b
L-rhamnose 1-dehydrogenase	BZ	-0.99384	0.155067	b
L-rhamnose 1-dehydrogenase	CZ	0.640599	0.093949	a
L-rhamnose 1-dehydrogenase	DZ	0.562243	0.077814	a
L-rhamnose 1-dehydrogenase	EZ	0.703613	0.193661	a
L-threonine 3-dehydrogenase	AZ	-0.90214	0.14682	b
L-threonine 3-dehydrogenase	BZ	-0.92798	0.094258	b
L-threonine 3-dehydrogenase	CZ	0.611383	0.104293	a
L-threonine 3-dehydrogenase	DZ	0.770964	0.050746	a
L-threonine 3-dehydrogenase	EZ	0.447769	0.232215	a
L-xylulose reductase	AZ	-0.58227	0.053901	bc
L-xylulose reductase	BZ	-0.99602	0.145848	c
L-xylulose reductase	CZ	0.626391	0.015944	a
L-xylulose reductase	DZ	0.062576	0.254921	ab
L-xylulose reductase	EZ	0.889319	0.157069	a
Laccase	AZ	-0.79613	0.166727	b
Laccase	BZ	-0.95413	0.118635	b
Laccase	CZ	0.51738	0.115006	a
Laccase	DZ	0.722328	0.052939	a
Laccase	EZ	0.51055	0.228877	a
Lactate 2-monooxygenase	AZ	-0.90574	0.108949	b
Lactate 2-monooxygenase	BZ	-0.9889	0.141027	b
Lactate 2-monooxygenase	CZ	0.698441	0.115014	a
Lactate 2-monooxygenase	DZ	0.614684	0.096876	a
Lactate 2-monooxygenase	EZ	0.581517	0.188475	a
Lactoylglutathione lyase	AZ	-0.82744	0.024913	c
Lactoylglutathione lyase	BZ	-0.57541	0.062442	bc
Lactoylglutathione lyase	CZ	0.84655	0.239566	a
Lactoylglutathione lyase	DZ	0.446056	0.153438	ab
Lactoylglutathione lyase	EZ	0.110241	0.239519	abc
Lanosterol synthase	AZ	0.816724	0.075452	a
Lanosterol synthase	BZ	0.9721	0.039344	a
Lanosterol synthase	CZ	-0.32409	0.165026	b
Lanosterol synthase	DZ	-0.67547	0.153211	b

Lanosterol synthase	EZ	-0.78926	0.205524	b
Leucine--tRNA ligase	AZ	0.787016	0.1364	a
Leucine--tRNA ligase	BZ	0.977418	0.034404	a
Leucine--tRNA ligase	CZ	-0.43569	0.193962	b
Leucine--tRNA ligase	DZ	-0.71738	0.100598	b
Leucine--tRNA ligase	EZ	-0.61136	0.199555	b
Leucyl aminopeptidase	AZ	0.829356	0.056454	a
Leucyl aminopeptidase	BZ	0.891443	0.016657	a
Leucyl aminopeptidase	CZ	-0.10452	0.146064	b
Leucyl aminopeptidase	DZ	-0.68794	0.163947	b
Leucyl aminopeptidase	EZ	-0.92834	0.216557	b
Leukotriene-A(4) hydrolase	AZ	0.8673	0.104805	a
Leukotriene-A(4) hydrolase	BZ	0.965083	0.034072	a
Leukotriene-A(4) hydrolase	CZ	-0.38881	0.171913	b
Leukotriene-A(4) hydrolase	DZ	-0.79366	0.111054	b
Leukotriene-A(4) hydrolase	EZ	-0.64991	0.203998	b
Levanase	AZ	-0.63547	0.05723	b
Levanase	BZ	-1.36193	0.135788	c
Levanase	CZ	0.733389	0.094361	a
Levanase	DZ	0.614078	0.094694	a
Levanase	EZ	0.649931	0.143575	a
Licheninase	AZ	-0.86112	0.127684	b
Licheninase	BZ	-0.93957	0.054852	b
Licheninase	CZ	0.33023	0.14056	a
Licheninase	DZ	0.854321	0.119021	a
Licheninase	EZ	0.616145	0.211236	a
Long-chain-alcohol oxidase	AZ	-0.90825	0.123915	b
Long-chain-alcohol oxidase	BZ	-0.9434	0.150561	b
Long-chain-alcohol oxidase	CZ	0.639849	0.124046	a
Long-chain-alcohol oxidase	DZ	0.709765	0.079869	a
Long-chain-alcohol oxidase	EZ	0.502036	0.190272	a
Long-chain-fatty-acid--CoA ligase	AZ	0.764235	0.104542	a
Long-chain-fatty-acid--CoA ligase	BZ	0.947359	0.046065	a
Long-chain-fatty-acid--CoA ligase	CZ	-0.46494	0.190881	b
Long-chain-fatty-acid--CoA ligase	DZ	-0.44072	0.099543	b
Long-chain-fatty-acid--CoA ligase	EZ	-0.80593	0.231367	b
Lysine 2,3-aminomutase	AZ	-0.87941	0.134089	b
Lysine 2,3-aminomutase	BZ	-0.38624	0.146626	b
Lysine 2,3-aminomutase	CZ	0.273466	0.184423	ab
Lysine 2,3-aminomutase	DZ	0.115406	0.155456	ab
Lysine 2,3-aminomutase	EZ	0.876778	0.231027	a
Lysine--tRNA ligase	AZ	0.872356	0.093393	a
Lysine--tRNA ligase	BZ	0.978133	0.024659	a
Lysine--tRNA ligase	CZ	-0.53911	0.204674	b

Lysine--tRNA ligase	DZ	-0.7395	0.0945	b
Lysine--tRNA ligase	EZ	-0.57187	0.189032	b
Lysophospholipase	AZ	-0.87467	0.125036	b
Lysophospholipase	BZ	-0.988	0.048741	b
Lysophospholipase	CZ	0.49879	0.158211	a
Lysophospholipase	DZ	0.802268	0.095393	a
Lysophospholipase	EZ	0.561608	0.20102	a
Lysosomal Pro-Xaa carboxypeptidase	AZ	-1.10011	0.155997	c
Lysosomal Pro-Xaa carboxypeptidase	BZ	-0.50578	0.114711	bc
Lysosomal Pro-Xaa carboxypeptidase	CZ	0.222494	0.12128	ab
Lysosomal Pro-Xaa carboxypeptidase	DZ	1.068699	0.0831	a
Lysosomal Pro-Xaa carboxypeptidase	EZ	0.314689	0.207503	ab
Lysozyme	AZ	-0.72824	0.069862	b
Lysozyme	BZ	-0.77056	0.071886	b
Lysozyme	CZ	0.61804	0.033576	a
Lysozyme	DZ	-0.03068	0.280935	ab
Lysozyme	EZ	0.911445	0.181693	a
Malate dehydrogenase (oxaloacetate-decarboxylating)	AZ	0.874139	0.07087	a
Malate dehydrogenase (oxaloacetate-decarboxylating)	BZ	0.951658	0.019632	a
Malate dehydrogenase (oxaloacetate-decarboxylating)	CZ	-0.4211	0.214259	b
Malate dehydrogenase (oxaloacetate-decarboxylating)	DZ	-0.79528	0.106409	b
Malate dehydrogenase (oxaloacetate-decarboxylating)	EZ	-0.60941	0.186586	b
Malate dehydrogenase (oxaloacetate-decarboxylating) (NADP(+))	AZ	-0.93309	0.153607	b
Malate dehydrogenase (oxaloacetate-decarboxylating) (NADP(+))	BZ	-0.91404	0.092675	b
Malate dehydrogenase (oxaloacetate-decarboxylating) (NADP(+))	CZ	0.544251	0.105521	a
Malate dehydrogenase (oxaloacetate-decarboxylating) (NADP(+))	DZ	0.833128	0.051043	a
Malate dehydrogenase (oxaloacetate-decarboxylating) (NADP(+))	EZ	0.469751	0.219593	a
Maleylacetoacetate isomerase	AZ	0.803006	0.075845	a
Maleylacetoacetate isomerase	BZ	0.889531	0.020849	a
Maleylacetoacetate isomerase	CZ	-0.30794	0.18419	b
Maleylacetoacetate isomerase	DZ	-0.86002	0.194105	b
Maleylacetoacetate isomerase	EZ	-0.52457	0.186879	b
Malonyl-CoA decarboxylase	AZ	0.835083	0.040204	a
Malonyl-CoA decarboxylase	BZ	0.883621	0.010458	a
Malonyl-CoA decarboxylase	CZ	-0.14654	0.161629	b
Malonyl-CoA decarboxylase	DZ	-0.73186	0.183458	b
Malonyl-CoA decarboxylase	EZ	-0.8403	0.203534	b
Maltose O-acetyltransferase	AZ	-0.40138	0.107781	ab
Maltose O-acetyltransferase	BZ	-0.99485	0.104069	b
Maltose O-acetyltransferase	CZ	0.275518	0.114317	a
Maltose O-acetyltransferase	DZ	0.542859	0.290228	a
Maltose O-acetyltransferase	EZ	0.577851	0.165784	a
Mannan endo-1,4-beta-mannosidase	AZ	-0.96894	0.083718	b
Mannan endo-1,4-beta-mannosidase	BZ	-1.08614	0.066436	b

Mannan endo-1,4-beta-mannosidase	CZ	0.696238	0.074219	a
Mannan endo-1,4-beta-mannosidase	DZ	0.655821	0.090385	a
Mannan endo-1,4-beta-mannosidase	EZ	0.703022	0.19626	a
Mannan endo-1,6-alpha-mannosidase	AZ	-0.90337	0.158498	b
Mannan endo-1,6-alpha-mannosidase	BZ	-0.88813	0.098328	b
Mannan endo-1,6-alpha-mannosidase	CZ	0.540941	0.095431	a
Mannan endo-1,6-alpha-mannosidase	DZ	0.80791	0.077104	a
Mannan endo-1,6-alpha-mannosidase	EZ	0.442645	0.229896	a
Mannitol 2-dehydrogenase	AZ	-0.85175	0.088809	b
Mannitol 2-dehydrogenase	BZ	-1.17153	0.052559	b
Mannitol 2-dehydrogenase	CZ	0.744356	0.11485	a
Mannitol 2-dehydrogenase	DZ	0.664187	0.100404	a
Mannitol 2-dehydrogenase	EZ	0.614744	0.180345	a
Mannitol-1-phosphate 5-dehydrogenase	AZ	-0.88994	0.116957	b
Mannitol-1-phosphate 5-dehydrogenase	BZ	-0.97131	0.155248	b
Mannitol-1-phosphate 5-dehydrogenase	CZ	0.642625	0.124684	a
Mannitol-1-phosphate 5-dehydrogenase	DZ	0.692707	0.083325	a
Mannitol-1-phosphate 5-dehydrogenase	EZ	0.525917	0.185711	a
Mannose-1-phosphate guanylyltransferase	AZ	0.892891	0.095873	a
Mannose-1-phosphate guanylyltransferase	BZ	0.984546	0.026433	a
Mannose-1-phosphate guanylyltransferase	CZ	-0.46055	0.181688	b
Mannose-1-phosphate guanylyltransferase	DZ	-0.82235	0.093869	b
Mannose-1-phosphate guanylyltransferase	EZ	-0.59453	0.194593	b
Mannose-6-phosphate isomerase	AZ	-0.78587	0.137864	b
Mannose-6-phosphate isomerase	BZ	-0.92432	0.047658	b
Mannose-6-phosphate isomerase	CZ	0.320073	0.117336	a
Mannose-6-phosphate isomerase	DZ	0.889204	0.195878	a
Mannose-6-phosphate isomerase	EZ	0.500905	0.189175	a
Mannosyl-oligosaccharide 1,2-alpha-mannosidase	AZ	-0.75331	0.140769	b
Mannosyl-oligosaccharide 1,2-alpha-mannosidase	BZ	-0.95852	0.049261	b
Mannosyl-oligosaccharide 1,2-alpha-mannosidase	CZ	0.362882	0.136389	a
Mannosyl-oligosaccharide 1,2-alpha-mannosidase	DZ	0.665272	0.160663	a
Mannosyl-oligosaccharide 1,2-alpha-mannosidase	EZ	0.683677	0.216672	a
Mannosyl-oligosaccharide glucosidase	AZ	-0.71379	0.145334	b
Mannosyl-oligosaccharide glucosidase	BZ	-0.81453	0.054175	b
Mannosyl-oligosaccharide glucosidase	CZ	0.245829	0.107172	ab
Mannosyl-oligosaccharide glucosidase	DZ	0.620812	0.184692	a
Mannosyl-oligosaccharide glucosidase	EZ	0.661677	0.259838	a
Membrane dipeptidase	AZ	-0.95878	0.165463	b
Membrane dipeptidase	BZ	-0.84528	0.096199	b
Membrane dipeptidase	CZ	0.574712	0.110587	a
Membrane dipeptidase	DZ	0.77046	0.050437	a
Membrane dipeptidase	EZ	0.458887	0.224015	a
Methionine adenosyltransferase	AZ	0.735541	0.133095	a

Methionine adenosyltransferase	BZ	0.949577	0.039107	a
Methionine adenosyltransferase	CZ	-0.28371	0.163739	b
Methionine adenosyltransferase	DZ	-0.65729	0.146881	b
Methionine adenosyltransferase	EZ	-0.74412	0.21662	b
Methionine synthase	AZ	0.835083	0.040204	a
Methionine synthase	BZ	0.883621	0.010458	a
Methionine synthase	CZ	-0.14654	0.161629	b
Methionine synthase	DZ	-0.73186	0.183458	b
Methionine synthase	EZ	-0.8403	0.203534	b
Methionine--tRNA ligase	AZ	0.86678	0.103121	a
Methionine--tRNA ligase	BZ	0.963025	0.033349	a
Methionine--tRNA ligase	CZ	-0.38524	0.173243	b
Methionine--tRNA ligase	DZ	-0.7648	0.109375	b
Methionine--tRNA ligase	EZ	-0.67976	0.206328	b
Methionyl aminopeptidase	AZ	0.774253	0.118975	a
Methionyl aminopeptidase	BZ	0.950757	0.031242	a
Methionyl aminopeptidase	CZ	-0.31149	0.15179	b
Methionyl aminopeptidase	DZ	-0.58439	0.132942	b
Methionyl aminopeptidase	EZ	-0.82913	0.229533	b
Methionyl-tRNA formyltransferase	AZ	0.70152	0.075257	ab
Methionyl-tRNA formyltransferase	BZ	0.787016	0.021065	a
Methionyl-tRNA formyltransferase	CZ	-0.11762	0.107142	abc
Methionyl-tRNA formyltransferase	DZ	-1.02506	0.295961	c
Methionyl-tRNA formyltransferase	EZ	-0.34586	0.141542	bc
Methylated-DNA--[protein]-cysteine S-methyltransferase	AZ	-0.61109	0.181739	bc
Methylated-DNA--[protein]-cysteine S-methyltransferase	BZ	-0.94274	0.17628	c
Methylated-DNA--[protein]-cysteine S-methyltransferase	CZ	0.283737	0.070214	ab
Methylated-DNA--[protein]-cysteine S-methyltransferase	DZ	0.871052	0.111061	a
Methylated-DNA--[protein]-cysteine S-methyltransferase	EZ	0.399035	0.21354	ab
Methylcrotonoyl-CoA carboxylase	AZ	0.881027	0.095647	a
Methylcrotonoyl-CoA carboxylase	BZ	0.972267	0.029421	a
Methylcrotonoyl-CoA carboxylase	CZ	-0.34119	0.163498	b
Methylcrotonoyl-CoA carboxylase	DZ	-0.87265	0.117712	b
Methylcrotonoyl-CoA carboxylase	EZ	-0.63946	0.197436	b
Methylene-fatty-acyl-phospholipid synthase	AZ	-0.89978	0.157162	b
Methylene-fatty-acyl-phospholipid synthase	BZ	-0.86893	0.111069	b
Methylene-fatty-acyl-phospholipid synthase	CZ	0.632661	0.11099	a
Methylene-fatty-acyl-phospholipid synthase	DZ	0.722048	0.074125	a
Methylene-fatty-acyl-phospholipid synthase	EZ	0.414005	0.228621	a
Methylenetetrahydrofolate dehydrogenase (NAD(+))	AZ	0.866777	0.104822	a
Methylenetetrahydrofolate dehydrogenase (NAD(+))	BZ	0.964489	0.033905	a
Methylenetetrahydrofolate dehydrogenase (NAD(+))	CZ	-0.40485	0.175879	b
Methylenetetrahydrofolate dehydrogenase (NAD(+))	DZ	-0.77899	0.109166	b
Methylenetetrahydrofolate dehydrogenase (NAD(+))	EZ	-0.64743	0.20357	b

Methylenetetrahydrofolate reductase (NAD(P)H)	AZ	0.866735	0.102996	a
Methylenetetrahydrofolate reductase (NAD(P)H)	BZ	0.962873	0.033319	a
Methylenetetrahydrofolate reductase (NAD(P)H)	CZ	-0.38425	0.173143	b
Methylenetetrahydrofolate reductase (NAD(P)H)	DZ	-0.76726	0.109994	b
Methylenetetrahydrofolate reductase (NAD(P)H)	EZ	-0.6781	0.206105	b
Methylglutaconyl-CoA hydratase	AZ	-1.11723	0.137139	b
Methylglutaconyl-CoA hydratase	BZ	-0.66932	0.104588	b
Methylglutaconyl-CoA hydratase	CZ	0.556537	0.109972	a
Methylglutaconyl-CoA hydratase	DZ	0.725929	0.050054	a
Methylglutaconyl-CoA hydratase	EZ	0.504085	0.237287	a
Methylisocitrate lyase	AZ	-0.90156	0.10378	b
Methylisocitrate lyase	BZ	-1.02359	0.126349	b
Methylisocitrate lyase	CZ	0.70962	0.115502	a
Methylisocitrate lyase	DZ	0.627407	0.098248	a
Methylisocitrate lyase	EZ	0.588118	0.187327	a
Methylmalonate-semialdehyde dehydrogenase (CoA acylating)	AZ	0.854874	0.07625	a
Methylmalonate-semialdehyde dehydrogenase (CoA acylating)	BZ	0.852271	0.040633	a
Methylmalonate-semialdehyde dehydrogenase (CoA acylating)	CZ	-0.27936	0.216405	b
Methylmalonate-semialdehyde dehydrogenase (CoA acylating)	DZ	-0.71674	0.113031	b
Methylmalonate-semialdehyde dehydrogenase (CoA acylating)	EZ	-0.71104	0.212608	b
Methylmalonyl-CoA epimerase	AZ	0.835083	0.040204	a
Methylmalonyl-CoA epimerase	BZ	0.883621	0.010458	a
Methylmalonyl-CoA epimerase	CZ	-0.14654	0.161629	b
Methylmalonyl-CoA epimerase	DZ	-0.73186	0.183458	b
Methylmalonyl-CoA epimerase	EZ	-0.8403	0.203534	b
Methylmalonyl-CoA mutase	AZ	0.835083	0.040204	a
Methylmalonyl-CoA mutase	BZ	0.883621	0.010458	a
Methylmalonyl-CoA mutase	CZ	-0.14654	0.161629	b
Methylmalonyl-CoA mutase	DZ	-0.73186	0.183458	b
Methylmalonyl-CoA mutase	EZ	-0.8403	0.203534	b
Mevalonate kinase	AZ	1.018767	0.030493	a
Mevalonate kinase	BZ	0.814277	0.016027	a
Mevalonate kinase	CZ	-0.62716	0.174432	b
Mevalonate kinase	DZ	-0.73398	0.141864	b
Mevalonate kinase	EZ	-0.4719	0.213254	b
Micrococcal nuclease	AZ	-0.90841	0.123829	b
Micrococcal nuclease	BZ	-0.94353	0.150456	b
Micrococcal nuclease	CZ	0.638615	0.12396	a
Micrococcal nuclease	DZ	0.709639	0.07942	a
Micrococcal nuclease	EZ	0.50368	0.190599	a
Microtubule-severing ATPase	AZ	0.879893	0.077006	a
Microtubule-severing ATPase	BZ	0.963818	0.021964	a
Microtubule-severing ATPase	CZ	-0.34996	0.167904	b
Microtubule-severing ATPase	DZ	-0.75126	0.129414	b

Microtubule-severing ATPase	EZ	-0.74249	0.203918	b
Mitochondrial intermediate peptidase	AZ	0.866625	0.102879	a
Mitochondrial intermediate peptidase	BZ	0.962814	0.033298	a
Mitochondrial intermediate peptidase	CZ	-0.38296	0.172958	b
Mitochondrial intermediate peptidase	DZ	-0.76988	0.110634	b
Mitochondrial intermediate peptidase	EZ	-0.6766	0.205894	b
Mitochondrial processing peptidase	AZ	0.846751	0.107196	a
Mitochondrial processing peptidase	BZ	0.96328	0.034766	a
Mitochondrial processing peptidase	CZ	-0.29498	0.167294	b
Mitochondrial processing peptidase	DZ	-0.85581	0.115079	b
Mitochondrial processing peptidase	EZ	-0.65924	0.202791	b
Molybdate-transporting ATPase	AZ	-0.91236	0.100228	b
Molybdate-transporting ATPase	BZ	-1.02254	0.128381	b
Molybdate-transporting ATPase	CZ	0.671024	0.113187	a
Molybdate-transporting ATPase	DZ	0.67545	0.082503	a
Molybdate-transporting ATPase	EZ	0.588425	0.193178	a
Monoamine oxidase	AZ	-0.84048	0.154415	b
Monoamine oxidase	BZ	-0.97513	0.098057	b
Monoamine oxidase	CZ	0.607599	0.11579	a
Monoamine oxidase	DZ	0.765966	0.073597	a
Monoamine oxidase	EZ	0.442045	0.217459	a
Monodehydroascorbate reductase (NADH)	AZ	-0.93671	0.152623	b
Monodehydroascorbate reductase (NADH)	BZ	-0.91874	0.094083	b
Monodehydroascorbate reductase (NADH)	CZ	0.599474	0.103553	a
Monodehydroascorbate reductase (NADH)	DZ	0.78822	0.050807	a
Monodehydroascorbate reductase (NADH)	EZ	0.467755	0.220232	a
mRNA (2'-O-methyladenosine-N(6)-)-methyltransferase	AZ	-0.89745	0.158608	b
mRNA (2'-O-methyladenosine-N(6)-)-methyltransferase	BZ	-0.88535	0.099671	b
mRNA (2'-O-methyladenosine-N(6)-)-methyltransferase	CZ	0.477909	0.185637	a
mRNA (2'-O-methyladenosine-N(6)-)-methyltransferase	DZ	0.843023	0.077156	a
mRNA (2'-O-methyladenosine-N(6)-)-methyltransferase	EZ	0.461871	0.165563	a
mRNA guanylyltransferase	AZ	-0.79721	0.116678	b
mRNA guanylyltransferase	BZ	-0.93307	0.037896	b
mRNA guanylyltransferase	CZ	0.182516	0.101772	a
mRNA guanylyltransferase	DZ	0.833982	0.17428	a
mRNA guanylyltransferase	EZ	0.71378	0.216783	a
Multiple inositol-polyphosphate phosphatase	AZ	0.835083	0.040204	a
Multiple inositol-polyphosphate phosphatase	BZ	0.883621	0.010458	a
Multiple inositol-polyphosphate phosphatase	CZ	-0.14654	0.161629	b
Multiple inositol-polyphosphate phosphatase	DZ	-0.73186	0.183458	b
Multiple inositol-polyphosphate phosphatase	EZ	-0.8403	0.203534	b
N-acetyl-gamma-glutamyl-phosphate reductase	AZ	-0.76164	0.213396	b
N-acetyl-gamma-glutamyl-phosphate reductase	BZ	-0.66518	0.143851	b
N-acetyl-gamma-glutamyl-phosphate reductase	CZ	0.792488	0.193741	a

N-acetyl-gamma-glutamyl-phosphate reductase	DZ	0.200582	0.156993	ab
N-acetyl-gamma-glutamyl-phosphate reductase	EZ	0.433754	0.132807	ab
N-acetylglucosamine-6-phosphate deacetylase	AZ	0.801763	0.062567	a
N-acetylglucosamine-6-phosphate deacetylase	BZ	0.592458	0.030359	a
N-acetylglucosamine-6-phosphate deacetylase	CZ	-0.13499	0.196349	ab
N-acetylglucosamine-6-phosphate deacetylase	DZ	-1.13024	0.213067	b
N-acetylglucosamine-6-phosphate deacetylase	EZ	-0.129	0.189491	ab
N-acetylglucosamine-6-sulfatase	AZ	-0.66959	0.245071	bc
N-acetylglucosamine-6-sulfatase	BZ	-0.90894	0.113463	c
N-acetylglucosamine-6-sulfatase	CZ	0.742316	0.157603	a
N-acetylglucosamine-6-sulfatase	DZ	0.543772	0.103486	a
N-acetylglucosamine-6-sulfatase	EZ	0.292438	0.145535	ab
N-acetylglucosaminephosphotransferase	AZ	0.866802	0.103111	a
N-acetylglucosaminephosphotransferase	BZ	0.962972	0.033353	a
N-acetylglucosaminephosphotransferase	CZ	-0.38555	0.173323	b
N-acetylglucosaminephosphotransferase	DZ	-0.76463	0.109365	b
N-acetylglucosaminephosphotransferase	EZ	-0.67959	0.206309	b
N-acetylglucosaminyldiphosphodolichol N-acetylglucosaminyltransferase	AZ	0.868417	0.101955	a
N-acetylglucosaminyldiphosphodolichol N-acetylglucosaminyltransferase	BZ	0.962537	0.033014	a
N-acetylglucosaminyldiphosphodolichol N-acetylglucosaminyltransferase	CZ	-0.38162	0.171606	b
N-acetylglucosaminyldiphosphodolichol N-acetylglucosaminyltransferase	DZ	-0.75355	0.108546	b
N-acetylglucosaminyldiphosphodolichol N-acetylglucosaminyltransferase	EZ	-0.69579	0.208264	b
N-acetyllactosaminide beta-1,3-N-acetylglucosaminyltransferase	AZ	-1.02954	0.236645	d
N-acetyllactosaminide beta-1,3-N-acetylglucosaminyltransferase	BZ	-0.67728	0.061079	cd
N-acetyllactosaminide beta-1,3-N-acetylglucosaminyltransferase	CZ	0.119054	0.083966	bc
N-acetyllactosaminide beta-1,3-N-acetylglucosaminyltransferase	DZ	1.101512	0.082576	a
N-acetyllactosaminide beta-1,3-N-acetylglucosaminyltransferase	EZ	0.486257	0.13141	ab
N-acyl-aliphatic-L-amino acid amidohydrolase	AZ	0.816366	0.04706	a
N-acyl-aliphatic-L-amino acid amidohydrolase	BZ	0.876269	0.011401	a
N-acyl-aliphatic-L-amino acid amidohydrolase	CZ	-0.21134	0.165784	b
N-acyl-aliphatic-L-amino acid amidohydrolase	DZ	-0.79318	0.192958	b
N-acyl-aliphatic-L-amino acid amidohydrolase	EZ	-0.68812	0.209423	b
N-acylneuraminate-9-phosphatase	AZ	-0.56855	0.054568	bc
N-acylneuraminate-9-phosphatase	BZ	-0.98743	0.147652	c
N-acylneuraminate-9-phosphatase	CZ	0.558435	0.029317	a
N-acylneuraminate-9-phosphatase	DZ	0.079358	0.257277	ab
N-acylneuraminate-9-phosphatase	EZ	0.918183	0.158736	a
N-carbamoylputrescine amidase	AZ	-1.0271	0.078376	b
N-carbamoylputrescine amidase	BZ	-1.0011	0.072972	b
N-carbamoylputrescine amidase	CZ	0.490014	0.037098	a
N-carbamoylputrescine amidase	DZ	0.683965	0.098256	a
N-carbamoylputrescine amidase	EZ	0.854224	0.206793	a
N-sulfoglucosamine sulfohydrolase	AZ	-0.81188	0.157477	b
N-sulfoglucosamine sulfohydrolase	BZ	-1.08815	0.078995	b

N-sulfoglucosamine sulfohydrolase	CZ	0.779984	0.117538	a
N-sulfoglucosamine sulfohydrolase	DZ	0.691439	0.062589	a
N-sulfoglucosamine sulfohydrolase	EZ	0.428605	0.188572	a
N(4)-(beta-N-acetylglucosaminyl)-L-asparaginase	AZ	-0.83303	0.176105	b
N(4)-(beta-N-acetylglucosaminyl)-L-asparaginase	BZ	-0.97862	0.078243	b
N(4)-(beta-N-acetylglucosaminyl)-L-asparaginase	CZ	0.618366	0.101897	a
N(4)-(beta-N-acetylglucosaminyl)-L-asparaginase	DZ	0.873354	0.111924	a
N(4)-(beta-N-acetylglucosaminyl)-L-asparaginase	EZ	0.319932	0.187081	a
NAD(+) ADP-ribosyltransferase	AZ	-0.91372	0.110421	b
NAD(+) ADP-ribosyltransferase	BZ	-0.95906	0.058735	b
NAD(+) ADP-ribosyltransferase	CZ	0.554511	0.154401	a
NAD(+) ADP-ribosyltransferase	DZ	0.634323	0.093814	a
NAD(+) ADP-ribosyltransferase	EZ	0.683942	0.212372	a
NAD(+) diphosphatase	AZ	0.866082	0.044425	a
NAD(+) diphosphatase	BZ	0.859355	0.020489	a
NAD(+) diphosphatase	CZ	-0.13694	0.128398	b
NAD(+) diphosphatase	DZ	-0.79892	0.187522	b
NAD(+) diphosphatase	EZ	-0.78958	0.217953	b
NAD(+) kinase	AZ	0.733598	0.115184	ab
NAD(+) kinase	BZ	0.902883	0.027059	a
NAD(+) kinase	CZ	-0.20403	0.122516	bc
NAD(+) kinase	DZ	-0.89014	0.226456	c
NAD(+) kinase	EZ	-0.54231	0.188774	c
NAD(+) synthase (glutamine-hydrolyzing)	AZ	0.866802	0.103111	a
NAD(+) synthase (glutamine-hydrolyzing)	BZ	0.962972	0.033353	a
NAD(+) synthase (glutamine-hydrolyzing)	CZ	-0.38555	0.173323	b
NAD(+) synthase (glutamine-hydrolyzing)	DZ	-0.76463	0.109365	b
NAD(+) synthase (glutamine-hydrolyzing)	EZ	-0.67959	0.206309	b
NAD(+)--protein-arginine ADP-ribosyltransferase	AZ	-0.86651	0.206282	b
NAD(+)--protein-arginine ADP-ribosyltransferase	BZ	-0.7592	0.142918	b
NAD(+)--protein-arginine ADP-ribosyltransferase	CZ	0.625923	0.187347	a
NAD(+)--protein-arginine ADP-ribosyltransferase	DZ	0.438991	0.077203	a
NAD(+)--protein-arginine ADP-ribosyltransferase	EZ	0.560799	0.157009	a
NAD(P)(+) transhydrogenase (Re/Si-specific)	AZ	-0.92507	0.164577	b
NAD(P)(+) transhydrogenase (Re/Si-specific)	BZ	-0.88877	0.094628	b
NAD(P)(+) transhydrogenase (Re/Si-specific)	CZ	0.50865	0.09345	a
NAD(P)(+) transhydrogenase (Re/Si-specific)	DZ	0.870117	0.053652	a
NAD(P)(+) transhydrogenase (Re/Si-specific)	EZ	0.435072	0.22318	a
NAD(P)H dehydrogenase (quinone)	AZ	-0.88032	0.098633	b
NAD(P)H dehydrogenase (quinone)	BZ	-1.1001	0.083981	b
NAD(P)H dehydrogenase (quinone)	CZ	0.677977	0.11875	a
NAD(P)H dehydrogenase (quinone)	DZ	0.760211	0.092001	a
NAD(P)H dehydrogenase (quinone)	EZ	0.542234	0.186585	a
NADH:ubiquinone reductase (H(+)-translocating)	AZ	0.836187	0.075227	a

NADH:ubiquinone reductase (H(+)-translocating)	BZ	0.841951	0.040927	a
NADH:ubiquinone reductase (H(+)-translocating)	CZ	-0.55247	0.225978	b
NADH:ubiquinone reductase (H(+)-translocating)	DZ	-0.37262	0.1318	b
NADH:ubiquinone reductase (H(+)-translocating)	EZ	-0.75305	0.208007	b
NADPH dehydrogenase	AZ	-0.91121	0.162861	b
NADPH dehydrogenase	BZ	-0.93977	0.093604	b
NADPH dehydrogenase	CZ	0.564586	0.114201	a
NADPH dehydrogenase	DZ	0.809704	0.041201	a
NADPH dehydrogenase	EZ	0.476696	0.210286	a
NADPH--hemoprotein reductase	AZ	-0.94068	0.117858	b
NADPH--hemoprotein reductase	BZ	-0.86382	0.07644	b
NADPH--hemoprotein reductase	CZ	0.462757	0.191943	a
NADPH--hemoprotein reductase	DZ	0.973598	0.051839	a
NADPH--hemoprotein reductase	EZ	0.368139	0.185219	a
NADPH:quinone reductase	AZ	-0.87483	0.13056	b
NADPH:quinone reductase	BZ	-0.95555	0.050705	b
NADPH:quinone reductase	CZ	0.401722	0.147572	a
NADPH:quinone reductase	DZ	0.820921	0.090068	a
NADPH:quinone reductase	EZ	0.607738	0.21527	a
Naringenin-chalcone synthase	AZ	-0.79938	0.178171	b
Naringenin-chalcone synthase	BZ	-0.94792	0.105282	b
Naringenin-chalcone synthase	CZ	0.481348	0.086194	a
Naringenin-chalcone synthase	DZ	0.817097	0.124464	a
Naringenin-chalcone synthase	EZ	0.448849	0.205748	a
Nicotianamine synthase	AZ	-0.76624	0.201062	b
Nicotianamine synthase	BZ	-0.83143	0.124281	b
Nicotianamine synthase	CZ	0.22473	0.050898	ab
Nicotianamine synthase	DZ	0.919519	0.167204	a
Nicotianamine synthase	EZ	0.453422	0.185957	a
Nicotinamidase	AZ	-0.93311	0.158265	b
Nicotinamidase	BZ	-0.89853	0.10037	b
Nicotinamidase	CZ	0.567282	0.106413	a
Nicotinamidase	DZ	0.808167	0.051339	a
Nicotinamidase	EZ	0.456191	0.218777	a
Nicotinamide N-methyltransferase	AZ	-0.93281	0.116165	b
Nicotinamide N-methyltransferase	BZ	-0.95056	0.152923	b
Nicotinamide N-methyltransferase	CZ	0.70452	0.124077	a
Nicotinamide N-methyltransferase	DZ	0.603836	0.064203	a
Nicotinamide N-methyltransferase	EZ	0.575016	0.187983	a
Nicotinamide phosphoribosyltransferase	AZ	0.835083	0.040204	a
Nicotinamide phosphoribosyltransferase	BZ	0.883621	0.010458	a
Nicotinamide phosphoribosyltransferase	CZ	-0.14654	0.161629	b
Nicotinamide phosphoribosyltransferase	DZ	-0.73186	0.183458	b
Nicotinamide phosphoribosyltransferase	EZ	-0.8403	0.203534	b

Nicotinamide-nucleotide adenylyltransferase	AZ	0.878729	0.080974	a
Nicotinamide-nucleotide adenylyltransferase	BZ	0.930567	0.031801	a
Nicotinamide-nucleotide adenylyltransferase	CZ	-0.50622	0.197665	b
Nicotinamide-nucleotide adenylyltransferase	DZ	-0.63167	0.123576	b
Nicotinamide-nucleotide adenylyltransferase	EZ	-0.6714	0.20219	b
Nicotinate-nucleotide diphosphorylase (carboxylating)	AZ	0.860117	0.108704	a
Nicotinate-nucleotide diphosphorylase (carboxylating)	BZ	0.960435	0.035561	a
Nicotinate-nucleotide diphosphorylase (carboxylating)	CZ	-0.41979	0.180996	b
Nicotinate-nucleotide diphosphorylase (carboxylating)	DZ	-0.82634	0.112576	b
Nicotinate-nucleotide diphosphorylase (carboxylating)	EZ	-0.57442	0.197289	b
Nicotinate-nucleotide--dimethylbenzimidazole phosphoribosyltransferase	AZ	-0.89137	0.161964	b
Nicotinate-nucleotide--dimethylbenzimidazole phosphoribosyltransferase	BZ	-0.83904	0.113938	b
Nicotinate-nucleotide--dimethylbenzimidazole phosphoribosyltransferase	CZ	0.501417	0.121032	a
Nicotinate-nucleotide--dimethylbenzimidazole phosphoribosyltransferase	DZ	0.690333	0.061715	a
Nicotinate-nucleotide--dimethylbenzimidazole phosphoribosyltransferase	EZ	0.538661	0.237687	a
Nitrilase	AZ	-0.88132	0.160783	b
Nitrilase	BZ	-0.91248	0.098793	b
Nitrilase	CZ	0.567339	0.11131	a
Nitrilase	DZ	0.761441	0.065842	a
Nitrilase	EZ	0.465021	0.226497	a
Non-reducing end alpha-L-arabinofuranosidase	AZ	-0.91414	0.157092	b
Non-reducing end alpha-L-arabinofuranosidase	BZ	-0.90779	0.092949	b
Non-reducing end alpha-L-arabinofuranosidase	CZ	0.590031	0.10059	a
Non-reducing end alpha-L-arabinofuranosidase	DZ	0.782694	0.083307	a
Non-reducing end alpha-L-arabinofuranosidase	EZ	0.449204	0.220252	a
Nucleoside-diphosphate kinase	AZ	0.868975	0.073334	a
Nucleoside-diphosphate kinase	BZ	0.951917	0.020095	a
Nucleoside-diphosphate kinase	CZ	-0.25343	0.156533	b
Nucleoside-diphosphate kinase	DZ	-0.80808	0.142968	b
Nucleoside-diphosphate kinase	EZ	-0.75939	0.20376	b
Nucleoside-triphosphate diphosphatase	AZ	0.803114	0.08275	a
Nucleoside-triphosphate diphosphatase	BZ	0.87983	0.026474	a
Nucleoside-triphosphate diphosphatase	CZ	0.042854	0.094703	ab
Nucleoside-triphosphate diphosphatase	DZ	-0.91859	0.171519	c
Nucleoside-triphosphate diphosphatase	EZ	-0.80721	0.219655	bc
Nucleoside-triphosphate phosphatase	AZ	-0.93034	0.163708	b
Nucleoside-triphosphate phosphatase	BZ	-0.84634	0.100899	b
Nucleoside-triphosphate phosphatase	CZ	0.49594	0.092128	a
Nucleoside-triphosphate phosphatase	DZ	0.816902	0.079034	a
Nucleoside-triphosphate phosphatase	EZ	0.463833	0.229724	a
o-pyrocatechuate decarboxylase	AZ	-0.90332	0.084559	b
o-pyrocatechuate decarboxylase	BZ	-1.14563	0.063606	b
o-pyrocatechuate decarboxylase	CZ	0.740765	0.092797	a
o-pyrocatechuate decarboxylase	DZ	0.615915	0.0853	a

o-pyrocatechuate decarboxylase	EZ	0.692261	0.190077	a
Oleate hydratase	AZ	-0.81857	0.083135	b
Oleate hydratase	BZ	-1.204	0.060222	b
Oleate hydratase	CZ	0.768072	0.113489	a
Oleate hydratase	DZ	0.65262	0.106526	a
Oleate hydratase	EZ	0.601875	0.174201	a
Oligo-1,6-glucosidase	AZ	-0.99026	0.150666	b
Oligo-1,6-glucosidase	BZ	-0.25634	0.208797	ab
Oligo-1,6-glucosidase	CZ	0.302174	0.18971	a
Oligo-1,6-glucosidase	DZ	0.800486	0.11857	a
Oligo-1,6-glucosidase	EZ	0.143942	0.18357	ab
Oligopeptidase B	AZ	0.835083	0.040204	a
Oligopeptidase B	BZ	0.883621	0.010458	a
Oligopeptidase B	CZ	-0.14654	0.161629	b
Oligopeptidase B	DZ	-0.73186	0.183458	b
Oligopeptidase B	EZ	-0.8403	0.203534	b
Omega-amidase	AZ	-0.91815	0.130856	b
Omega-amidase	BZ	-0.88248	0.183847	b
Omega-amidase	CZ	0.633049	0.096675	a
Omega-amidase	DZ	0.527191	0.077845	a
Omega-amidase	EZ	0.640396	0.195302	a
Ornithine aminotransferase	AZ	0.566457	0.074956	a
Ornithine aminotransferase	BZ	0.798427	0.044119	a
Ornithine aminotransferase	CZ	0.350495	0.148538	a
Ornithine aminotransferase	DZ	-1.01451	0.214746	b
Ornithine aminotransferase	EZ	-0.70087	0.184859	b
Ornithine carbamoyltransferase	AZ	0.866802	0.103111	a
Ornithine carbamoyltransferase	BZ	0.962972	0.033353	a
Ornithine carbamoyltransferase	CZ	-0.38555	0.173323	b
Ornithine carbamoyltransferase	DZ	-0.76463	0.109365	b
Ornithine carbamoyltransferase	EZ	-0.67959	0.206309	b
Ornithine cyclodeaminase	AZ	-0.83465	0.16958	b
Ornithine cyclodeaminase	BZ	-0.92386	0.104238	b
Ornithine cyclodeaminase	CZ	0.603803	0.147263	a
Ornithine cyclodeaminase	DZ	0.763103	0.041511	a
Ornithine cyclodeaminase	EZ	0.3916	0.210825	a
Orotate phosphoribosyltransferase	AZ	0.869003	0.101853	a
Orotate phosphoribosyltransferase	BZ	0.961099	0.033099	a
Orotate phosphoribosyltransferase	CZ	-0.37196	0.17163	b
Orotate phosphoribosyltransferase	DZ	-0.78328	0.111643	b
Orotate phosphoribosyltransferase	EZ	-0.67486	0.205669	b
Orotidine-5'-phosphate decarboxylase	AZ	0.717664	0.097725	ab
Orotidine-5'-phosphate decarboxylase	BZ	0.891248	0.023669	a
Orotidine-5'-phosphate decarboxylase	CZ	-0.26464	0.168063	bc

Orotidine-5'-phosphate decarboxylase	DZ	-0.69127	0.212079	c
Orotidine-5'-phosphate decarboxylase	EZ	-0.65299	0.206116	c
Oxoglutarate dehydrogenase (succinyl-transferring)	AZ	0.861154	0.089157	a
Oxoglutarate dehydrogenase (succinyl-transferring)	BZ	0.946983	0.027374	a
Oxoglutarate dehydrogenase (succinyl-transferring)	CZ	-0.29093	0.163403	b
Oxoglutarate dehydrogenase (succinyl-transferring)	DZ	-0.78173	0.133704	b
Oxoglutarate dehydrogenase (succinyl-transferring)	EZ	-0.73548	0.207298	b
Palmitoyl-CoA hydrolase	AZ	0.835529	0.043348	a
Palmitoyl-CoA hydrolase	BZ	0.785056	0.044792	a
Palmitoyl-CoA hydrolase	CZ	-0.57772	0.207349	b
Palmitoyl-CoA hydrolase	DZ	-0.81425	0.232971	b
Palmitoyl-CoA hydrolase	EZ	-0.22861	0.14378	ab
Palmitoyl-protein hydrolase	AZ	0.878595	0.103414	a
Palmitoyl-protein hydrolase	BZ	0.940052	0.040484	a
Palmitoyl-protein hydrolase	CZ	-0.37419	0.173478	b
Palmitoyl-protein hydrolase	DZ	-0.75436	0.109297	b
Palmitoyl-protein hydrolase	EZ	-0.6901	0.208757	b
Pantetheine-phosphate adenylyltransferase	AZ	-0.8949	0.175888	b
Pantetheine-phosphate adenylyltransferase	BZ	-0.92275	0.081658	b
Pantetheine-phosphate adenylyltransferase	CZ	0.563471	0.122989	a
Pantetheine-phosphate adenylyltransferase	DZ	0.788573	0.147729	a
Pantetheine-phosphate adenylyltransferase	EZ	0.465609	0.158184	a
Pantoate--beta-alanine ligase (AMP-forming)	AZ	0.866802	0.103111	a
Pantoate--beta-alanine ligase (AMP-forming)	BZ	0.962972	0.033353	a
Pantoate--beta-alanine ligase (AMP-forming)	CZ	-0.38555	0.173323	b
Pantoate--beta-alanine ligase (AMP-forming)	DZ	-0.76463	0.109365	b
Pantoate--beta-alanine ligase (AMP-forming)	EZ	-0.67959	0.206309	b
Pantothenate kinase	AZ	0.876679	0.090698	a
Pantothenate kinase	BZ	0.984317	0.028123	a
Pantothenate kinase	CZ	-0.45752	0.163466	b
Pantothenate kinase	DZ	-0.744	0.132625	b
Pantothenate kinase	EZ	-0.65947	0.199779	b
Pectate lyase	AZ	-0.85325	0.121212	b
Pectate lyase	BZ	-1.12128	0.044211	b
Pectate lyase	CZ	0.72192	0.121058	a
Pectate lyase	DZ	0.660688	0.100523	a
Pectate lyase	EZ	0.591927	0.184187	a
Pectin lyase	AZ	-0.85084	0.089212	b
Pectin lyase	BZ	-1.17283	0.053363	b
Pectin lyase	CZ	0.744517	0.114432	a
Pectin lyase	DZ	0.664626	0.100056	a
Pectin lyase	EZ	0.614519	0.18009	a
Pectinesterase	AZ	-1.02906	0.143462	b
Pectinesterase	BZ	-0.86816	0.071201	b

Pectinesterase	CZ	0.555381	0.107399	a
Pectinesterase	DZ	0.927744	0.051064	a
Pectinesterase	EZ	0.414089	0.206259	a
Pepsin A	AZ	-0.94725	0.190344	b
Pepsin A	BZ	-0.87214	0.066234	b
Pepsin A	CZ	0.460844	0.138494	a
Pepsin A	DZ	0.913952	0.09582	a
Pepsin A	EZ	0.444593	0.160802	a
Peptide alpha-N-acetyltransferase	AZ	-0.82843	0.019853	b
Peptide alpha-N-acetyltransferase	BZ	-0.74934	0.117252	b
Peptide alpha-N-acetyltransferase	CZ	0.682107	0.240302	a
Peptide alpha-N-acetyltransferase	DZ	0.636891	0.202254	a
Peptide alpha-N-acetyltransferase	EZ	0.258777	0.133874	ab
Peptide-N(4)-(N-acetyl-beta-glucosaminyl)asparagine amidase	AZ	-0.81841	0.165027	b
Peptide-N(4)-(N-acetyl-beta-glucosaminyl)asparagine amidase	BZ	-0.94357	0.117161	b
Peptide-N(4)-(N-acetyl-beta-glucosaminyl)asparagine amidase	CZ	0.554784	0.075302	a
Peptide-N(4)-(N-acetyl-beta-glucosaminyl)asparagine amidase	DZ	0.743188	0.101957	a
Peptide-N(4)-(N-acetyl-beta-glucosaminyl)asparagine amidase	EZ	0.464003	0.226269	a
Peptidylprolyl isomerase	AZ	0.884439	0.091169	a
Peptidylprolyl isomerase	BZ	0.970379	0.029634	a
Peptidylprolyl isomerase	CZ	-0.36485	0.161255	b
Peptidylprolyl isomerase	DZ	-0.79435	0.119368	b
Peptidylprolyl isomerase	EZ	-0.69562	0.204853	b
Peroxidase	AZ	-0.91558	0.165896	b
Peroxidase	BZ	-0.83131	0.104741	b
Peroxidase	CZ	0.533872	0.104321	a
Peroxidase	DZ	0.737251	0.071025	a
Peroxidase	EZ	0.475765	0.237417	a
Persulfide dioxygenase	AZ	-0.88123	0.086574	b
Persulfide dioxygenase	BZ	-1.15649	0.059691	b
Persulfide dioxygenase	CZ	0.734481	0.103584	a
Persulfide dioxygenase	DZ	0.64058	0.09162	a
Persulfide dioxygenase	EZ	0.662659	0.1862	a
Phenol 2-monooxygenase	AZ	-0.96618	0.163152	b
Phenol 2-monooxygenase	BZ	-0.84009	0.094829	b
Phenol 2-monooxygenase	CZ	0.531597	0.106045	a
Phenol 2-monooxygenase	DZ	0.82865	0.044663	a
Phenol 2-monooxygenase	EZ	0.44602	0.225337	a
Phenylalanine 4-monooxygenase	AZ	0.835083	0.040204	a
Phenylalanine 4-monooxygenase	BZ	0.883621	0.010458	a
Phenylalanine 4-monooxygenase	CZ	-0.14654	0.161629	b
Phenylalanine 4-monooxygenase	DZ	-0.73186	0.183458	b
Phenylalanine 4-monooxygenase	EZ	-0.8403	0.203534	b
Phenylalanine--tRNA ligase	AZ	0.866801	0.103111	a

Phenylalanine--tRNA ligase	BZ	0.962972	0.033353	a
Phenylalanine--tRNA ligase	CZ	-0.38555	0.173323	b
Phenylalanine--tRNA ligase	DZ	-0.76463	0.109365	b
Phenylalanine--tRNA ligase	EZ	-0.67959	0.206309	b
Phosphate-transporting ATPase	AZ	-0.79162	0.103435	b
Phosphate-transporting ATPase	BZ	-1.15577	0.057948	b
Phosphate-transporting ATPase	CZ	0.789922	0.133689	a
Phosphate-transporting ATPase	DZ	0.702334	0.118265	a
Phosphate-transporting ATPase	EZ	0.455133	0.172454	a
Phosphatidate cytidylyltransferase	AZ	0.866802	0.103111	a
Phosphatidate cytidylyltransferase	BZ	0.962972	0.033353	a
Phosphatidate cytidylyltransferase	CZ	-0.38555	0.173323	b
Phosphatidate cytidylyltransferase	DZ	-0.76463	0.109365	b
Phosphatidate cytidylyltransferase	EZ	-0.67959	0.206309	b
Phosphatidate phosphatase	AZ	0.897831	0.078575	a
Phosphatidate phosphatase	BZ	0.968147	0.025799	a
Phosphatidate phosphatase	CZ	-0.54933	0.186643	b
Phosphatidate phosphatase	DZ	-0.77783	0.124558	b
Phosphatidate phosphatase	EZ	-0.53881	0.189168	b
Phosphatidyl-N-methylethanolamine N-methyltransferase	AZ	-1.06871	0.232637	b
Phosphatidyl-N-methylethanolamine N-methyltransferase	BZ	-0.73775	0.066113	b
Phosphatidyl-N-methylethanolamine N-methyltransferase	CZ	0.389591	0.084582	a
Phosphatidyl-N-methylethanolamine N-methyltransferase	DZ	0.984553	0.081332	a
Phosphatidyl-N-methylethanolamine N-methyltransferase	EZ	0.432316	0.134232	a
Phosphatidylethanolamine N-methyltransferase	AZ	0.866802	0.103111	a
Phosphatidylethanolamine N-methyltransferase	BZ	0.962972	0.033353	a
Phosphatidylethanolamine N-methyltransferase	CZ	-0.38555	0.173323	b
Phosphatidylethanolamine N-methyltransferase	DZ	-0.76463	0.109365	b
Phosphatidylethanolamine N-methyltransferase	EZ	-0.67959	0.206309	b
Phosphatidylglycerophosphatase	AZ	-0.87445	0.120752	b
Phosphatidylglycerophosphatase	BZ	-0.93992	0.172271	b
Phosphatidylglycerophosphatase	CZ	0.651346	0.124801	a
Phosphatidylglycerophosphatase	DZ	0.61008	0.097015	a
Phosphatidylglycerophosphatase	EZ	0.552945	0.182316	a
Phosphatidylinositol 3-kinase	AZ	0.887953	0.087037	a
Phosphatidylinositol 3-kinase	BZ	0.969985	0.027559	a
Phosphatidylinositol 3-kinase	CZ	-0.44839	0.175852	b
Phosphatidylinositol 3-kinase	DZ	-0.72981	0.114308	b
Phosphatidylinositol 3-kinase	EZ	-0.67973	0.203525	b
Phosphatidylinositol N-acetylglucosaminyltransferase	AZ	0.889451	0.081837	a
Phosphatidylinositol N-acetylglucosaminyltransferase	BZ	0.978848	0.025976	a
Phosphatidylinositol N-acetylglucosaminyltransferase	CZ	-0.42563	0.173234	b
Phosphatidylinositol N-acetylglucosaminyltransferase	DZ	-0.7732	0.118205	b
Phosphatidylinositol N-acetylglucosaminyltransferase	EZ	-0.66947	0.199556	b

Phosphatidylserine decarboxylase	AZ	-0.79005	0.069909	b
Phosphatidylserine decarboxylase	BZ	-0.88011	0.035676	b
Phosphatidylserine decarboxylase	CZ	0.263634	0.223118	a
Phosphatidylserine decarboxylase	DZ	0.861713	0.163524	a
Phosphatidylserine decarboxylase	EZ	0.544818	0.178704	a
Phosphoacetylglucosamine mutase	AZ	0.866694	0.102883	a
Phosphoacetylglucosamine mutase	BZ	0.962712	0.03327	a
Phosphoacetylglucosamine mutase	CZ	-0.38294	0.172965	b
Phosphoacetylglucosamine mutase	DZ	-0.76987	0.110638	b
Phosphoacetylglucosamine mutase	EZ	-0.6766	0.205902	b
Phosphodiesterase I	AZ	-0.70639	0.03206	bc
Phosphodiesterase I	BZ	-0.79137	0.035701	c
Phosphodiesterase I	CZ	0.219899	0.238333	abc
Phosphodiesterase I	DZ	0.895632	0.232956	a
Phosphodiesterase I	EZ	0.382228	0.143295	ab
Phosphoenolpyruvate carboxykinase (ATP)	AZ	0.866659	0.102881	a
Phosphoenolpyruvate carboxykinase (ATP)	BZ	0.962763	0.033284	a
Phosphoenolpyruvate carboxykinase (ATP)	CZ	-0.38295	0.172962	b
Phosphoenolpyruvate carboxykinase (ATP)	DZ	-0.76987	0.110636	b
Phosphoenolpyruvate carboxykinase (ATP)	EZ	-0.6766	0.205898	b
Phosphoenolpyruvate mutase	AZ	-0.82559	0.203332	b
Phosphoenolpyruvate mutase	BZ	-0.7258	0.140931	b
Phosphoenolpyruvate mutase	CZ	0.483151	0.220533	a
Phosphoenolpyruvate mutase	DZ	0.485257	0.075736	a
Phosphoenolpyruvate mutase	EZ	0.582975	0.153514	a
Phosphoethanolamine N-methyltransferase	AZ	-0.61232	0.084784	b
Phosphoethanolamine N-methyltransferase	BZ	-1.32059	0.139203	b
Phosphoethanolamine N-methyltransferase	CZ	0.710696	0.107787	a
Phosphoethanolamine N-methyltransferase	DZ	0.604545	0.1063	a
Phosphoethanolamine N-methyltransferase	EZ	0.617669	0.148215	a
Phosphoglucomutase (alpha-D-glucose-1,6-bisphosphate-dependent)	AZ	0.82414	0.10687	a
Phosphoglucomutase (alpha-D-glucose-1,6-bisphosphate-dependent)	BZ	0.923816	0.034569	a
Phosphoglucomutase (alpha-D-glucose-1,6-bisphosphate-dependent)	CZ	-0.146	0.169765	b
Phosphoglucomutase (alpha-D-glucose-1,6-bisphosphate-dependent)	DZ	-0.84631	0.110887	b
Phosphoglucomutase (alpha-D-glucose-1,6-bisphosphate-dependent)	EZ	-0.75565	0.210255	b
Phosphoglycerate dehydrogenase	AZ	-0.80194	0.145618	b
Phosphoglycerate dehydrogenase	BZ	-0.9934	0.054112	b
Phosphoglycerate dehydrogenase	CZ	0.542293	0.150345	a
Phosphoglycerate dehydrogenase	DZ	0.587052	0.108454	a
Phosphoglycerate dehydrogenase	EZ	0.665993	0.214223	a
Phosphoglycerate kinase	AZ	0.866836	0.103113	a
Phosphoglycerate kinase	BZ	0.96292	0.033339	a
Phosphoglycerate kinase	CZ	-0.38554	0.173326	b
Phosphoglycerate kinase	DZ	-0.76463	0.109367	b

Phosphoglycerate kinase	EZ	-0.67959	0.206313	b
Phosphoglycolate phosphatase	AZ	0.84509	0.103869	a
Phosphoglycolate phosphatase	BZ	0.945677	0.029362	a
Phosphoglycolate phosphatase	CZ	-0.31896	0.150613	b
Phosphoglycolate phosphatase	DZ	-0.89365	0.159259	b
Phosphoglycolate phosphatase	EZ	-0.57816	0.195311	b
Phosphoinositide 5-phosphatase	AZ	-0.83199	0.092479	b
Phosphoinositide 5-phosphatase	BZ	-0.9807	0.044291	b
Phosphoinositide 5-phosphatase	CZ	0.48724	0.216425	a
Phosphoinositide 5-phosphatase	DZ	0.733177	0.085615	a
Phosphoinositide 5-phosphatase	EZ	0.592269	0.190606	a
Phosphoinositide phospholipase C	AZ	-0.80922	0.129003	b
Phosphoinositide phospholipase C	BZ	-0.973	0.043158	b
Phosphoinositide phospholipase C	CZ	0.479028	0.149679	a
Phosphoinositide phospholipase C	DZ	0.731959	0.160367	a
Phosphoinositide phospholipase C	EZ	0.57123	0.196772	a
Phospholipase A(2)	AZ	-0.91658	0.138274	b
Phospholipase A(2)	BZ	-0.87443	0.17501	b
Phospholipase A(2)	CZ	0.581778	0.121809	a
Phospholipase A(2)	DZ	0.747161	0.073463	a
Phospholipase A(2)	EZ	0.462069	0.185119	a
Phospholipase C	AZ	-0.85632	0.086937	b
Phospholipase C	BZ	-1.17695	0.055952	b
Phospholipase C	CZ	0.7418	0.109988	a
Phospholipase C	DZ	0.655793	0.09683	a
Phospholipase C	EZ	0.635671	0.180813	a
Phospholipase D	AZ	-0.69548	0.062349	c
Phospholipase D	BZ	-0.79598	0.035636	c
Phospholipase D	CZ	-0.07529	0.038277	bc
Phospholipase D	DZ	1.143378	0.280262	a
Phospholipase D	EZ	0.423376	0.156063	ab
Phospholipid-translocating ATPase	AZ	0.881992	0.090283	a
Phospholipid-translocating ATPase	BZ	0.9729	0.026874	a
Phospholipid-translocating ATPase	CZ	-0.38137	0.16568	b
Phospholipid-translocating ATPase	DZ	-0.74116	0.114001	b
Phospholipid-translocating ATPase	EZ	-0.73237	0.207129	b
Phosphomannomutase	AZ	0.8371	0.085447	a
Phosphomannomutase	BZ	0.928181	0.024289	a
Phosphomannomutase	CZ	-0.27402	0.143883	b
Phosphomannomutase	DZ	-0.94822	0.185613	b
Phosphomannomutase	EZ	-0.54304	0.188113	b
Phosphomethylpyrimidine kinase	AZ	0.812489	0.079898	a
Phosphomethylpyrimidine kinase	BZ	0.923037	0.021656	a
Phosphomethylpyrimidine kinase	CZ	-0.17702	0.132805	b

Phosphomethylpyrimidine kinase	DZ	-0.62302	0.160477	b
Phosphomethylpyrimidine kinase	EZ	-0.93549	0.222304	b
Phosphomevalonate kinase	AZ	0.866802	0.103111	a
Phosphomevalonate kinase	BZ	0.962972	0.033353	a
Phosphomevalonate kinase	CZ	-0.38555	0.173323	b
Phosphomevalonate kinase	DZ	-0.76463	0.109365	b
Phosphomevalonate kinase	EZ	-0.67959	0.206309	b
Phosphopantothenate--cysteine ligase	AZ	0.867003	0.102901	a
Phosphopantothenate--cysteine ligase	BZ	0.962252	0.033146	a
Phosphopantothenate--cysteine ligase	CZ	-0.38285	0.172995	b
Phosphopantothenate--cysteine ligase	DZ	-0.76985	0.110658	b
Phosphopantothenate--cysteine ligase	EZ	-0.67656	0.205938	b
Phosphopantothenoylcysteine decarboxylase	AZ	0.817174	0.11207	a
Phosphopantothenoylcysteine decarboxylase	BZ	0.918005	0.036871	a
Phosphopantothenoylcysteine decarboxylase	CZ	-0.14709	0.175339	b
Phosphopantothenoylcysteine decarboxylase	DZ	-0.93479	0.116632	b
Phosphopantothenoylcysteine decarboxylase	EZ	-0.6533	0.200239	b
Phosphopyruvate hydratase	AZ	0.859015	0.111385	a
Phosphopyruvate hydratase	BZ	0.957644	0.03672	a
Phosphopyruvate hydratase	CZ	-0.43721	0.187281	b
Phosphopyruvate hydratase	DZ	-0.84344	0.112283	b
Phosphopyruvate hydratase	EZ	-0.53601	0.190583	b
Phosphoribosylamine--glycine ligase	AZ	-0.75153	0.195465	b
Phosphoribosylamine--glycine ligase	BZ	-0.8319	0.129066	b
Phosphoribosylamine--glycine ligase	CZ	0.619984	0.108694	a
Phosphoribosylamine--glycine ligase	DZ	0.434852	0.113952	a
Phosphoribosylamine--glycine ligase	EZ	0.52859	0.234199	a
Phosphoribosylaminoimidazole carboxylase	AZ	0.867146	0.103132	a
Phosphoribosylaminoimidazole carboxylase	BZ	0.96246	0.033215	a
Phosphoribosylaminoimidazole carboxylase	CZ	-0.38545	0.173357	b
Phosphoribosylaminoimidazole carboxylase	DZ	-0.7646	0.109387	b
Phosphoribosylaminoimidazole carboxylase	EZ	-0.67955	0.206349	b
Phosphoribosylaminoimidazolesuccinocarboxamide synthase	AZ	0.866802	0.103111	a
Phosphoribosylaminoimidazolesuccinocarboxamide synthase	BZ	0.962972	0.033353	a
Phosphoribosylaminoimidazolesuccinocarboxamide synthase	CZ	-0.38555	0.173323	b
Phosphoribosylaminoimidazolesuccinocarboxamide synthase	DZ	-0.76463	0.109365	b
Phosphoribosylaminoimidazolesuccinocarboxamide synthase	EZ	-0.67959	0.206309	b
Phosphoribosylformylglycinamidine synthase	AZ	0.866802	0.103111	a
Phosphoribosylformylglycinamidine synthase	BZ	0.962972	0.033353	a
Phosphoribosylformylglycinamidine synthase	CZ	-0.38555	0.173323	b
Phosphoribosylformylglycinamidine synthase	DZ	-0.76463	0.109365	b
Phosphoribosylformylglycinamidine synthase	EZ	-0.67959	0.206309	b
Phosphoribosylglycinamide formyltransferase	AZ	0.868645	0.101496	a
Phosphoribosylglycinamide formyltransferase	BZ	0.960563	0.032988	a

Phosphoribosylglycinamide formyltransferase	CZ	-0.3711	0.171907	b
Phosphoribosylglycinamide formyltransferase	DZ	-0.78655	0.112839	b
Phosphoribosylglycinamide formyltransferase	EZ	-0.67155	0.205174	b
Phosphoserine phosphatase	AZ	0.855235	0.072045	a
Phosphoserine phosphatase	BZ	0.914577	0.024572	a
Phosphoserine phosphatase	CZ	-0.59987	0.242775	b
Phosphoserine phosphatase	DZ	-0.69761	0.072332	b
Phosphoserine phosphatase	EZ	-0.47234	0.195347	b
Phosphoserine transaminase	AZ	0.866802	0.103111	a
Phosphoserine transaminase	BZ	0.962972	0.033353	a
Phosphoserine transaminase	CZ	-0.38555	0.173323	b
Phosphoserine transaminase	DZ	-0.76463	0.109365	b
Phosphoserine transaminase	EZ	-0.67959	0.206309	b
Phytanoyl-CoA dioxygenase	AZ	-0.92849	0.112252	b
Phytanoyl-CoA dioxygenase	BZ	-0.97052	0.149811	b
Phytanoyl-CoA dioxygenase	CZ	0.656261	0.101742	a
Phytanoyl-CoA dioxygenase	DZ	0.628795	0.071513	a
Phytanoyl-CoA dioxygenase	EZ	0.613951	0.197841	a
Phytepsin	AZ	-1.10532	0.122377	c
Phytepsin	BZ	-0.49483	0.108981	bc
Phytepsin	CZ	0.503281	0.062782	ab
Phytepsin	DZ	0.688895	0.169774	a
Phytepsin	EZ	0.407976	0.249674	ab
Poly(A)-specific ribonuclease	AZ	0.843143	0.09614	a
Poly(A)-specific ribonuclease	BZ	0.933579	0.026702	a
Poly(A)-specific ribonuclease	CZ	-0.64361	0.193105	b
Poly(A)-specific ribonuclease	DZ	-0.7132	0.128611	b
Poly(A)-specific ribonuclease	EZ	-0.41991	0.205256	b
Poly(ADP-ribose) glycohydrolase	AZ	-0.91835	0.095926	b
Poly(ADP-ribose) glycohydrolase	BZ	-1.08963	0.074358	b
Poly(ADP-ribose) glycohydrolase	CZ	0.747501	0.100282	a
Poly(ADP-ribose) glycohydrolase	DZ	0.619499	0.087865	a
Poly(ADP-ribose) glycohydrolase	EZ	0.640976	0.195679	a
Polyamine-transporting ATPase	AZ	-0.58224	0.05391	bc
Polyamine-transporting ATPase	BZ	-0.99607	0.145874	c
Polyamine-transporting ATPase	CZ	0.626634	0.015947	a
Polyamine-transporting ATPase	DZ	0.062719	0.254967	ab
Polyamine-transporting ATPase	EZ	0.888953	0.15701	a
Polygalacturonase	AZ	-0.95977	0.161458	b
Polygalacturonase	BZ	-0.81485	0.095112	b
Polygalacturonase	CZ	0.50564	0.112035	a
Polygalacturonase	DZ	0.816801	0.032209	a
Polygalacturonase	EZ	0.452179	0.235953	a
Polynucleotide 5'-hydroxyl-kinase	AZ	-0.91474	0.116984	b

Polynucleotide 5'-hydroxyl-kinase	BZ	-0.95351	0.1432	b
Polynucleotide 5'-hydroxyl-kinase	CZ	0.65631	0.125821	a
Polynucleotide 5'-hydroxyl-kinase	DZ	0.727624	0.084706	a
Polynucleotide 5'-hydroxyl-kinase	EZ	0.484312	0.189128	a
Polypeptide N-acetylgalactosaminyltransferase	AZ	-0.86922	0.121242	b
Polypeptide N-acetylgalactosaminyltransferase	BZ	-0.93495	0.17297	b
Polypeptide N-acetylgalactosaminyltransferase	CZ	0.626198	0.129524	a
Polypeptide N-acetylgalactosaminyltransferase	DZ	0.618622	0.09747	a
Polypeptide N-acetylgalactosaminyltransferase	EZ	0.559346	0.182312	a
Polyprenyldihydroxybenzoate methyltransferase	AZ	-0.89082	0.163787	b
Polyprenyldihydroxybenzoate methyltransferase	BZ	-0.85791	0.105542	b
Polyprenyldihydroxybenzoate methyltransferase	CZ	0.462163	0.094984	a
Polyprenyldihydroxybenzoate methyltransferase	DZ	0.781342	0.092227	a
Polyprenyldihydroxybenzoate methyltransferase	EZ	0.50522	0.232512	a
Polyribonucleotide nucleotidyltransferase	AZ	0.856247	0.081918	a
Polyribonucleotide nucleotidyltransferase	BZ	0.919564	0.029109	a
Polyribonucleotide nucleotidyltransferase	CZ	-0.45978	0.20717	b
Polyribonucleotide nucleotidyltransferase	DZ	-0.68297	0.12581	b
Polyribonucleotide nucleotidyltransferase	EZ	-0.63307	0.202054	b
Prephenate dehydratase	AZ	-0.92901	0.113137	b
Prephenate dehydratase	BZ	-0.91189	0.189148	b
Prephenate dehydratase	CZ	0.642822	0.098106	a
Prephenate dehydratase	DZ	0.542419	0.082292	a
Prephenate dehydratase	EZ	0.65566	0.182838	a
Prephenate dehydrogenase (NADP(+))	AZ	0.866836	0.103113	a
Prephenate dehydrogenase (NADP(+))	BZ	0.96292	0.033339	a
Prephenate dehydrogenase (NADP(+))	CZ	-0.38554	0.173326	b
Prephenate dehydrogenase (NADP(+))	DZ	-0.76463	0.109367	b
Prephenate dehydrogenase (NADP(+))	EZ	-0.67959	0.206313	b
Procollagen galactosyltransferase	AZ	-0.51189	0.051914	bc
Procollagen galactosyltransferase	BZ	-0.96978	0.163971	c
Procollagen galactosyltransferase	CZ	0.552643	0.027522	ab
Procollagen galactosyltransferase	DZ	0.04002	0.269057	abc
Procollagen galactosyltransferase	EZ	0.889012	0.14925	a
Procollagen-proline dioxygenase	AZ	-0.91592	0.158521	b
Procollagen-proline dioxygenase	BZ	-0.87684	0.103569	b
Procollagen-proline dioxygenase	CZ	0.582176	0.100116	a
Procollagen-proline dioxygenase	DZ	0.73485	0.068415	a
Procollagen-proline dioxygenase	EZ	0.475737	0.231926	a
Proline--tRNA ligase	AZ	0.867814	0.100902	a
Proline--tRNA ligase	BZ	0.969028	0.02764	a
Proline--tRNA ligase	CZ	-0.58046	0.213504	b
Proline--tRNA ligase	DZ	-0.74664	0.084163	b
Proline--tRNA ligase	EZ	-0.50974	0.18458	b

Prolyl aminopeptidase	AZ	0.800114	0.121641	a
Prolyl aminopeptidase	BZ	0.83743	0.054823	a
Prolyl aminopeptidase	CZ	0.177942	0.084244	ab
Prolyl aminopeptidase	DZ	-1.18079	0.104469	c
Prolyl aminopeptidase	EZ	-0.6347	0.213718	bc
Prolyl oligopeptidase	AZ	0.825767	0.059261	a
Prolyl oligopeptidase	BZ	0.892691	0.016936	a
Prolyl oligopeptidase	CZ	-0.11604	0.154034	b
Prolyl oligopeptidase	DZ	-0.74667	0.166498	b
Prolyl oligopeptidase	EZ	-0.85575	0.213657	b
Propionate--CoA ligase	AZ	-0.96261	0.15911	b
Propionate--CoA ligase	BZ	-0.82215	0.099357	b
Propionate--CoA ligase	CZ	0.521479	0.100093	a
Propionate--CoA ligase	DZ	0.811744	0.064369	a
Propionate--CoA ligase	EZ	0.45153	0.231918	a
Propionyl-CoA carboxylase	AZ	0.848766	0.064828	a
Propionyl-CoA carboxylase	BZ	0.923182	0.018483	a
Propionyl-CoA carboxylase	CZ	-0.45468	0.206712	b
Propionyl-CoA carboxylase	DZ	-0.73519	0.16656	b
Propionyl-CoA carboxylase	EZ	-0.58208	0.179018	b
Proprotein convertase 2	AZ	-1.02877	0.15774	b
Proprotein convertase 2	BZ	-0.22402	0.205929	ab
Proprotein convertase 2	CZ	0.315487	0.184408	a
Proprotein convertase 2	DZ	0.814878	0.11712	a
Proprotein convertase 2	EZ	0.12243	0.175021	ab
Prostaglandin-D synthase	AZ	-0.72824	0.069858	b
Prostaglandin-D synthase	BZ	-0.77055	0.071882	b
Prostaglandin-D synthase	CZ	0.617972	0.033574	a
Prostaglandin-D synthase	DZ	-0.03072	0.280921	ab
Prostaglandin-D synthase	EZ	0.911544	0.181706	a
Prostaglandin-E synthase	AZ	-0.90959	0.121271	b
Prostaglandin-E synthase	BZ	-0.91939	0.166399	b
Prostaglandin-E synthase	CZ	0.669095	0.113269	a
Prostaglandin-E synthase	DZ	0.584316	0.092462	a
Prostaglandin-E synthase	EZ	0.575561	0.191247	a
Prostaglandin-endoperoxide synthase	AZ	-0.878	0.04665	b
Prostaglandin-endoperoxide synthase	BZ	-0.50282	0.129332	b
Prostaglandin-endoperoxide synthase	CZ	-0.10696	0.264749	ab
Prostaglandin-endoperoxide synthase	DZ	0.689759	0.071996	a
Prostaglandin-endoperoxide synthase	EZ	0.798026	0.196043	a
Proteasome endopeptidase complex	AZ	0.875182	0.092506	a
Proteasome endopeptidase complex	BZ	0.953193	0.031192	a
Proteasome endopeptidase complex	CZ	-0.3694	0.17223	b
Proteasome endopeptidase complex	DZ	-0.75	0.118754	b

Proteasome endopeptidase complex	EZ	-0.70897	0.207118	b
Protein C (activated)	AZ	-0.56855	0.054568	bc
Protein C (activated)	BZ	-0.98743	0.147652	c
Protein C (activated)	CZ	0.558435	0.029317	a
Protein C (activated)	DZ	0.079358	0.257277	ab
Protein C (activated)	EZ	0.918183	0.158736	a
Protein disulfide-isomerase	AZ	0.857955	0.103532	a
Protein disulfide-isomerase	BZ	0.960947	0.031965	a
Protein disulfide-isomerase	CZ	-0.23576	0.137336	b
Protein disulfide-isomerase	DZ	-0.87397	0.132381	b
Protein disulfide-isomerase	EZ	-0.70918	0.206837	b
Protein-S-isoprenylcysteine O-methyltransferase	AZ	0.644899	0.052546	a
Protein-S-isoprenylcysteine O-methyltransferase	BZ	0.633771	0.041138	a
Protein-S-isoprenylcysteine O-methyltransferase	CZ	-0.64322	0.163028	b
Protein-S-isoprenylcysteine O-methyltransferase	DZ	0.140722	0.28418	ab
Protein-S-isoprenylcysteine O-methyltransferase	EZ	-0.77618	0.188502	b
Protein-serine/threonine phosphatase	AZ	0.88563	0.09317	a
Protein-serine/threonine phosphatase	BZ	0.975011	0.029088	a
Protein-serine/threonine phosphatase	CZ	-0.40405	0.165638	b
Protein-serine/threonine phosphatase	DZ	-0.76491	0.115027	b
Protein-serine/threonine phosphatase	EZ	-0.69168	0.204251	b
Protein-tyrosine-phosphatase	AZ	0.870969	0.101361	a
Protein-tyrosine-phosphatase	BZ	0.960184	0.034275	a
Protein-tyrosine-phosphatase	CZ	-0.4367	0.16771	b
Protein-tyrosine-phosphatase	DZ	-0.65148	0.111226	b
Protein-tyrosine-phosphatase	EZ	-0.74297	0.213704	b
Protochlorophyllide reductase	AZ	-0.86411	0.157224	b
Protochlorophyllide reductase	BZ	-0.98887	0.108688	b
Protochlorophyllide reductase	CZ	0.564278	0.082263	a
Protochlorophyllide reductase	DZ	0.78982	0.094512	a
Protochlorophyllide reductase	EZ	0.498889	0.204899	a
Proton-exporting ATPase	AZ	0.699411	0.128366	a
Proton-exporting ATPase	BZ	0.892786	0.034336	a
Proton-exporting ATPase	CZ	0.003824	0.135273	ab
Proton-exporting ATPase	DZ	-0.92519	0.156133	b
Proton-exporting ATPase	EZ	-0.67083	0.223729	b
Purine nucleosidase	AZ	-0.73978	0.099277	b
Purine nucleosidase	BZ	-0.87422	0.033381	b
Purine nucleosidase	CZ	0.164817	0.09851	ab
Purine nucleosidase	DZ	0.698308	0.217699	a
Purine nucleosidase	EZ	0.750876	0.228965	a
Purine-nucleoside phosphorylase	AZ	0.776691	0.075686	a
Purine-nucleoside phosphorylase	BZ	0.859228	0.023168	a
Purine-nucleoside phosphorylase	CZ	-0.31806	0.16464	b

Purine-nucleoside phosphorylase	DZ	-0.86841	0.236057	b
Purine-nucleoside phosphorylase	EZ	-0.44944	0.175967	b
Pyridoxal 5'-phosphate synthase	AZ	-0.93183	0.15888	b
Pyridoxal 5'-phosphate synthase	BZ	-0.89697	0.100521	b
Pyridoxal 5'-phosphate synthase	CZ	0.575055	0.11047	a
Pyridoxal 5'-phosphate synthase	DZ	0.795711	0.051587	a
Pyridoxal 5'-phosphate synthase	EZ	0.458035	0.217859	a
Pyridoxal kinase	AZ	0.850922	0.101775	a
Pyridoxal kinase	BZ	0.979431	0.033966	a
Pyridoxal kinase	CZ	-0.40893	0.173562	b
Pyridoxal kinase	DZ	-0.74565	0.116958	b
Pyridoxal kinase	EZ	-0.67578	0.203772	b
Pyridoxine 4-dehydrogenase	AZ	-0.88195	0.133885	b
Pyridoxine 4-dehydrogenase	BZ	-1.00674	0.046718	b
Pyridoxine 4-dehydrogenase	CZ	0.450612	0.132336	a
Pyridoxine 4-dehydrogenase	DZ	0.891107	0.086287	a
Pyridoxine 4-dehydrogenase	EZ	0.546968	0.201374	a
Pyroglutamyl-peptidase I	AZ	0.82042	0.1135	a
Pyroglutamyl-peptidase I	BZ	0.922587	0.03714	a
Pyroglutamyl-peptidase I	CZ	-0.39832	0.159725	b
Pyroglutamyl-peptidase I	DZ	-0.85429	0.115261	b
Pyroglutamyl-peptidase I	EZ	-0.49041	0.232982	b
Pyrroline-5-carboxylate reductase	AZ	-0.77961	0.125426	b
Pyrroline-5-carboxylate reductase	BZ	-0.92557	0.040358	b
Pyrroline-5-carboxylate reductase	CZ	0.774984	0.20042	a
Pyrroline-5-carboxylate reductase	DZ	0.319155	0.125875	a
Pyrroline-5-carboxylate reductase	EZ	0.611045	0.199025	a
Pyruvate decarboxylase	AZ	-0.91835	0.149848	b
Pyruvate decarboxylase	BZ	-0.91649	0.105571	b
Pyruvate decarboxylase	CZ	0.577368	0.103699	a
Pyruvate decarboxylase	DZ	0.765391	0.062963	a
Pyruvate decarboxylase	EZ	0.492081	0.222374	a
Pyruvate dehydrogenase (acetyl-transferring)	AZ	0.886897	0.100128	a
Pyruvate dehydrogenase (acetyl-transferring)	BZ	0.965368	0.033319	a
Pyruvate dehydrogenase (acetyl-transferring)	CZ	-0.44174	0.180988	b
Pyruvate dehydrogenase (acetyl-transferring)	DZ	-0.73632	0.090942	b
Pyruvate dehydrogenase (acetyl-transferring)	EZ	-0.67421	0.205713	b
Pyruvate kinase	AZ	0.866802	0.103111	a
Pyruvate kinase	BZ	0.962972	0.033353	a
Pyruvate kinase	CZ	-0.38555	0.173323	b
Pyruvate kinase	DZ	-0.76463	0.109365	b
Pyruvate kinase	EZ	-0.67959	0.206309	b
Quercetin 3-O-methyltransferase	AZ	-0.70801	0.134451	bc
Quercetin 3-O-methyltransferase	BZ	-0.92827	0.14134	c

Quercetin 3-O-methyltransferase	CZ	0.250258	0.186484	ab
Quercetin 3-O-methyltransferase	DZ	0.693414	0.068034	a
Quercetin 3-O-methyltransferase	EZ	0.69261	0.207929	a
Quinol--cytochrome-c reductase	AZ	0.840608	0.084551	a
Quinol--cytochrome-c reductase	BZ	0.923206	0.026059	a
Quinol--cytochrome-c reductase	CZ	-0.46819	0.202037	b
Quinol--cytochrome-c reductase	DZ	-0.63904	0.141662	b
Quinol--cytochrome-c reductase	EZ	-0.65658	0.201009	b
Ribitol 2-dehydrogenase	AZ	-0.88722	0.164658	b
Ribitol 2-dehydrogenase	BZ	-0.87907	0.098982	b
Ribitol 2-dehydrogenase	CZ	0.503876	0.108686	a
Ribitol 2-dehydrogenase	DZ	0.748873	0.088523	a
Ribitol 2-dehydrogenase	EZ	0.513534	0.226991	a
Riboflavin kinase	AZ	0.805196	0.095328	a
Riboflavin kinase	BZ	0.874263	0.04345	a
Riboflavin kinase	CZ	-0.56419	0.17715	b
Riboflavin kinase	DZ	-0.44359	0.190023	b
Riboflavin kinase	EZ	-0.67168	0.206369	b
Riboflavin synthase	AZ	0.919729	0.086766	a
Riboflavin synthase	BZ	0.943444	0.03795	a
Riboflavin synthase	CZ	-0.4916	0.19077	b
Riboflavin synthase	DZ	-0.76203	0.088147	b
Riboflavin synthase	EZ	-0.60954	0.200933	b
Ribonuclease III	AZ	0.80641	0.120024	a
Ribonuclease III	BZ	0.906396	0.041106	a
Ribonuclease III	CZ	-0.20233	0.168206	b
Ribonuclease III	DZ	-0.79852	0.13794	b
Ribonuclease III	EZ	-0.71196	0.20909	b
Ribonuclease P	AZ	0.882085	0.091384	a
Ribonuclease P	BZ	0.966129	0.028572	a
Ribonuclease P	CZ	-0.37796	0.170366	b
Ribonuclease P	DZ	-0.77375	0.11101	b
Ribonuclease P	EZ	-0.69651	0.206343	b
Ribonuclease T(1)	AZ	-0.8757	0.099626	b
Ribonuclease T(1)	BZ	-1.09717	0.084218	b
Ribonuclease T(1)	CZ	0.693528	0.120397	a
Ribonuclease T(1)	DZ	0.736839	0.08846	a
Ribonuclease T(1)	EZ	0.542509	0.19031	a
Ribonuclease T(2)	AZ	-0.74063	0.137514	b
Ribonuclease T(2)	BZ	-0.86223	0.046116	b
Ribonuclease T(2)	CZ	0.139229	0.116909	ab
Ribonuclease T(2)	DZ	0.821042	0.191821	a
Ribonuclease T(2)	EZ	0.642584	0.220412	a
Ribonucleoside-diphosphate reductase	AZ	0.859871	0.092574	a

Ribonucleoside-diphosphate reductase	BZ	0.956139	0.027798	a
Ribonucleoside-diphosphate reductase	CZ	-0.3089	0.162232	b
Ribonucleoside-diphosphate reductase	DZ	-0.77212	0.130171	b
Ribonucleoside-diphosphate reductase	EZ	-0.73499	0.207665	b
Ribose-5-phosphate isomerase	AZ	-0.87156	0.098197	b
Ribose-5-phosphate isomerase	BZ	-0.96197	0.045695	b
Ribose-5-phosphate isomerase	CZ	0.359941	0.112812	a
Ribose-5-phosphate isomerase	DZ	0.877458	0.173355	a
Ribose-5-phosphate isomerase	EZ	0.596138	0.196205	a
Ribose-phosphate diphosphokinase	AZ	0.857344	0.10419	a
Ribose-phosphate diphosphokinase	BZ	0.96563	0.030646	a
Ribose-phosphate diphosphokinase	CZ	-0.60631	0.199343	b
Ribose-phosphate diphosphokinase	DZ	-0.6029	0.100503	b
Ribose-phosphate diphosphokinase	EZ	-0.61377	0.199004	b
Ribosomal-protein-alanine N-acetyltransferase	AZ	-0.9126	0.166002	b
Ribosomal-protein-alanine N-acetyltransferase	BZ	-0.53757	0.147183	ab
Ribosomal-protein-alanine N-acetyltransferase	CZ	0.661406	0.206041	a
Ribosomal-protein-alanine N-acetyltransferase	DZ	0.612076	0.105216	a
Ribosomal-protein-alanine N-acetyltransferase	EZ	0.176687	0.197487	ab
Ribulose-phosphate 3-epimerase	AZ	0.866802	0.103111	a
Ribulose-phosphate 3-epimerase	BZ	0.962972	0.033353	a
Ribulose-phosphate 3-epimerase	CZ	-0.38555	0.173323	b
Ribulose-phosphate 3-epimerase	DZ	-0.76463	0.109365	b
Ribulose-phosphate 3-epimerase	EZ	-0.67959	0.206309	b
RNA 3'-terminal-phosphate cyclase (ATP)	AZ	-0.83512	0.127726	b
RNA 3'-terminal-phosphate cyclase (ATP)	BZ	-0.94771	0.042571	b
RNA 3'-terminal-phosphate cyclase (ATP)	CZ	0.300702	0.112808	a
RNA 3'-terminal-phosphate cyclase (ATP)	DZ	0.909098	0.169	a
RNA 3'-terminal-phosphate cyclase (ATP)	EZ	0.573033	0.196331	a
RNA ligase (ATP)	AZ	0.866802	0.103111	a
RNA ligase (ATP)	BZ	0.962972	0.033353	a
RNA ligase (ATP)	CZ	-0.38555	0.173323	b
RNA ligase (ATP)	DZ	-0.76463	0.109365	b
RNA ligase (ATP)	EZ	-0.67959	0.206309	b
RNA uridylyltransferase	AZ	-0.90959	0.121271	b
RNA uridylyltransferase	BZ	-0.91939	0.166399	b
RNA uridylyltransferase	CZ	0.669095	0.113269	a
RNA uridylyltransferase	DZ	0.584316	0.092462	a
RNA uridylyltransferase	EZ	0.575561	0.191247	a
RNA-directed DNA polymerase	AZ	-1.02735	0.188942	b
RNA-directed DNA polymerase	BZ	-0.81797	0.114007	b
RNA-directed DNA polymerase	CZ	0.707881	0.103596	a
RNA-directed DNA polymerase	DZ	0.749693	0.098763	a
RNA-directed DNA polymerase	EZ	0.387743	0.154652	a

S-formylglutathione hydrolase	AZ	-0.95534	0.149077	b
S-formylglutathione hydrolase	BZ	-0.92431	0.123801	b
S-formylglutathione hydrolase	CZ	0.657878	0.139123	a
S-formylglutathione hydrolase	DZ	0.758671	0.057479	a
S-formylglutathione hydrolase	EZ	0.463103	0.174058	a
S-methyl-5-thioribose-1-phosphate isomerase	AZ	-0.88886	0.086899	b
S-methyl-5-thioribose-1-phosphate isomerase	BZ	-1.11655	0.075601	b
S-methyl-5-thioribose-1-phosphate isomerase	CZ	0.697441	0.112973	a
S-methyl-5-thioribose-1-phosphate isomerase	DZ	0.743929	0.089425	a
S-methyl-5-thioribose-1-phosphate isomerase	EZ	0.564043	0.190355	a
S-methyl-5'-thioadenosine phosphorylase	AZ	0.918304	0.097832	a
S-methyl-5'-thioadenosine phosphorylase	BZ	0.897011	0.063389	a
S-methyl-5'-thioadenosine phosphorylase	CZ	-0.42035	0.203763	b
S-methyl-5'-thioadenosine phosphorylase	DZ	-0.85549	0.069863	b
S-methyl-5'-thioadenosine phosphorylase	EZ	-0.53947	0.193999	b
Saccharolysin	AZ	-0.88637	0.166599	b
Saccharolysin	BZ	-0.91336	0.09465	b
Saccharolysin	CZ	0.495308	0.113807	a
Saccharolysin	DZ	0.82173	0.05449	a
Saccharolysin	EZ	0.482695	0.220663	a
Saccharopine dehydrogenase (NAD(+), L-lysine-forming)	AZ	0.905016	0.098152	a
Saccharopine dehydrogenase (NAD(+), L-lysine-forming)	BZ	0.902619	0.062589	a
Saccharopine dehydrogenase (NAD(+), L-lysine-forming)	CZ	-0.3993	0.202093	b
Saccharopine dehydrogenase (NAD(+), L-lysine-forming)	DZ	-0.89303	0.078605	b
Saccharopine dehydrogenase (NAD(+), L-lysine-forming)	EZ	-0.5153	0.191353	b
Saccharopine dehydrogenase (NADP(+), L-glutamate-forming)	AZ	0.808766	0.082495	a
Saccharopine dehydrogenase (NADP(+), L-glutamate-forming)	BZ	0.883095	0.020419	a
Saccharopine dehydrogenase (NADP(+), L-glutamate-forming)	CZ	-0.65106	0.281665	b
Saccharopine dehydrogenase (NADP(+), L-glutamate-forming)	DZ	-0.61126	0.079686	b
Saccharopine dehydrogenase (NADP(+), L-glutamate-forming)	EZ	-0.42955	0.162153	b
Salicylate 1-monooxygenase	AZ	-0.91529	0.162077	b
Salicylate 1-monooxygenase	BZ	-0.89583	0.098679	b
Salicylate 1-monooxygenase	CZ	0.536406	0.103148	a
Salicylate 1-monooxygenase	DZ	0.812896	0.052452	a
Salicylate 1-monooxygenase	EZ	0.461811	0.224722	a
Sarcosine oxidase	AZ	-0.9228	0.168044	b
Sarcosine oxidase	BZ	-0.82627	0.102435	b
Sarcosine oxidase	CZ	0.602877	0.13948	a
Sarcosine oxidase	DZ	0.708048	0.044679	a
Sarcosine oxidase	EZ	0.438138	0.224201	a
Sedoheptulose-bisphosphatase	AZ	-0.84027	0.169001	b
Sedoheptulose-bisphosphatase	BZ	-0.98188	0.08269	b
Sedoheptulose-bisphosphatase	CZ	0.640626	0.104378	a
Sedoheptulose-bisphosphatase	DZ	0.850274	0.104951	a

Sedoheptulose-bisphosphatase	EZ	0.331254	0.192429	a
Selenocysteine lyase	AZ	-0.78851	0.208289	c
Selenocysteine lyase	BZ	-0.7261	0.136522	bc
Selenocysteine lyase	CZ	0.420987	0.162662	ab
Selenocysteine lyase	DZ	0.716772	0.0706	a
Selenocysteine lyase	EZ	0.376843	0.217798	ab
Sepiapterin reductase (L-erythro-7,8-dihydrobiopterin forming)	AZ	-0.58234	0.053899	bc
Sepiapterin reductase (L-erythro-7,8-dihydrobiopterin forming)	BZ	-0.99609	0.145845	c
Sepiapterin reductase (L-erythro-7,8-dihydrobiopterin forming)	CZ	0.626285	0.015944	a
Sepiapterin reductase (L-erythro-7,8-dihydrobiopterin forming)	DZ	0.063598	0.255091	ab
Sepiapterin reductase (L-erythro-7,8-dihydrobiopterin forming)	EZ	0.888551	0.156979	a
Serine C-palmitoyltransferase	AZ	0.822052	0.104426	a
Serine C-palmitoyltransferase	BZ	0.979212	0.023924	a
Serine C-palmitoyltransferase	CZ	-0.38339	0.17008	b
Serine C-palmitoyltransferase	DZ	-0.67795	0.127074	b
Serine C-palmitoyltransferase	EZ	-0.73992	0.20979	b
Serine O-acetyltransferase	AZ	-0.87917	0.086704	b
Serine O-acetyltransferase	BZ	-1.15484	0.05978	b
Serine O-acetyltransferase	CZ	0.722806	0.106329	a
Serine O-acetyltransferase	DZ	0.644496	0.091649	a
Serine O-acetyltransferase	EZ	0.666712	0.186432	a
Serine--tRNA ligase	AZ	0.900887	0.102398	a
Serine--tRNA ligase	BZ	0.933044	0.048854	a
Serine--tRNA ligase	CZ	-0.41182	0.188129	b
Serine--tRNA ligase	DZ	-0.80109	0.081168	b
Serine--tRNA ligase	EZ	-0.62102	0.202949	b
Shikimate O-hydroxycinnamoyltransferase	AZ	-0.91512	0.126729	b
Shikimate O-hydroxycinnamoyltransferase	BZ	-0.89837	0.177292	b
Shikimate O-hydroxycinnamoyltransferase	CZ	0.649433	0.103929	a
Shikimate O-hydroxycinnamoyltransferase	DZ	0.55261	0.083907	a
Shikimate O-hydroxycinnamoyltransferase	EZ	0.611446	0.193364	a
Signal peptidase I	AZ	0.884217	0.079288	a
Signal peptidase I	BZ	0.962615	0.023938	a
Signal peptidase I	CZ	-0.38384	0.16995	b
Signal peptidase I	DZ	-0.75873	0.132019	b
Signal peptidase I	EZ	-0.70426	0.201135	b
Site-specific DNA-methyltransferase (adenine-specific)	AZ	0.856111	0.08184	a
Site-specific DNA-methyltransferase (adenine-specific)	BZ	0.919426	0.029091	a
Site-specific DNA-methyltransferase (adenine-specific)	CZ	-0.46103	0.207462	b
Site-specific DNA-methyltransferase (adenine-specific)	DZ	-0.68163	0.125689	b
Site-specific DNA-methyltransferase (adenine-specific)	EZ	-0.63288	0.202038	b
Sodium/potassium-exchanging ATPase	AZ	0.835335	0.056305	a
Sodium/potassium-exchanging ATPase	BZ	0.902473	0.014843	a
Sodium/potassium-exchanging ATPase	CZ	-0.10518	0.142421	b

Sodium/potassium-exchanging ATPase	DZ	-0.80794	0.174168	b
Sodium/potassium-exchanging ATPase	EZ	-0.82469	0.207804	b
Spermidine synthase	AZ	0.866802	0.103111	a
Spermidine synthase	BZ	0.962972	0.033353	a
Spermidine synthase	CZ	-0.38555	0.173323	b
Spermidine synthase	DZ	-0.76463	0.109365	b
Spermidine synthase	EZ	-0.67959	0.206309	b
Sphinganine kinase	AZ	0.816988	0.113477	a
Sphinganine kinase	BZ	0.921547	0.037116	a
Sphinganine kinase	CZ	-0.17017	0.1783	b
Sphinganine kinase	DZ	-0.91222	0.113475	b
Sphinganine kinase	EZ	-0.65614	0.201644	b
Sphinganine-1-phosphate aldolase	AZ	0.866729	0.104702	a
Sphinganine-1-phosphate aldolase	BZ	0.964329	0.033866	a
Sphinganine-1-phosphate aldolase	CZ	-0.40344	0.175676	b
Sphinganine-1-phosphate aldolase	DZ	-0.77754	0.108884	b
Sphinganine-1-phosphate aldolase	EZ	-0.65008	0.204006	b
Sphingomyelin phosphodiesterase	AZ	0.784268	0.098971	a
Sphingomyelin phosphodiesterase	BZ	0.903019	0.036847	a
Sphingomyelin phosphodiesterase	CZ	-0.32121	0.175714	b
Sphingomyelin phosphodiesterase	DZ	-0.52108	0.120714	b
Sphingomyelin phosphodiesterase	EZ	-0.84499	0.239662	b
Sphingosine N-acyltransferase	AZ	0.871711	0.118024	a
Sphingosine N-acyltransferase	BZ	0.943056	0.04701	a
Sphingosine N-acyltransferase	CZ	-0.20518	0.132939	b
Sphingosine N-acyltransferase	DZ	-0.9022	0.088279	b
Sphingosine N-acyltransferase	EZ	-0.70739	0.219339	b
Squalene synthase	AZ	0.882667	0.101383	a
Squalene synthase	BZ	0.954955	0.033277	a
Squalene synthase	CZ	-0.39173	0.172807	b
Squalene synthase	DZ	-0.75978	0.107282	b
Squalene synthase	EZ	-0.68611	0.206439	b
Sterol 24-C-methyltransferase	AZ	-0.92044	0.100147	b
Sterol 24-C-methyltransferase	BZ	-0.96568	0.043471	b
Sterol 24-C-methyltransferase	CZ	0.420465	0.147305	a
Sterol 24-C-methyltransferase	DZ	0.959789	0.102555	a
Sterol 24-C-methyltransferase	EZ	0.505864	0.197947	a
Sterol 3-beta-glucosyltransferase	AZ	-0.9213	0.106494	b
Sterol 3-beta-glucosyltransferase	BZ	-0.95023	0.042517	b
Sterol 3-beta-glucosyltransferase	CZ	0.515673	0.160163	a
Sterol 3-beta-glucosyltransferase	DZ	0.623076	0.085367	a
Sterol 3-beta-glucosyltransferase	EZ	0.732782	0.216263	a
Sterol esterase	AZ	-0.95619	0.126852	b
Sterol esterase	BZ	-0.89137	0.160598	b

Sterol esterase	CZ	0.658516	0.120518	a
Sterol esterase	DZ	0.682299	0.066149	a
Sterol esterase	EZ	0.506755	0.18967	a
Sterol O-acyltransferase	AZ	-0.62241	0.116318	b
Sterol O-acyltransferase	BZ	-0.86753	0.050499	b
Sterol O-acyltransferase	CZ	0.167094	0.139309	ab
Sterol O-acyltransferase	DZ	0.547991	0.216053	a
Sterol O-acyltransferase	EZ	0.774859	0.233244	a
Succinate dehydrogenase (quinone)	AZ	0.866777	0.088934	a
Succinate dehydrogenase (quinone)	BZ	0.97725	0.025184	a
Succinate dehydrogenase (quinone)	CZ	-0.57324	0.181209	b
Succinate dehydrogenase (quinone)	DZ	-0.63531	0.128612	b
Succinate dehydrogenase (quinone)	EZ	-0.63548	0.1999	b
Succinate--CoA ligase (ADP-forming)	AZ	0.815463	0.054468	a
Succinate--CoA ligase (ADP-forming)	BZ	0.875371	0.015079	a
Succinate--CoA ligase (ADP-forming)	CZ	-0.03028	0.133324	ab
Succinate--CoA ligase (ADP-forming)	DZ	-0.86765	0.197284	b
Succinate--CoA ligase (ADP-forming)	EZ	-0.7929	0.198559	b
Succinate--CoA ligase (GDP-forming)	AZ	-0.84226	0.124374	b
Succinate--CoA ligase (GDP-forming)	BZ	-0.88413	0.057063	b
Succinate--CoA ligase (GDP-forming)	CZ	0.450103	0.18477	a
Succinate--CoA ligase (GDP-forming)	DZ	0.826343	0.155507	a
Succinate--CoA ligase (GDP-forming)	EZ	0.449945	0.184401	a
Succinate--hydroxymethylglutarate CoA-transferase	AZ	-0.89823	0.109463	b
Succinate--hydroxymethylglutarate CoA-transferase	BZ	-0.97781	0.146137	b
Succinate--hydroxymethylglutarate CoA-transferase	CZ	0.658889	0.126298	a
Succinate--hydroxymethylglutarate CoA-transferase	DZ	0.678782	0.085856	a
Succinate--hydroxymethylglutarate CoA-transferase	EZ	0.538361	0.189626	a
Succinate-semialdehyde dehydrogenase (NAD(+))	AZ	-0.8909	0.11335	b
Succinate-semialdehyde dehydrogenase (NAD(+))	BZ	-1.10688	0.05224	b
Succinate-semialdehyde dehydrogenase (NAD(+))	CZ	0.774978	0.094584	a
Succinate-semialdehyde dehydrogenase (NAD(+))	DZ	0.732305	0.082829	a
Succinate-semialdehyde dehydrogenase (NAD(+))	EZ	0.490496	0.196975	a
Succinate-semialdehyde dehydrogenase (NAD(P)(+))	AZ	-0.75558	0.131723	b
Succinate-semialdehyde dehydrogenase (NAD(P)(+))	BZ	-0.87632	0.057549	b
Succinate-semialdehyde dehydrogenase (NAD(P)(+))	CZ	0.473388	0.220339	a
Succinate-semialdehyde dehydrogenase (NAD(P)(+))	DZ	0.719918	0.157129	a
Succinate-semialdehyde dehydrogenase (NAD(P)(+))	EZ	0.438591	0.180054	a
Sucrose-phosphate synthase	AZ	-1.07777	0.166962	c
Sucrose-phosphate synthase	BZ	-0.48483	0.115813	bc
Sucrose-phosphate synthase	CZ	0.2197	0.151521	ab
Sucrose-phosphate synthase	DZ	1.036309	0.048744	a
Sucrose-phosphate synthase	EZ	0.306586	0.206724	ab
Sulfate adenylyltransferase	AZ	0.866802	0.103111	a

Sulfate adenylyltransferase	BZ	0.962972	0.033353	a
Sulfate adenylyltransferase	CZ	-0.38555	0.173323	b
Sulfate adenylyltransferase	DZ	-0.76463	0.109365	b
Sulfate adenylyltransferase	EZ	-0.67959	0.206309	b
Superoxide dismutase	AZ	0.898702	0.080973	a
Superoxide dismutase	BZ	0.922802	0.043786	a
Superoxide dismutase	CZ	-0.69144	0.224337	b
Superoxide dismutase	DZ	-0.62913	0.051937	b
Superoxide dismutase	EZ	-0.50094	0.198239	b
Tartrate dehydrogenase	AZ	-0.88856	0.183777	b
Tartrate dehydrogenase	BZ	-0.77857	0.112058	b
Tartrate dehydrogenase	CZ	0.533477	0.115542	a
Tartrate dehydrogenase	DZ	0.648273	0.06617	a
Tartrate dehydrogenase	EZ	0.485386	0.24317	a
Taurine dioxygenase	AZ	-0.85107	0.089108	b
Taurine dioxygenase	BZ	-1.17249	0.053158	b
Taurine dioxygenase	CZ	0.744426	0.114534	a
Taurine dioxygenase	DZ	0.664467	0.10014	a
Taurine dioxygenase	EZ	0.614673	0.180164	a
Tetrahydroxynaphthalene reductase	AZ	-0.9181	0.130935	b
Tetrahydroxynaphthalene reductase	BZ	-0.88241	0.183957	b
Tetrahydroxynaphthalene reductase	CZ	0.634034	0.096733	a
Tetrahydroxynaphthalene reductase	DZ	0.52718	0.077986	a
Tetrahydroxynaphthalene reductase	EZ	0.639301	0.195108	a
Thiamine diphosphokinase	AZ	0.864973	0.024133	a
Thiamine diphosphokinase	BZ	0.963909	0.060995	a
Thiamine diphosphokinase	CZ	-0.45631	0.116133	b
Thiamine diphosphokinase	DZ	-0.81821	0.19672	b
Thiamine diphosphokinase	EZ	-0.55437	0.200988	b
Thimet oligopeptidase	AZ	0.835083	0.040204	a
Thimet oligopeptidase	BZ	0.883621	0.010458	a
Thimet oligopeptidase	CZ	-0.14654	0.161629	b
Thimet oligopeptidase	DZ	-0.73186	0.183458	b
Thimet oligopeptidase	EZ	-0.8403	0.203534	b
Thiol oxidase	AZ	-0.91701	0.122578	b
Thiol oxidase	BZ	-0.94592	0.148203	b
Thiol oxidase	CZ	0.623185	0.1217	a
Thiol oxidase	DZ	0.74728	0.079514	a
Thiol oxidase	EZ	0.492468	0.188753	a
Thiopurine S-methyltransferase	AZ	-0.8771	0.134277	b
Thiopurine S-methyltransferase	BZ	-0.83881	0.178408	b
Thiopurine S-methyltransferase	CZ	0.673142	0.135806	a
Thiopurine S-methyltransferase	DZ	0.607619	0.112769	a
Thiopurine S-methyltransferase	EZ	0.435147	0.186416	a

Thiosulfate sulfurtransferase	AZ	0.838686	0.086023	a
Thiosulfate sulfurtransferase	BZ	0.903762	0.027048	a
Thiosulfate sulfurtransferase	CZ	-0.64255	0.237478	b
Thiosulfate sulfurtransferase	DZ	-0.54647	0.103064	b
Thiosulfate sulfurtransferase	EZ	-0.55343	0.194814	b
Threonine--tRNA ligase	AZ	0.785599	0.140446	a
Threonine--tRNA ligase	BZ	0.970555	0.038031	a
Threonine--tRNA ligase	CZ	-0.44283	0.195748	b
Threonine--tRNA ligase	DZ	-0.71725	0.099068	b
Threonine--tRNA ligase	EZ	-0.59608	0.198613	b
Thromboxane-A synthase	AZ	-0.77538	0.15744	b
Thromboxane-A synthase	BZ	-0.9576	0.118609	b
Thromboxane-A synthase	CZ	0.487072	0.090306	a
Thromboxane-A synthase	DZ	0.736971	0.095688	a
Thromboxane-A synthase	EZ	0.508941	0.236544	a
Thymidylate synthase	AZ	0.893788	0.084005	a
Thymidylate synthase	BZ	0.963543	0.027262	a
Thymidylate synthase	CZ	-0.37462	0.1691	b
Thymidylate synthase	DZ	-0.77873	0.117569	b
Thymidylate synthase	EZ	-0.70398	0.203228	b
Tocopherol O-methyltransferase	AZ	-0.84934	0.174568	b
Tocopherol O-methyltransferase	BZ	-0.97066	0.077019	b
Tocopherol O-methyltransferase	CZ	0.592773	0.100238	a
Tocopherol O-methyltransferase	DZ	0.875237	0.106271	a
Tocopherol O-methyltransferase	EZ	0.35199	0.192377	a
Trans-1,2-dihydrobenzene-1,2-diol dehydrogenase	AZ	-0.93262	0.157576	b
Trans-1,2-dihydrobenzene-1,2-diol dehydrogenase	BZ	-0.86792	0.09442	b
Trans-1,2-dihydrobenzene-1,2-diol dehydrogenase	CZ	0.545586	0.129898	a
Trans-1,2-dihydrobenzene-1,2-diol dehydrogenase	DZ	0.804502	0.04658	a
Trans-1,2-dihydrobenzene-1,2-diol dehydrogenase	EZ	0.450448	0.220611	a
Trans-2-enoyl-CoA reductase (NADPH)	AZ	-0.79617	0.129543	b
Trans-2-enoyl-CoA reductase (NADPH)	BZ	-0.91291	0.050173	b
Trans-2-enoyl-CoA reductase (NADPH)	CZ	0.651287	0.148972	a
Trans-2-enoyl-CoA reductase (NADPH)	DZ	0.565479	0.186015	a
Trans-2-enoyl-CoA reductase (NADPH)	EZ	0.492315	0.203044	a
Trans-L-3-hydroxyproline dehydratase	AZ	-0.87495	0.09083	b
Trans-L-3-hydroxyproline dehydratase	BZ	-1.14857	0.057719	b
Trans-L-3-hydroxyproline dehydratase	CZ	0.750665	0.108658	a
Trans-L-3-hydroxyproline dehydratase	DZ	0.651409	0.096984	a
Trans-L-3-hydroxyproline dehydratase	EZ	0.621452	0.185112	a
Transaldolase	AZ	-0.91787	0.06319	b
Transaldolase	BZ	-0.91369	0.032196	b
Transaldolase	CZ	0.69238	0.196832	a
Transaldolase	DZ	0.589256	0.147162	a

Transaldolase	EZ	0.549932	0.185075	a
Transketolase	AZ	-0.76619	0.134022	b
Transketolase	BZ	-0.78831	0.079612	b
Transketolase	CZ	0.336177	0.191218	a
Transketolase	DZ	0.425695	0.156394	a
Transketolase	EZ	0.792623	0.219382	a
Triacylglycerol lipase	AZ	0.739139	0.135567	a
Triacylglycerol lipase	BZ	0.828585	0.050051	a
Triacylglycerol lipase	CZ	-0.14458	0.212314	ab
Triacylglycerol lipase	DZ	-0.87245	0.135059	b
Triacylglycerol lipase	EZ	-0.55069	0.19972	b
Trimethyllysine dioxygenase	AZ	0.724547	0.097116	ab
Trimethyllysine dioxygenase	BZ	0.935188	0.043041	a
Trimethyllysine dioxygenase	CZ	-0.19898	0.147599	bc
Trimethyllysine dioxygenase	DZ	-0.80715	0.21646	c
Trimethyllysine dioxygenase	EZ	-0.6536	0.18489	c
Triose-phosphate isomerase	AZ	-0.72986	0.104502	bc
Triose-phosphate isomerase	BZ	-0.95087	0.057028	c
Triose-phosphate isomerase	CZ	0.119072	0.06577	ab
Triose-phosphate isomerase	DZ	0.848019	0.218178	a
Triose-phosphate isomerase	EZ	0.713643	0.199817	a
Tripeptidyl-peptidase I	AZ	-0.90782	0.167084	b
Tripeptidyl-peptidase I	BZ	-0.90856	0.097864	b
Tripeptidyl-peptidase I	CZ	0.492704	0.095626	a
Tripeptidyl-peptidase I	DZ	0.840539	0.04869	a
Tripeptidyl-peptidase I	EZ	0.483142	0.222204	a
Tripeptidyl-peptidase II	AZ	0.83489	0.044881	a
Tripeptidyl-peptidase II	BZ	0.88892	0.011741	a
Tripeptidyl-peptidase II	CZ	-0.23084	0.174805	b
Tripeptidyl-peptidase II	DZ	-0.75841	0.192929	b
Tripeptidyl-peptidase II	EZ	-0.73456	0.193147	b
tRNA (5-methylaminomethyl-2-thiouridylate)-methyltransferase	AZ	0.826436	0.068731	a
tRNA (5-methylaminomethyl-2-thiouridylate)-methyltransferase	BZ	0.908883	0.018242	a
tRNA (5-methylaminomethyl-2-thiouridylate)-methyltransferase	CZ	-0.19872	0.161896	b
tRNA (5-methylaminomethyl-2-thiouridylate)-methyltransferase	DZ	-0.84935	0.180507	b
tRNA (5-methylaminomethyl-2-thiouridylate)-methyltransferase	EZ	-0.68725	0.197578	b
tRNA (guanine(46)-N(7))-methyltransferase	AZ	0.862186	0.100593	a
tRNA (guanine(46)-N(7))-methyltransferase	BZ	0.959563	0.032281	a
tRNA (guanine(46)-N(7))-methyltransferase	CZ	-0.36439	0.170408	b
tRNA (guanine(46)-N(7))-methyltransferase	DZ	-0.74282	0.108914	b
tRNA (guanine(46)-N(7))-methyltransferase	EZ	-0.71454	0.212074	b
tRNA (guanosine(18)-2'-O)-methyltransferase	AZ	-0.7774	0.187167	b
tRNA (guanosine(18)-2'-O)-methyltransferase	BZ	-0.84381	0.144698	b
tRNA (guanosine(18)-2'-O)-methyltransferase	CZ	0.495082	0.087418	a

tRNA (guanosine(18)-2'-O)-methyltransferase	DZ	0.516678	0.101196	a
tRNA (guanosine(18)-2'-O)-methyltransferase	EZ	0.609448	0.236972	a
tRNA (uracil(54)-C(5))-methyltransferase	AZ	-0.96437	0.167865	b
tRNA (uracil(54)-C(5))-methyltransferase	BZ	-0.89515	0.134388	b
tRNA (uracil(54)-C(5))-methyltransferase	CZ	0.601912	0.089992	a
tRNA (uracil(54)-C(5))-methyltransferase	DZ	0.717486	0.102676	a
tRNA (uracil(54)-C(5))-methyltransferase	EZ	0.54012	0.173537	a
tRNA nucleotidyltransferase	AZ	-0.9114	0.177221	b
tRNA nucleotidyltransferase	BZ	-0.77529	0.119123	b
tRNA nucleotidyltransferase	CZ	0.489543	0.101087	a
tRNA nucleotidyltransferase	DZ	0.710699	0.092409	a
tRNA nucleotidyltransferase	EZ	0.486447	0.234498	a
tRNA pseudouridine(38-40) synthase	AZ	0.880377	0.085356	a
tRNA pseudouridine(38-40) synthase	BZ	0.971534	0.024657	a
tRNA pseudouridine(38-40) synthase	CZ	-0.36692	0.165544	b
tRNA pseudouridine(38-40) synthase	DZ	-0.77025	0.124574	b
tRNA pseudouridine(38-40) synthase	EZ	-0.71474	0.203217	b
tRNA-guanine(34) transglycosylase	AZ	-0.93331	0.156848	b
tRNA-guanine(34) transglycosylase	BZ	-0.91161	0.092082	b
tRNA-guanine(34) transglycosylase	CZ	0.522441	0.09764	a
tRNA-guanine(34) transglycosylase	DZ	0.865867	0.051099	a
tRNA-guanine(34) transglycosylase	EZ	0.456605	0.219346	a
Tropinone reductase I	AZ	-0.92494	0.146354	b
Tropinone reductase I	BZ	-0.90168	0.106962	b
Tropinone reductase I	CZ	0.578397	0.101142	a
Tropinone reductase I	DZ	0.751591	0.073557	a
Tropinone reductase I	EZ	0.496631	0.225319	a
Tropinone reductase II	AZ	-1.02877	0.15774	b
Tropinone reductase II	BZ	-0.22402	0.205929	ab
Tropinone reductase II	CZ	0.315487	0.184408	a
Tropinone reductase II	DZ	0.814878	0.11712	a
Tropinone reductase II	EZ	0.12243	0.175021	ab
Trypsin	AZ	-0.88395	0.16718	b
Trypsin	BZ	-0.92799	0.08825	b
Trypsin	CZ	0.571765	0.149068	a
Trypsin	DZ	0.470916	0.060853	a
Trypsin	EZ	0.769253	0.197967	a
Tryptophan--tRNA ligase	AZ	0.836398	0.118646	a
Tryptophan--tRNA ligase	BZ	0.950925	0.036297	a
Tryptophan--tRNA ligase	CZ	-0.29716	0.161371	b
Tryptophan--tRNA ligase	DZ	-0.89031	0.131278	b
Tryptophan--tRNA ligase	EZ	-0.59985	0.19774	b
Tubulin--tyrosine ligase	AZ	-0.97935	0.163142	b
Tubulin--tyrosine ligase	BZ	-0.78113	0.090227	b

Tubulin--tyrosine ligase	CZ	0.456724	0.09938	a
Tubulin--tyrosine ligase	DZ	0.938962	0.086716	a
Tubulin--tyrosine ligase	EZ	0.364791	0.219585	a
Type I site-specific deoxyribonuclease	AZ	-0.76153	0.137925	b
Type I site-specific deoxyribonuclease	BZ	-0.8598	0.169786	b
Type I site-specific deoxyribonuclease	CZ	0.584179	0.140283	a
Type I site-specific deoxyribonuclease	DZ	0.279196	0.174326	a
Type I site-specific deoxyribonuclease	EZ	0.757954	0.162591	a
Tyrosine decarboxylase	AZ	-0.87359	0.162729	b
Tyrosine decarboxylase	BZ	-0.91699	0.094296	b
Tyrosine decarboxylase	CZ	0.613292	0.103382	a
Tyrosine decarboxylase	DZ	0.759466	0.08515	a
Tyrosine decarboxylase	EZ	0.417813	0.223863	a
Tyrosine transaminase	AZ	0.74654	0.086983	ab
Tyrosine transaminase	BZ	0.890337	0.030078	a
Tyrosine transaminase	CZ	-0.19819	0.185751	bc
Tyrosine transaminase	DZ	-0.54985	0.130912	c
Tyrosine transaminase	EZ	-0.88884	0.236229	c
Tyrosine--tRNA ligase	AZ	0.775952	0.100415	a
Tyrosine--tRNA ligase	BZ	0.980717	0.015534	a
Tyrosine--tRNA ligase	CZ	-0.13891	0.113263	b
Tyrosine--tRNA ligase	DZ	-0.89315	0.158407	b
Tyrosine--tRNA ligase	EZ	-0.7246	0.215536	b
Ubiquitinyl hydrolase 1	AZ	-0.89597	0.087432	b
Ubiquitinyl hydrolase 1	BZ	-0.92198	0.0632	b
Ubiquitinyl hydrolase 1	CZ	0.558067	0.18277	a
Ubiquitinyl hydrolase 1	DZ	0.72429	0.133602	a
Ubiquitinyl hydrolase 1	EZ	0.535592	0.19576	a
UDP-glucose 6-dehydrogenase	AZ	0.847989	0.081435	a
UDP-glucose 6-dehydrogenase	BZ	0.969791	0.041106	a
UDP-glucose 6-dehydrogenase	CZ	-0.56007	0.190107	b
UDP-glucose 6-dehydrogenase	DZ	-0.64121	0.150531	b
UDP-glucose 6-dehydrogenase	EZ	-0.6165	0.186157	b
UDP-glucose--hexose-1-phosphate uridylyltransferase	AZ	-0.92378	0.163429	b
UDP-glucose--hexose-1-phosphate uridylyltransferase	BZ	-0.85881	0.108954	b
UDP-glucose--hexose-1-phosphate uridylyltransferase	CZ	0.56172	0.101788	a
UDP-glucose--hexose-1-phosphate uridylyltransferase	DZ	0.732521	0.069869	a
UDP-glucose--hexose-1-phosphate uridylyltransferase	EZ	0.488351	0.228257	a
UDP-glucuronate 4-epimerase	AZ	-0.89325	0.0923	b
UDP-glucuronate 4-epimerase	BZ	-1.1266	0.062867	b
UDP-glucuronate 4-epimerase	CZ	0.751926	0.105013	a
UDP-glucuronate 4-epimerase	DZ	0.639498	0.094012	a
UDP-glucuronate 4-epimerase	EZ	0.628425	0.189323	a
UDP-glucuronate decarboxylase	AZ	-0.93344	0.143638	b

UDP-glucuronate decarboxylase	BZ	-0.94157	0.092397	b
UDP-glucuronate decarboxylase	CZ	0.433413	0.15687	a
UDP-glucuronate decarboxylase	DZ	0.916182	0.05031	a
UDP-glucuronate decarboxylase	EZ	0.525413	0.175026	a
UDP-N-acetylglucosamine diphosphorylase	AZ	0.8557	0.081742	a
UDP-N-acetylglucosamine diphosphorylase	BZ	0.918939	0.029086	a
UDP-N-acetylglucosamine diphosphorylase	CZ	-0.46179	0.207909	b
UDP-N-acetylglucosamine diphosphorylase	DZ	-0.68075	0.125612	b
UDP-N-acetylglucosamine diphosphorylase	EZ	-0.6321	0.202048	b
UMP/CMP kinase	AZ	0.835083	0.040204	a
UMP/CMP kinase	BZ	0.883621	0.010458	a
UMP/CMP kinase	CZ	-0.14654	0.161629	b
UMP/CMP kinase	DZ	-0.73186	0.183458	b
UMP/CMP kinase	EZ	-0.8403	0.203534	b
Unspecific monooxygenase	AZ	-0.92722	0.149551	b
Unspecific monooxygenase	BZ	-0.91378	0.066139	b
Unspecific monooxygenase	CZ	0.344665	0.131433	a
Unspecific monooxygenase	DZ	0.947686	0.044262	a
Unspecific monooxygenase	EZ	0.548647	0.208313	a
Uracil phosphoribosyltransferase	AZ	-0.86695	0.099366	b
Uracil phosphoribosyltransferase	BZ	-0.98809	0.036861	b
Uracil phosphoribosyltransferase	CZ	0.377441	0.167029	a
Uracil phosphoribosyltransferase	DZ	0.901755	0.108881	a
Uracil phosphoribosyltransferase	EZ	0.57585	0.195591	a
Urease	AZ	0.811517	0.119597	a
Urease	BZ	0.839779	0.05726	a
Urease	CZ	0.188843	0.075642	a
Urease	DZ	-1.07171	0.110885	b
Urease	EZ	-0.76843	0.217889	b
Ureidoglycolate lyase	AZ	-0.90944	0.121381	b
Ureidoglycolate lyase	BZ	-0.91925	0.166549	b
Ureidoglycolate lyase	CZ	0.670664	0.113371	a
Ureidoglycolate lyase	DZ	0.584315	0.092782	a
Ureidoglycolate lyase	EZ	0.573709	0.190903	a
Uridine kinase	AZ	0.820464	0.093983	a
Uridine kinase	BZ	0.974281	0.02145	a
Uridine kinase	CZ	-0.41705	0.172497	b
Uridine kinase	DZ	-0.65111	0.145889	b
Uridine kinase	EZ	-0.72659	0.20557	b
Uridine nucleosidase	AZ	-0.91298	0.129411	b
Uridine nucleosidase	BZ	-0.92122	0.163767	b
Uridine nucleosidase	CZ	0.621711	0.1167	a
Uridine nucleosidase	DZ	0.678886	0.074589	a
Uridine nucleosidase	EZ	0.533611	0.191137	a

Uridine phosphorylase	AZ	0.829347	0.056455	a
Uridine phosphorylase	BZ	0.891435	0.016657	a
Uridine phosphorylase	CZ	-0.10454	0.146067	b
Uridine phosphorylase	DZ	-0.68785	0.163947	b
Uridine phosphorylase	EZ	-0.92838	0.216561	b
Uroporphyrinogen decarboxylase	AZ	0.866975	0.103291	a
Uroporphyrinogen decarboxylase	BZ	0.963014	0.033401	a
Uroporphyrinogen decarboxylase	CZ	-0.38104	0.172209	b
Uroporphyrinogen decarboxylase	DZ	-0.76706	0.109521	b
Uroporphyrinogen decarboxylase	EZ	-0.6819	0.206604	b
Uroporphyrinogen-III C-methyltransferase	AZ	0.866659	0.102881	a
Uroporphyrinogen-III C-methyltransferase	BZ	0.962763	0.033284	a
Uroporphyrinogen-III C-methyltransferase	CZ	-0.38295	0.172962	b
Uroporphyrinogen-III C-methyltransferase	DZ	-0.76987	0.110636	b
Uroporphyrinogen-III C-methyltransferase	EZ	-0.6766	0.205898	b
Uroporphyrinogen-III synthase	AZ	-0.85779	0.153122	b
Uroporphyrinogen-III synthase	BZ	-0.98338	0.115376	b
Uroporphyrinogen-III synthase	CZ	0.570972	0.101287	a
Uroporphyrinogen-III synthase	DZ	0.784279	0.060862	a
Uroporphyrinogen-III synthase	EZ	0.485918	0.212781	a
UTP--glucose-1-phosphate uridylyltransferase	AZ	0.895487	0.082605	a
UTP--glucose-1-phosphate uridylyltransferase	BZ	0.980742	0.024111	a
UTP--glucose-1-phosphate uridylyltransferase	CZ	-0.39229	0.166334	b
UTP--glucose-1-phosphate uridylyltransferase	DZ	-0.7713	0.109061	b
UTP--glucose-1-phosphate uridylyltransferase	EZ	-0.71263	0.204973	b
Valine--pyruvate transaminase	AZ	-0.95521	0.160654	b
Valine--pyruvate transaminase	BZ	-0.80353	0.10269	b
Valine--pyruvate transaminase	CZ	0.464303	0.103315	a
Valine--pyruvate transaminase	DZ	0.823043	0.065305	a
Valine--pyruvate transaminase	EZ	0.471399	0.234611	a
Valine--tRNA ligase	AZ	0.868536	0.09196	a
Valine--tRNA ligase	BZ	0.970388	0.025544	a
Valine--tRNA ligase	CZ	-0.37212	0.159382	b
Valine--tRNA ligase	DZ	-0.85457	0.136848	b
Valine--tRNA ligase	EZ	-0.61223	0.199451	b
Vesicle-fusing ATPase	AZ	0.876062	0.101512	a
Vesicle-fusing ATPase	BZ	0.94001	0.039625	a
Vesicle-fusing ATPase	CZ	-0.34925	0.170521	b
Vesicle-fusing ATPase	DZ	-0.75032	0.102407	b
Vesicle-fusing ATPase	EZ	-0.71651	0.214683	b
Xaa-Pro aminopeptidase	AZ	0.858847	0.100336	a
Xaa-Pro aminopeptidase	BZ	0.93918	0.036538	a
Xaa-Pro aminopeptidase	CZ	-0.23374	0.14456	b
Xaa-Pro aminopeptidase	DZ	-0.82174	0.13256	b

Xaa-Pro aminopeptidase	EZ	-0.74255	0.212322	b
Xaa-Pro dipeptidase	AZ	-0.80961	0.10241	b
Xaa-Pro dipeptidase	BZ	-0.96061	0.027449	b
Xaa-Pro dipeptidase	CZ	0.21074	0.094558	a
Xaa-Pro dipeptidase	DZ	0.815378	0.177046	a
Xaa-Pro dipeptidase	EZ	0.744093	0.215441	a
Xenobiotic-transporting ATPase	AZ	-0.92242	0.116281	b
Xenobiotic-transporting ATPase	BZ	-0.95303	0.151276	b
Xenobiotic-transporting ATPase	CZ	0.644053	0.114258	a
Xenobiotic-transporting ATPase	DZ	0.685134	0.076007	a
Xenobiotic-transporting ATPase	EZ	0.546267	0.1941	a
Xylan 1,4-beta-xylosidase	AZ	-0.90228	0.093958	b
Xylan 1,4-beta-xylosidase	BZ	-1.11411	0.067727	b
Xylan 1,4-beta-xylosidase	CZ	0.750669	0.102698	a
Xylan 1,4-beta-xylosidase	DZ	0.632909	0.091445	a
Xylan 1,4-beta-xylosidase	EZ	0.632814	0.191525	a
Xyloglucan:xyloglucosyl transferase	AZ	-0.94946	0.199276	c
Xyloglucan:xyloglucosyl transferase	BZ	-0.61602	0.088585	bc
Xyloglucan:xyloglucosyl transferase	CZ	0.79821	0.126691	a
Xyloglucan:xyloglucosyl transferase	DZ	0.33424	0.207183	ab
Xyloglucan:xyloglucosyl transferase	EZ	0.433031	0.150332	ab
Xylulokinase	AZ	0.802738	0.081857	a
Xylulokinase	BZ	0.857514	0.041326	a
Xylulokinase	CZ	-0.60288	0.182417	b
Xylulokinase	DZ	-0.32889	0.189908	b
Xylulokinase	EZ	-0.72847	0.207724	b

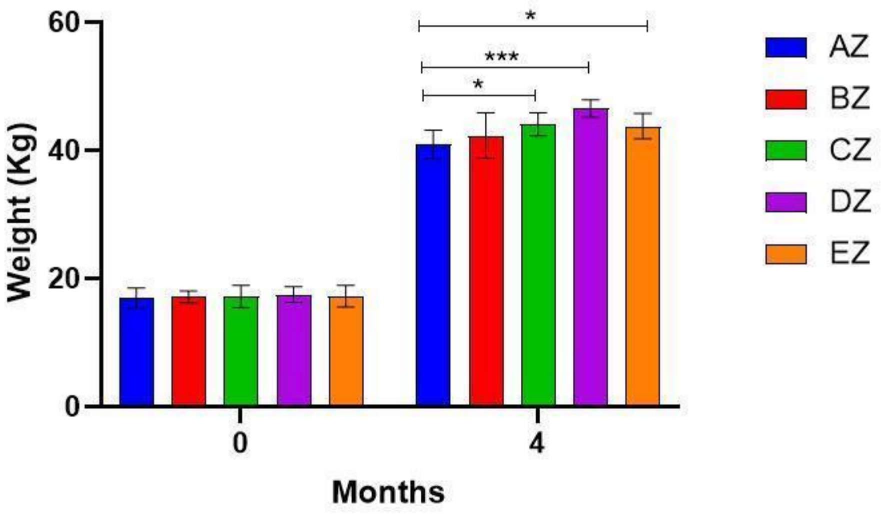

Figure S1. Body weights of the ST sheep in different groups. Significance is presented as * $p < 0.05$ and *** $p < 0.001$; data are presented as the mean ± SEM (n = 6).

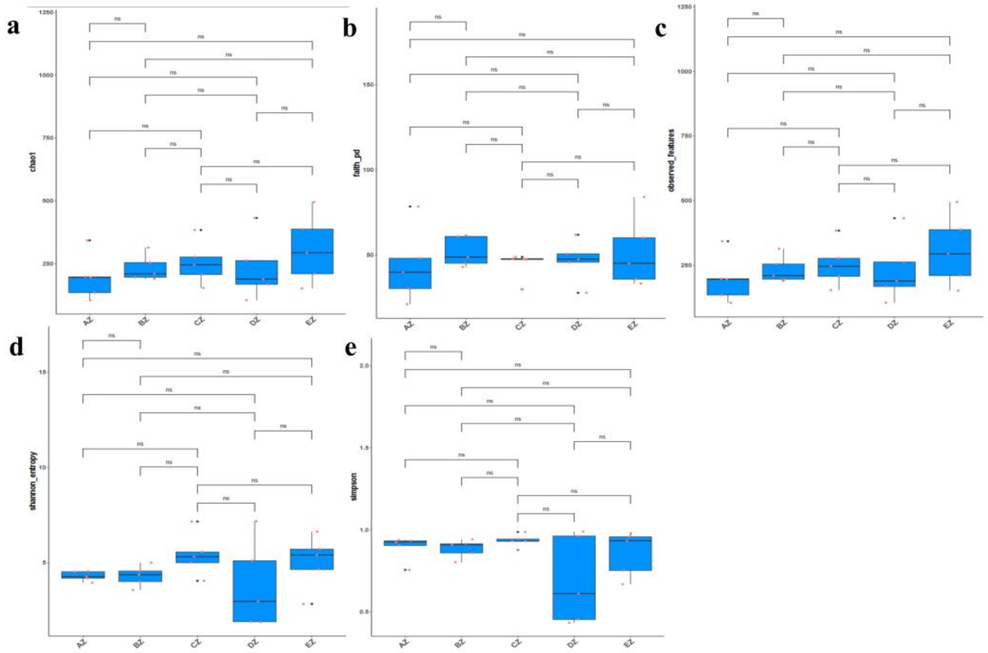

Figure S2. Comparing analyses of the Alpha diversity index in the ST sheep groups: (**a**) chao1; (**b**) faith_pd; (**c**) observed_features; (**d**) shannon_entropy; (**e**) Simpson. ns: non-significant

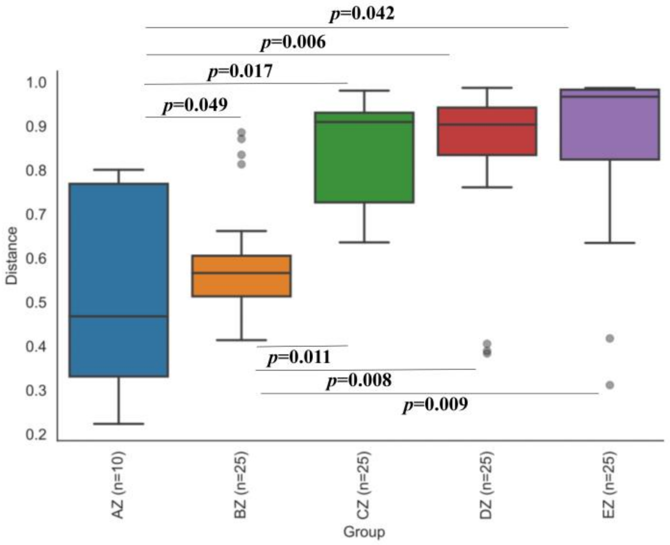

Figure S3. Pairwise ANOSIM analysis of Qiime2 in the five ST sheep groups.
